# Genetic validation of *Aspergillus fumigatus* phosphoglucomutase as a viable therapeutic target in invasive aspergillosis

**DOI:** 10.1016/j.jbc.2022.102003

**Published:** 2022-04-30

**Authors:** Kaizhou Yan, Mathew Stanley, Bartosz Kowalski, Olawale G. Raimi, Andrew T. Ferenbach, Pingzhen Wei, Wenxia Fang, Daan M.F. van Aalten

**Affiliations:** 1 Centre for Gene Regulation and Expression, School of Life Sciences, University of Dundee, Dundee, United Kingdom; 2 National Engineering Research Center for Non-Food Biorefinery, Guangxi Academy of Sciences, Nanning, China

**Keywords:** Aspergillus fumigatus, phosphoglucomutase, covalent inhibitor, isothiazolone, antifungal, fragment, FBDD, *Af*PGM, *Aspergillus fumigatus* PGM, BLI, biolayer interferometry, *Ca*PGM, *Candida albicans* PGM, CM, complete media, COVID-19, coronavirus disease 2019, G6PDH, Glc-6P dehydrogenase, Glc-1,6-2P, glucose-1,6-bisphosphate, Glc-1P, glucose-1-phosphate, Glc-6P, glucose-6-phosphate, *Hs*PGM, *Homo sapiens* PGM, ISFP1, isothiazolone fragment of PGM, MM, minimal media, Mw, molecular weight, *Pa*PGM, *Pseudomonas aeruginosa* PGM, PGM, phosphoglucomutase, UDP-Glc, UDP-glucose

## Abstract

*Aspergillus fumigatus* is the causative agent of invasive aspergillosis, an infection with mortality rates of up to 50%. The glucan-rich cell wall of *A. fumigatus* is a protective structure that is absent from human cells and is a potential target for antifungal treatments. Glucan is synthesized from the donor uridine diphosphate glucose, with the conversion of glucose-6-phosphate to glucose-1-phosphate by the enzyme phosphoglucomutase (PGM) representing a key step in its biosynthesis. Here, we explore the possibility of selectively targeting *A. fumigatus* PGM (*Af*PGM) as an antifungal treatment strategy. Using a promoter replacement strategy, we constructed a conditional *pgm* mutant and revealed that *pgm* is required for *A. fumigatus* growth and cell wall integrity. In addition, using a fragment screen, we identified the thiol-reactive compound isothiazolone fragment of PGM as targeting a cysteine residue not conserved in the human ortholog. Furthermore, through scaffold exploration, we synthesized a para-aryl derivative (ISFP10) and demonstrated that it inhibits *Af*PGM with an IC_50_ of 2 μM and exhibits 50-fold selectivity over the human enzyme. Taken together, our data provide genetic validation of PGM as a therapeutic target and suggest new avenues for inhibiting *Af*PGM using covalent inhibitors that could serve as tools for chemical validation.

Every year more than two million patients worldwide suffer infections from pathogenic fungi (1). The fungal species of *Candida* spp., *Aspergillus* spp., and *Cryptococcus* spp. are the main agents of fungal pathogenesis. *Aspergillus fumigatus* is a fungal species that is widespread in the environment, rarely infecting healthy individuals (2). However, *A. fumigatus* causes life-threatening invasive aspergillosis in immunocompromised individuals such as HIV-infected patients (1). Furthermore, increasing use of immunosuppressants enhances the risk for aspergillosis infections in organ-transplant recipients (3). Chemotherapy for cancer patients targets both cancer cells and neutrophils, which weakens the host defense against aspergillosis (2). Moreover, *A. fumigatus* also infects immunocompetent individuals with other medical comorbidities that enhance their risk of sensitization toward fungal infection (4). For instance, invasive pulmonary aspergillosis was recently reported in patients with severe influenza (5). Furthermore, approximately 30% patients with coronavirus disease 2019 (COVID-19) also developed invasive pulmonary aspergillosis (6, 7, 8). Although it is not clear whether aspergillosis is a major coinfection among COVID-19 patients, invasive pulmonary aspergillosis is believed to be a possible complication (9). Overall, *A. fumigatus* is estimated to lead to 600,000 deaths annually (10). The mortality rate of invasive aspergillosis remains approximately 50% even in cases where medical treatment is given (1, 11).

Clinical drugs against *A. fumigatus* are limited to only a few compound classes (echinocandins, azoles, polyenes) (12). Furthermore, these antifungals are facing emerging resistance (13), toxicity (14), and undesirable drug–drug interactions (15). The improvement of existing antifungal drugs has only modestly progressed and only partially addresses these issues (16). For instance, while the toxicity of amphotericin B is reduced when produced in the monomeric form, emerging resistance is still not effectively addressed by this new type of formulation (16, 17). Furthermore, no effective vaccine is available to protect individuals from *A. fumigatus* infection (1). Therefore, the current situation represents a considerable clinical threat due to the dearth of antifungal pipelines. Despite a £2.6 billion investment for antifungal research over a 14 year period (18), only limited numbers of novel antifungals are currently undergoing phase II trials (*e.g.*, NCT03583164 and Ibrexafungerp) (19). One of the main challenges to overcome is the lack of new and well-characterized antifungal targets, which is one of the reasons that the pharmaceutical industry has largely ceased research and development on antifungal agents (20).

The fungal cell wall is the outermost layer of fungal cells and provides mechanical strength to maintain shape and protect against the environment (21). Previous studies have demonstrated that the cell wall is essential for fungal viability, morphogenesis, and virulence (21, 22, 23). The cell wall is absent from human cells and as such has long been considered to be an attractive antifungal target. The *A. fumigatus* cell wall consists of polysaccharides and proteins (21, 24, 25, 26, 27, 28, 29). In *A. fumigatus* hyphae, cell wall polysaccharides include chitin, glucan, galactomannan, and galactosaminoglycan (21, 24, 30, 31, 32). β-1,3-glucan is a major carbohydrate in the *A. fumigatus* cell wall (25). Previous studies have demonstrated that β-1,3-glucan is essential for fungal growth and cell wall integrity (33, 34, 35). β-1,3-glucan is synthesized from UDP-glucose (UDP-Glc) by β-1,3-glucan synthase complex, a membrane-embedded protein that is composed of a catalytic subunit (Fks1, EC 2.4.1.34) and a regulatory subunit (Rho GTPase) (36, 37, 38, 39, 40, 41, 42). As such, targeting Fks1 elicits antifungal activity, in agreement with the clinical use of echinocandins, lipopeptides that inhibit the activity of Fks1 (43, 44, 45, 46). Although echinocandins are the third-line antifungal agents for the treatment of aspergillosis, the emergence of echinocandin-resistant strains hinders their clinical use in antifungal therapy (12, 47, 48, 49).

As the activity of β-1,3-glucan synthase requires the continuous supply of UDP-Glc, the inhibition of UDP-Glc biosynthesis is likely to modulate the biosynthesis of cell wall β-1,3-glucan and in turn disrupt cell wall integrity. Moreover, UDP-Glc is also involved in the biosynthetic pathway of galactomannan, α-1,3-glucan, and galactosaminoglycan in the *A. fumigatus* cell wall (50, 51, 52). These three carbohydrates are essential for the viability and/or virulence of *A. fumigatus* (31, 51, 53, 54, 55, 56, 57). In addition to affecting cell wall carbohydrates, targeting UDP-Glc biosynthesis may also affect other essential biological processes including trehalose biosynthesis and *N*-glycosylation (58, 59, 60, 61, 62). Taken together, targeting UDP-Glc biosynthesis is likely to elicit antifungal activity through disruption of cell wall integrity, *N*-glycosylation, and trehalose biosynthesis. As such, disruption of UDP-Glc biosynthesis could be hypothesized to be a likely Achilles’ heel of *A. fumigatus*.

The UDP-Glc biosynthetic pathway starts from the phosphorylation of glucose by hexokinase (EC 2.7.1.1), yielding glucose-6-phosphate (Glc-6P), which is subsequently converted to glucose-1-phosphate (Glc-1P) by phosphoglucomutase (PGM) (EC 5.4.2.2). UDP-Glc pyrophosphorylase (EC 2.7.7.9) then converts Glc-1P and UTP to UDP-Glc and pyrophosphate. As such, the inhibition of PGM activity could hypothetically limit the supply of Glc-1P and in turn disrupt the biosynthesis of UDP-Glc, suggesting that PGM is a possible antifungal target.

To select potential enzymatic protein targets, Wyatt *et al*. have raised six criteria: essentiality, druggability, assay feasibility, toxicity, resistance potential, and structural information (63). Target essentiality is a key prerequisite of drug discovery campaigns. The identification of target essentiality is carried out through genetic and chemical validation. Although genetic studies in *Ganoderma lucidum* have demonstrated that PGM is essential for fungal growth and cell wall integrity (64), physiological functions of *A. fumigatus* PGM (*Af*PGM) have not been explored. Therefore, no genetic validation of *Af*PGM has been reported.

To date, chemical validation of *Af*PGM has been lacking due to the absence of suitable chemical tools (*e.g.*, small molecule inhibitors). Currently, PGM inhibitors are limited to mechanism-inspired inhibitors based upon the sugar phosphate structure of the enzyme substrate (65, 66, 67). The possession of negatively charged phosphate groups reduces cell membrane permeability of such inhibitors (68, 69, 70), reducing the attractiveness of mechanism-inspired inhibitors for further follow-up studies. Currently, no other types of inhibitors against PGM have been reported. As such, chemical validation of *Af*PGM is hampered by the lack of suitable tool inhibitors. Moreover, the PGM ortholog in human (*Hs*PGM) is indispensable for human health as missense mutations in *Hs*PGM lead to congenital disorders of glycosylation (71). To avoid toxicity, any putative PGM inhibitor must exhibit exquisite selectivity to *Af*PGM over *Hs*PGM. *Af*PGM shares 53% sequence similarity compared to *Hs*PGM and, therefore, achieving selectivity of inhibitors can only be achieved with the help of a high-resolution crystal structure of *Af*PGM. PGM proteins belong to the α-phosphohexomutase family, members of which exhibit a heart-shaped structure composed of four domains (I, II, III, and IV) (71, 72, 73, 74, 75). These four domains form the active site in which the reaction is carried out *via* a “flip” mechanism (76, 77, 78). Prior to enzymatic catalysis, a catalytic serine is phosphorylated in cells (79). The 1-OH of Glc-6P undergoes nucleophilic attack of the phosphate group conjugated to the catalytic serine. As such, the phosphate group transfers to the 1-OH *via* S_N_2 substitution, which forms glucose-1,6-bisphosphate (Glc-1,6-2P) (78, 80). Next, Glc-1,6-2P flips 180˚ in the active site (81), placing the 6-phosphate group adjacent to the catalytic serine (77). Next, the 6-phosphate group transfers to the catalytic serine *via* the same mechanism as the 1-phosphate group (76). As such, Glc-6P is converted to Glc-1P. The enzymatic catalysis is facilitated by a conformational change (“open” to “closed”) of the protein through motion of domain IV *via* a hinge (77, 82, 83). Although the structure of PGM has been extensively studied in several eukaryotes, the absence of the *Af*PGM structure hampers discovery of inhibitors that are potentially selective to *Af*PGM over *Hs*PGM and in turn hinders the chemical validation of *Af*PGM as a potential antifungal target.

Here, we show that PGM is essential for the growth of *A. fumigatus*, which serves as the first genetic validation of *Af*PGM as an antifungal target. Furthermore, through fragment-based discovery of a thiol-reactive compound isothiazolone fragment of PGM (ISFP1) and X-ray crystallography, we demonstrate that the enzyme activity of *Af*PGM is inhibited through modification of a cysteine (C353) that is absent from *Hs*PGM. Exploration of the scaffold leads to ISFP10, a compound with an *IC*_*50*_ of 2 μM and 50-fold selectivity over the human enzyme. Our results show the first covalent inhibition mechanism in enzymes of the α-phosphohexomutase family, which serves as a starting point for exploring and establishing tools for the chemical validation of *Af*PGM.

## Results

### *A. fumigatus* possesses an active PGM enzyme

Genomic annotation suggests that *A. fumigatus* possesses a gene coding for PGM (GenBank: AFUA_3G11830) although there is no experimental evidence to support this function (84). The *pgm* gene possesses two introns in the ORF. An intron-free ORF of *pgm* was amplified from *A. fumigatus* RNA. The *pgm* gene was expressed as a 6His-modified GST fusion (6His-GST-PGM) protein in *Escherichia coli* and purified by glutathione sepharose beads. PGM was cleaved off the beads and further purified by size-exclusion chromatography (Fig. S1*A*). The main peak showed a single band in SDS-PAGE with a molecular weight (M_w_) between 55 kDa and 72 kDa, in agreement with the M_w_ of an *Af*PGM monomer (61 kDa, calculated by ExPASy) (Fig. S1*A*). Size-exclusion chromatography demonstrated that the main peak corresponded to an M_w_ of 63 kDa, therefore, *Af*PGM is likely to be a monomer in solution. Intact protein mass spectrometry showed that there were two protein species in the peak fraction (Fig. S1*A*). The M_w_ difference (82 Da) between the two protein species likely corresponds to the M_w_ of a phosphoryl group (79 Da), which is compatible with the observation that intracellular ATP/Glc-1,6-2P phosphorylates a catalytic serine in the active site of phosphohexomutases (78, 79, 85). The enzymatic activity of *Af*PGM for Glc-1P was determined using a Glc-6P dehydrogenase (G6PDH) coupled enzyme assay. *Af*PGM exhibits a *K*_m_ of 16 μM and a *k*_cat_ of 7 s^−1^ (Fig. S1*B*). The catalytic efficiency of *Af*PGM is 4 − 10^5^ s^−1^ M^−1^, in agreement with the catalytic efficiency of *Pseudomonas aeruginosa* PGM (*Pa*PGM) (2 × 10^6^ s^−1^ M^−1^) and *Hs*PGM (2 × 10^6^ s^−1^ M^−1^) (Table 1). Taken together, these data show that *A. fumigatus* possesses an active PGM enzyme.

**Table 1:** Kinetic parameters for *Af*PGM and orthologs from other organisms

Organism	*K*_m_ (μM)	*k*_cat_ (s^−1^)	*k*_cat_/*K*_m_ (s^−1^ M^−1^)	Reference
*Aspergillus fumigatus*	12.0 ± 7.0	6.7 ± 0.7	6 × 10^5^	This work
*Pseudomonas aeruginosa*	5.4 ± 0.3	8.2 ± 0.2	2 × 10^6^	(168)
*Homo sapiens*	80 ± 4	143 ±2	2 × 10^6^	(71)

Data are shown as mean ± SD for three determinations.

### PGM is essential for *A. fumigatus* viability and cell wall integrity

**Figure 1:**
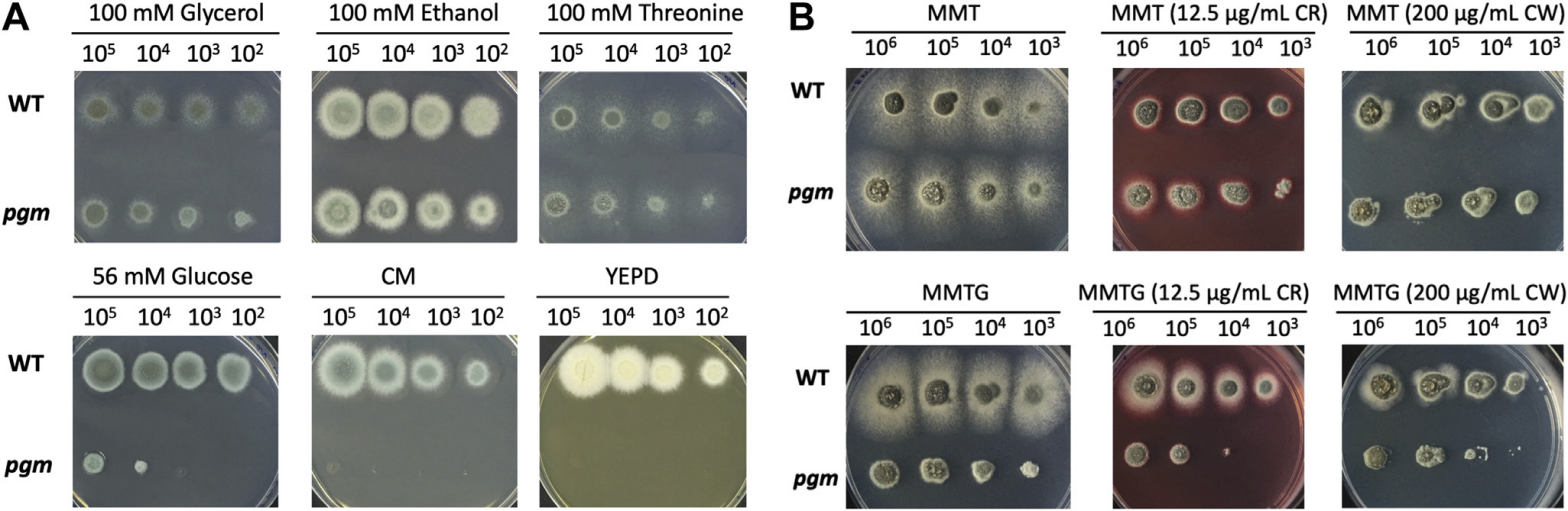
**Phenotypic analysis of the pgm conditional mutant strains in *Aspergillus fumigatus***. *A*, growth of the pgm conditional mutant strain on solid agar media supplemented with different carbon sources (glycerol, ethanol, threonine, glucose) and CM or YEPD media. The number of spores ranges from 10^5^ to 10^2^. *B*, the *upper panel* shows the growth of the *pgm* conditional mutant strain on solid minimal media supplemented with 100 mM threonine (MMT) and calcofluor white (CW) or Congo red (CR). The *lower panel* shows the growth of the *pgm* conditional mutant strain on solid minimal media supplemented with 100 mM threonine and 6 mM glucose (MMTG) and CW or CR. The number of spores ranges from 10^6^ to 10^3^. CM, complete media; PGM, phosphoglucomutase.

To explore possible essentiality of *pgm* in *A. fumigatus*, we initially attempted to construct a *pgm* deletion mutant through replacement of *pgm* with a *pyrG* cassette. However, all the screened transformants were negative by diagnostic PCR. Therefore, a conditional mutant was constructed through the replacement of the native *pgm* promoter by the alcohol dehydrogenase promoter (P_*alcA*_) from *Aspergillus nidulans* (Fig. S2*A*). P_*alcA*_ is a tightly regulated promoter that can be induced by the presence of alcohol, glycerol, and threonine (86, 87) and repressed with glucose (86, 87) and YEPD media (87). To construct the *pgm* conditional mutant, a plasmid (pALPGMN) containing a selective marker (*pyr-4*) and P_*alcA*_ fused with the 3′ truncated *pgm* was transformed into protoplasts of the *A. fumigatus* KU80 Δ*pyrG*^*-*^ strain to promote homologous recombination (Fig. S2*A*). The construction of the conditional mutant strain was confirmed by PCR (Fig. S2*B*) and Southern blot (Fig. S2*C*). One of the potential mutant strains (Fig. S2*C*, No. 25) was fully correct, named as *P*_*alcA*_*:*:*pgm* and utilized for phenotypic analysis. Growth of *P*_*alcA*_*:*:*pgm* was the same as the WT strain on solid minimal media (MM) supplemented with 100 mM glycerol, 100 mM ethanol, and 100 mM threonine as the sole carbon sources, respectively (Fig. 1*A*). However, the *P*_*alcA*_*:*:*pgm* strain showed retarded growth in MM media containing 56 mM glucose and growth was fully inhibited on complete media (CM) and YEPD (Fig. 1*A*). On MM solid media supplemented with 100 mM threonine and 6 mM glucose, the growth of the conditional mutant strain was partially inhibited (Fig. 1*B*). These results suggest that repression of P_*alcA*_ can inhibit the growth of the *P*_*alcA*_*:*:*pgm* strain, demonstrating that *pgm* is essential for the viability of *A. fumigatus* under the conditions investigated. To investigate the role of *pgm* in cell wall integrity, the susceptibility of strains to chemical reagents (Calcofluor white and Congo red), which interact with chitin and glucan in the cell wall (88, 89), were determined under inductive and partially repressive conditions (Fig. 1*B*). Under partially repressive conditions, the *P*_*alcA*_*:*:*pgm* strain exhibited increased sensitivity to the cell wall perturbing reagents, suggesting that repression of *pgm* results in a defect in cell wall integrity (Fig. 1*B*). Taken together, these *in vitro* experiments show that the *pgm* gene is essential for viability and cell wall integrity of *A. fumigatus*.

### The mode of substrate-recognition is conserved in *Af*PGM and *Hs*PGM

**Figure 2:**
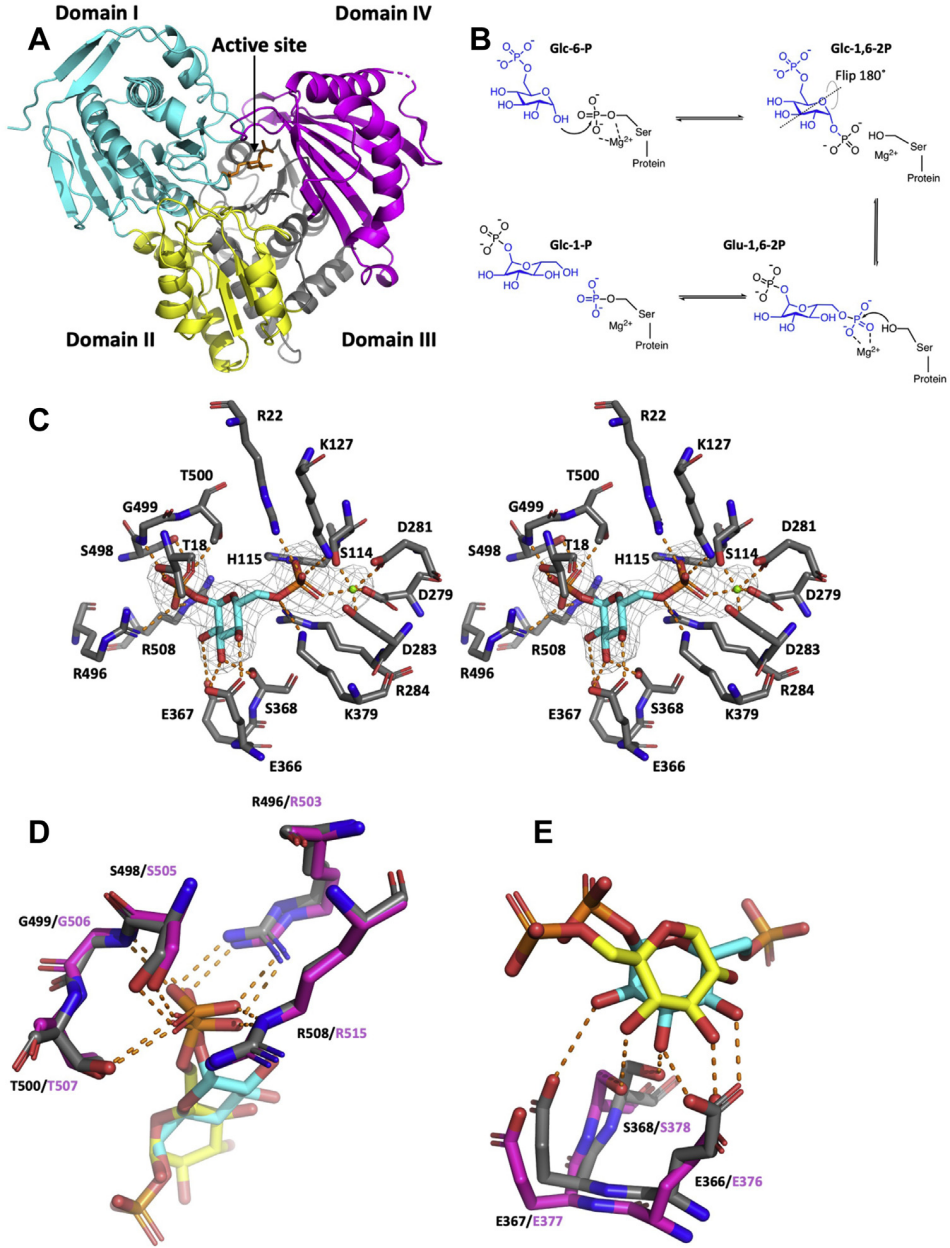
**Crystal structures and catalytic mechanism of *Af*PGM**. *A*, an overview of the *Af*PGM structure. Domains I-III are colored in *cyan*, *yellow*, and *gray*, respectively. Domain IV is colored in *magenta*. The reaction intermediate (Glc-1,6-2P) in the active site is shown as *orange sticks*. *B*, the proposed catalytic mechanism of *Af*PGM as adapted from previous work (72). *C*, a close-up view of the *Af*PGM active site. Carbon atoms of the protein and the intermediate (Glc-1,6-2P) are colored in *gray* and *cyan*, respectively. The *green sphere* indicates a Mg^2+^ ion. The *gray mesh* around the intermediate indicates F_o_-F_c_ map before inclusion of ligand contoured at 2.5σ. *Orange dashed lines* indicate polar interactions. Images are shown as stereoscopic view. *D*, superposition of the phosphate-binding site of *Af*PGM (*gray sticks*) onto that of *Hs*PGM (PDB code 6UIQ: *magenta* sticks) in complex with Glc-6P (*yellow* sticks). *E*, superposition of the sugar-binding site of *Af*PGM (*gray sticks*) onto that of *Hs*PGM (PDB code 6UIQ; *magenta* sticks) complexed with Glc-6P (*yellow sticks*). *Af*PGM, *Aspergillus fumigatus* PGM; Glc-1,6-2P, glucose-1,6-bisphosphate; *Hs*PGM, *Homo sapiens* PGM; Glc-6P, glucose-6-phosphate; PGM, phosphoglucomutase.

As the structure of *Af*PGM has not yet been reported, we next sought to solve the crystal structure of *Af*PGM. Purified *Af*PGM was incubated with Glc-6P and Mg^2+^ ions and crystallized from PEG solutions. Diffraction data were collected at the European Synchrotron Radiation Facility, the phase problem was solved by molecular replacement using the structure of *Hs*PGM (PDB code 5EPC) (Stiers *et al*., 2016) as the search model and the structure of *Af*PGM was refined to 2.48 Å (R = 0.20, R_free_ = 0.26 Table 2). The overall structure of *Af*PGM exhibits a heart shape with four domains (Fig. 2*A*). Domains I-III are Rossmann folds with a central β-sheet flanked by α-helices (Fig. 2*A*). Domain IV exhibits an α+β fold and is linked to domain III *via* a hinge region (Fig. 2*A*). Although the overall structure of *Af*PGM is similar to that of the human ortholog (Cα RMSD = 1.3 Å; PDB code 5EPC) (71), *Af*PGM adopts a “closed” conformation compared to reported *Hs*PGM structures (Fig. S3). During initial stages of the refinement, a well-defined F_o_-F_c_ map revealed the presence of the reaction intermediate Glc-1,6-2P in the active site of *Af*PGM (Fig. 2*C*), which we assume to have been converted from Glc-6P by the enzyme. The 6-phosphate group of the intermediate is oriented toward the hydroxyl group of S114. Sequence alignment (Fig. S7) shows that S114 corresponds to the conserved serine in the catalytic motif (TASHNP) of PGM, suggesting that S114 is the catalytic serine of *Af*PGM (Fig. 2*C*). The 6-phosphate group is adjacent (2.5 Å) to the hydroxyl group of S114, which suggests that the intermediate exhibits a near attack conformation. By superimposing the structure of *Af*PGM onto that of *Hs*PGM, we observed that the 6-phosphate group occupies the same position as the phosphate group on the catalytic serine before phosphoryl transfer (Fig. S4). Two apical positions are occupied by oxygens from the catalytic serine (S135 in *Hs*PGM) and the 6-phosphate group of the intermediate (Fig. S4). Equatorial positions are occupied by oxygens from two phosphate groups (S135 and the intermediate) (Fig. S4). Therefore, the conformation of the two phosphate groups mimics a pentavalent phosphorus formed in the transition state of S_N_2 substitution (90). As such, the intermediate is likely to represent a snapshot that is close to the transition state of phosphoryl transfer. This hypothesis is supported by the fact that the 6-phosphate group and S114 are covered by continuous electron density (Fig. S5). Furthermore, the movement of domain I enables R22, a residue on a β-strand in domain I, to coordinate the 6-phosphate group in *Af*PGM, which is likely to stabilize the transition state of phosphoryl transfer (Fig. S4). By contrast, R41 (counterpart of R22 in *Af*PGM) does not interact with the phosphate group on the catalytic serine in *Hs*PGM since domain I adopts a different conformation (Fig. S4). As such, the motion of domain I is likely to stabilize the transition state through R22 (Fig. S4), in agreement with the induced-fit model. A model of the proposed motion of domain I is provided (Movie S1). The hypothesized function of R22 is supported by studies in *Pa*PGM in which mutation of R20 (counterpart of R22 in *Af*PGM) to alanine reduces the enzyme activity (91). A spherical electron density map was observed adjacent to this serine–—the reaction intermediate—and the carboxyl groups of three aspartates (D279, D281, D283) (Fig. 2*C*). Sequence alignment (Fig. S7) shows that the three aspartates (D279, D281, D283) correspond to aspartates in the metal binding motif of PGM (DGDGDR), suggesting that the spherical electron density map likely represents a metal ion. The putative metal ion coordinates to five oxygens with an average distance (metal–oxygen) of 2.1 Å, in agreement with the coordination feature of magnesium ion (coordination number is six or five; average Mg-O distance is 2.1 Å) (92, 93, 94). As such, the spherical electron density map likely represents a magnesium ion (Mg^2+^).

**Table 2:** Summary of X-ray diffraction data and refinement

Parameters	Native *Ca*PGM	*Ca*PGM-ISFP1	*Af*PGM-Glc-1,6-2P
**PDB code**	7PIZ	7PJC	7P5O
**Space group**	P2_1_	P2_1_	P2_1_2_1_2
**Cell dimensions**			
a, b, c (Å)	67.0, 86.8, 109.9	66.7, 86.4, 110.2	98.6, 209.6, 61.2
α, β, γ (˚)	90.0, 92.8, 90.0	90.0, 92.7, 90.0	90.0, 90.0, 90.0
**R**_**merge**_**(%)**^a^	14.5 (33.6)	11.3 (49.5)	10.6 (64.5)
**CC (1/2) (%)**	97.9 (82.0)	99.3 (85.5)	99.4 (79.5)
**Completeness (%)**	95.5 (94.3)	93.0 (70.4)	98.4 (99.3)
**Redundancy**	3.4 (3.4)	3.2 (3.4)	4.2 (4.3)
**Resolution range (Å)**	25.7–2.2	68.0–2.1	89.2–2.5
**No. of observations**	220,976 (14,671)	151,483 (8099)	189,268 (9779)
**No. of unique**	65,230 (4312)	47,519 (2376)	44,960 (2259)
**I/σ(I)**	4.5 (2.3)	4.0 (1.4)	11.2 (2.1)
**R (%)**^b^	19.6	18.9	20.11
**R**_**free**_**(%)**^c^	25.9	25.5	25.84
**No. of atoms**			
Protein	8517	8501	8558
Water	1428	627	510
SO_4_^2-^	25	50	ND
Glycerol	ND	6	ND
Adduct	ND	28	ND
Glc-1,6-2P	ND	ND	40
Mg^2+^	ND	ND	2
**B-factors (Å**^**2**^**)**			
Protein	20.2	31.4	40.9
Water	27.1	30.6	35.7
SO_4_^2-^	41.7	73.8	ND
Glycerol	ND	32.8	ND
Adduct	ND	28.8	ND
Glc-1,6-2P	ND	ND	32.0
Mg^2+^	ND	ND	17.4
**RMSD**^d^**from ideal geometry**			
Bond length (Å)	0.008	0.008	0.009
Bond angle (Å)	1.0	1.0	1.6

Numbers in parenthesis indicate outer shell data. ND indicates not detected.

a $R_{merge}=\frac{\sum_{hkl}\sum_{i=1}^{n}\left|I_{i}\left(hkl\right)-\bar{I}\left(hkl\right)\right|}{\sum_{hkl}\sum_{i=1}^{n}I_{i}\left(hkl\right)}$. *I*_*i*_ indicates intensity observed by experiments.

b $R=\frac{\sum_{hkl}\left|F_{obs}\left(hkl\right)-F_{cal}\left(hkl\right)\right|}{\sum_{hkl}F_{obs}\left(hkl\right)}$ The observed structure factor amplitude is *F*_*obs*_. The calculated structure factor amplitude is *F*_*cal*_. A proportion (5%) of *F*_*obs*_ was used to calculate.

c *R*_*free*_ are not utilized for model building.

d RMSD is the abbreviation of root mean square deviation.

To compare the substrate-recognition mode with that of *Hs*PGM, the structure of *Af*PGM was superimposed onto that of *Hs*PGM in complex with Glc-6P (Fig. 2) (95). The sugar ring (3-OH and 4-OH) of the intermediate is recognized by E366 (Fig. 2*E*). S368 also interacts with the sugar ring through a hydrogen bond (Fig. 2*E*). Residues for sugar recognition are conserved in *Af*PGM and *Hs*PGM, suggesting that both PGMs adopt the same mode of sugar-recognition. The 1-phosphate group of the intermediate is bound to the phosphate binding motif (R496, S498, G499, T500, R508) (Fig. 2*D*). In addition to residues in the phosphate binding motif, T18 also forms a hydrogen bond with the 1-phosphate group. The hydrogen bond formed by T18 is unique to *Af*PGM and absent from *Hs*PGM (Fig. 2, *C* and *D*), although T18 (corresponding to T37 in *Hs*PGM) is highly conserved among PGM families (Fig. S7). The structure of *Af*PGM shows that T18 is situated in a loop within domain I (Fig. S4). This loop is postulated to move toward domain IV when *Af*PGM changes its conformation from “open” to “closed” (Movie S1). The movement of the loop is likely achieved by a combination of domain I motion and intrinsic loop flexibility (Movie S1). As such, the movement of the loop places T18 adjacent to the 1-phosphate group, facilitating the formation of a hydrogen bond with T18. This additional hydrogen bond interaction may stabilize the intermediate at the transition state of phosphoryl transfer. The function of T18 is supported by the fact that Y17A (counterpart of T18 in *Af*PGM) reduces the activity of *Pa*PGM (82). Taken together, the motion of domain I is likely necessary to stabilize the transition state of phosphoryl transfer through R22 and T18. As residues for substrate recognition are conserved in *Hs*PGM, both PGMs adopt the same mode of substrate-recognition.

### A thiol-reactive fragment modifies cysteines in *Af*PGM

**Figure 3:**
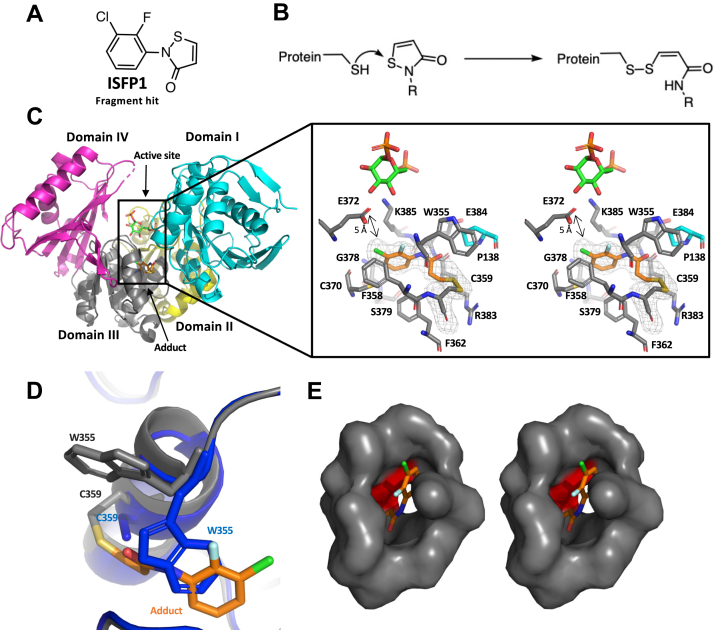
**Crystal structures of *Ca*PGM in complex with ISFP1 (fragment hit)**. *A*, the structure of the thiol-reactive fragment ISFP1, which contains a five-membered isothiazolone ring. *B*, the isothiazolone moiety can undergo a ring-opening reaction with the thiol group of cysteine side chain functionality, resulting in the formation of a disulphide covalent adduct (101). *C*, the *Ca*PGM–ISFP1 complex structure. Domains I-IV of *Ca*PGM are shown in *cyan*, *yellow*, *gray*, and *magenta*, respectively. The close-up view shows that ISFP1 (*orange sticks*) is covalently attached to the thiol group of *Ca*PGM C359. The formation of an isothiazolone disulphide adduct places the chlorine atom in proximity to E372 (5 Å).The *mesh* around the isothiazolone adduct indicates the F_o_-F_c_ map contoured at 2.5σ before inclusion of the ligand. Residues in domains I and III are shown in *cyan* and *gray*, respectively. *Green sticks* indicate the position of Glc-1,6-2P, as obtained by superposition with the *Af*PGM crystal structure. The close-up view is shown in stereoscopic view. *D*, in the *Ca*PGM–ISFP1 complex structure (*gray*), compared to the native *Ca*PGM structure (*blue*), W355 is flipped to the protein surface, forming a new pocket which is occupied by the ISFP1 adduct on C359. *E*, stereoscopic surface representation of the structure of *Ca*PGM in complex with ISFP1. Residues that are conserved in *Hs*PGM are shown as *gray* surface. *Ca*PGM F362 corresponding to M366 in *Hs*PGM is shown as *red* surface. *Ca*PGM, *Candida albicans* PGM; Glc-1,6-2P, glucose-1,6-bisphosphate; *Af*PGM, *Aspergillus fumigatus* PGM; PGM, phosphoglucomutase; *Hs*PGM, *Homo sapiens* PGM; ISFP1, isothiazolone fragment of PGM.

To identify compound tools for chemical validation of *Af*PGM, a fragment-based approach (96, 97) was applied by screening *Af*PGM against a Maybridge fragment compound library (1000 compounds) using biolayer interferometry (BLI) (98, 99, 100). One of the identified fragment hits (ISFP1) bound to *Af*PGM in a concentration-dependent manner (Fig. S6). In the association stage, binding of ISFP1 reached a plateau quickly (10 s). Within the dissociation stage, ISFP1 did not completely dissociate from *Af*PGM, suggesting that ISFP1 remains tightly associated with *Af*PGM. The structure of ISFP1 (Fig. 3*A*) contains a five-membered isothiazolone ring, which can covalently attach to the thiol group of cysteine side chains through the scission of the endocyclic N-S bond and the formation of a new disulphide bond to the cysteine thiol (Fig. 3*B*) (101). Sequence alignment shows that there are four cysteines in *Af*PGM (Fig. S7). Two cysteines (C242 and C364) are conserved in *Hs*PGM and the other two (C131 and C353) are absent from *Hs*PGM. Incomplete dissociation of ISFP1 was hypothesized to be due to covalent modification of cysteine side chains in *Af*PGM (Fig. S6). To test this, *Af*PGM was incubated with ISFP1 and analyzed using intact protein electrospray ionization mass spectrometry. Analysis of a preincubated *Af*PGM: ISFP1 solution (1:20) indicated modification of the protein by ISFP1 through observed increases in mass (232 Da) compared to the unmodified protein (60983 Da). This corresponds to modification of *Af*PGM with one ISFP1 adduct per protein molecule (Fig. S8). Furthermore, the M_w_ of modified *Af*PGM decreased to that of the unmodified *Af*PGM after treatment with DTT (Fig. S8), supporting ISFP1 forming a disulphide bond with cysteine thiols in *Af*PGM, a general isothiazolone mechanism which has been previously reported in the literature (102). As such, ISFP1 is a covalent modifier of *Af*PGM.

### ISFP1 inhibits *Af*PGM activity through covalent modification of C353

To unravel the mode of action of ISFP1, initially, we attempted to soak ISFP1 into *Af*PGM crystals to solve the *Af*PGM–ISFP1 complex structure. Unfortunately, we were not able to observe electron density for the compound. As an alternative strategy, we determined the crystal structure of ISFP1 in complex with the *Candida albicans* ortholog (*Ca*PGM). *Ca*PGM displays a high sequence similarity to *Af*PGM (66%). Diffraction data were obtained to a resolution of 2.1 Å (Table 2), the structure solved by molecular replacement and refined to 2.1 Å (Fig. 3*C* and Table 2). *Ca*PGM is structurally similar to *Af*PGM (RMSD 1.2 Å on Cα atoms) with two cysteines conserved (Fig. S7). During model building, well defined F_o_-F_c_ density was observed in the active site (Fig. 3*C*). Upon incorporation of the ISFP1 reaction product, the complex structure showed that ISFP1 forms a disulphide bond with C359 (Fig. 3*C*), which is conserved in PGM orthologs from several pathogenic fungi including *A. fumigatus*, *Pneumocystis jirovecii*, *Stachybotrys chartarum*, and *Histoplasma capsulatum* (Fig. S7). The C353 in *Af*PGM is located in a cleft (300 Å^3^) between domains I, III, and IV (Fig. S9*A*) and is solvent accessible (14 Å^2^ for the sulfur).

The adduct is bound to a pocket (282 Å^3^) in domain III *via* 268 Å^2^ buried surface area (Fig. 3*E*). Residues in the pocket are conserved in *Af*PGM (Fig. S7). The alkyl chain of the side chain of K385 interacts with the benzene ring of the adduct via a CH-π interaction (Fig. 3*C*) (103). The benzene ring of F362 (corresponding to M366 in *Hs*PGM) (Fig. 3*E*) also adopts a CH-π aromatic interaction to the benzene ring of the adduct (Fig. 3*C*) (104). Furthermore, the chlorine atom on the adduct is proximal (5 Å) to the carboxyl group of E372 (corresponding to E366 in *Af*PGM) (Fig. 3*C*), which is required for sugar ring recognition in the PGM active site (76). The electronegativity of the ISFP1 halogen substituents may perturb the side chain of E372, leading to disruption of sugar ring recognition, in agreement with the fact that E372 has a slightly perturbed orientation compared to that of native *Ca*PGM (Fig. S9*B*). This hypothesis is supported by the fact that both E372Q (*Ca*PGM) (Fig. S11*B*) and E366Q (*Af*PGM) (Fig. S11*A*) mutants are inactive even when the enzyme concentration was increased to 100 nM in the enzyme assay. Taken together, the complex structure of *Ca*PGM-ISFP1 suggests that ISFP1 is likely to inhibit the enzyme activity by perturbing sugar recognition, that is, decreasing the binding affinity toward the substrate.

An interesting aspect to covalent modification in this instance is the apparent formation of the fragment-binding pocket upon protein modification as the ISFP1 adduct–binding pocket is absent and occupied by a tryptophan (W355) in the native *Ca*PGM structure (Fig. 3*D*). The adduct mimics the indole ring of W355 and induces a flip of the tryptophan side chain to the interface between domains I and III (Figs. 3*D* and S10*A*). Neither W355 nor the adduct exhibit strong polar interactions with the protein molecule (Fig. 3*C*). Therefore, we speculate that the displacement of the indole ring of W355 is hydrophobically driven. As the indole ring of W355 is proximal (5 Å) to the active site (E372), the displacement of the indole ring of W355 concomitantly places the adduct proximal to the active site. The flip of W355 places the side chain in the interface region between domains I and III (Fig. S10*A*). The nitrogen on the indole ring of W355 forms a hydrogen bond with the backbone carbonyl of T21, which corresponds to T18 in *Af*PGM (Fig. S10*A*). This hydrogen bond may reduce mobility of the loop in which T21 presented, preventing T21 from interacting with the substrate. As previously described, the loss of the T21 (T18 in *Af*PGM) hydrogen bond is likely to destabilize the transition state of phosphoryl transfer and thus reduces the enzyme activity. Moreover, as the structure of *Ca*PGM–ISFP1 complex exhibits an “open” conformation, we sought to understand whether W355 hinders the motion of domain I when the enzyme changes its conformation. To do this, we attempted to obtain a “closed” *Ca*PGM structure by solving the crystal structure of *Ca*PGM in complex with its substrate, but no electron density was observed corresponding to bound substrate. As the structure of *Af*PGM–Mg^2+^-Glc-1,6-2P complex displays a “closed” conformation, alternatively, we obtained an homology model of the “closed” *Ca*PGM using SWISS-MODEL (105, 106, 107, 108, 109) using the structure of *Af*PGM-Mg^2+^-Glc-1,6-2P complex as the template. By superimposing the structure of the *Ca*PGM–ISFP1 complex onto that of the “closed” *Ca*PGM model, we observed that domains I and III move toward each other during the conformational change (Fig. S10*B*), in agreement with the fact that W355 ("open” *Ca*PGM) clashes with K356 and A139 in the “closed” *Ca*PGM (Fig. S10*B*). Therefore, the side chain of W355 likely introduces steric hindrance against the motion of domain I. As previously described, the movement of domain I potentially stabilizes the transition state *via* R22 in *Af*PGM (R25 in *Ca*PGM) (Fig. S4) and perturbing the motion of domain I is likely to destabilize the transition state and in turn reduces the catalytic activity of PGM, in agreement with the fact that a predicted allosteric pocket (predicted computationally by AlloSitePro) on *Ca*PGM encompasses the domains I&III interface (Fig. S10*C*) (110, 111). This hypothesis is also supported by the fact that the domain I&III interface has been predicted as a ligand-binding hot spot on *Pa*PGM (83). Taken together, the structure of the *Ca*PGM–ISFP1 complex also suggests that inhibitory action of ISFP1 may be a consequence of destabilization of the enzyme transition state and in turn reduce the rate of phosphoryl transfer.

**Figure 4:**
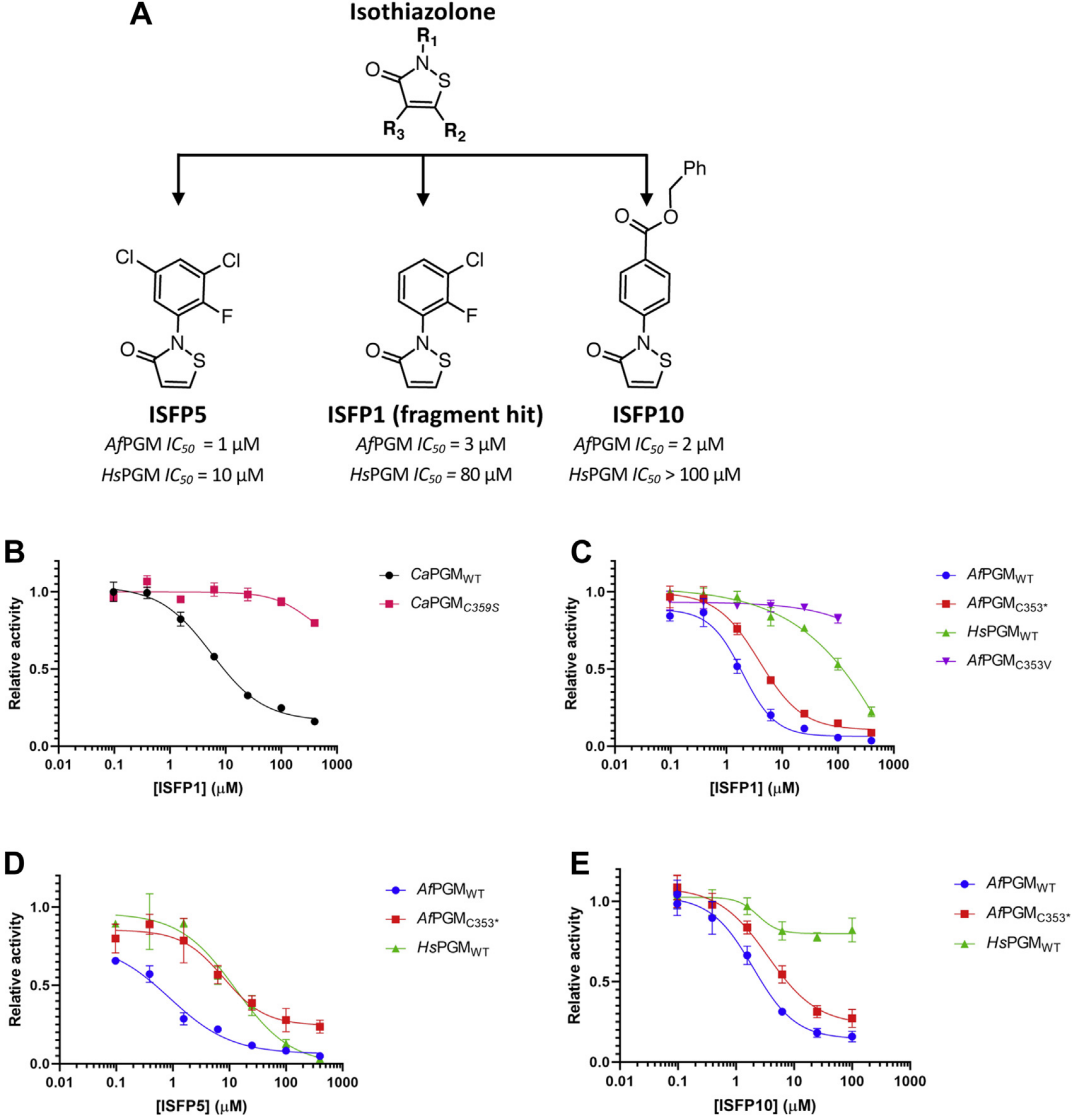
**Isothiazolones inhibit the activity of PGM**. Error bars represent SD of three determinations. Experiments were carried out without preincubating PGM (5 nM) with the inhibitor. *A*, structure of the isothiazolone scaffold and examples of synthesized isothiazolone derivatives (ISFP5 and ISFP10) that display altered selectivity against *Af*PGM and *Hs*PGM. *B*, ISFP1 (the initial fragment hit) inhibits the activity of WT *Ca*PGM (*IC*_*50*_ 5 ± 1 μM). No inhibition against *Ca*PGM_C359S_ was observed. *C*, ISFP1 inhibits the activity of WT *Af*PGM (*IC*_*50*_ 3 ± 1 μM) and the *Af*PGM variant (*Af*PGM_C353∗_, *IC*_*50*_ 4 ± 1 μM) in which C353 is the sole cysteine (all other cysteines were mutated to serine). *Af*PGM_C353V_ was not inhibited by ISFP1. ISFP1 also inhibits the activity of *Hs*PGM (*IC*_*50*_ 80 ± 2 μM). *D*, ISFP5 inhibits the activity of WT *Af*PGM (*IC*_*50*_ 1 ± 1 μM), *Af*PGM_C353∗_ (*IC*_*50*_ 8 ± 1 μM) and *Hs*PGM (*IC*_*50*_ 10 ± 1 μM). *E*, ISFP10 inhibits the activity of *Af*PGM_WT_ (*IC*_*50*_ 2 ± 1 μM) and *Af*PGM_C353∗_ (*IC*_*50*_ 4 ± 1 μM), but not *Hs*PGM (*IC*_*50*_ > 100 μM). PGM, phosphoglucomutase; *Af*PGM, *Aspergillus fumigatus* PGM; PGM, phosphoglucomutase; *Ca*PGM, *Candida albicans* PGM; *Hs*PGM, *Homo sapiens* PGM; ISFP, isothiazolone fragment of PGM.

The crystal structure of *Ca*PGM-ISFP1 suggests that ISFP1 is likely to inhibit *Ca*PGM by two hypothetical mechanisms. Therefore, we firstly sought to study whether ISFP1 inhibits the activity of PGM. Enzyme assay suggests that ISFP1 exhibits an *IC*_*50*_ of 5 μM against *Ca*PGM (Fig. 4*B*). The introduction of a C359S mutant in *Ca*PGM reduces the observed inhibitory activity (*IC*_*50*_ > 400 μM) (Fig. 4*B*), in agreement with the modification of C359 in the complex structure. As C359 in *Ca*PGM corresponds to C353 in *Af*PGM, we hypothesized that targeting C353 in *Af*PGM also inhibits the enzyme activity. Enzyme assays demonstrate that ISFP1 inhibits *Af*PGM with an *IC*_*50*_ of 3 μM (Fig. 4*C*). As expected, ISFP1 does not inhibit the *Af*PGM variant carrying a C353V mutant (Fig. 4*C*), which indicates that C353 is the sole target of ISFP1 against *Af*PGM. To validate that C353 is a target of ISFP1, an *Af*PGM triple mutant was generated, in which C353 was the only cysteine retained while other cysteines were mutated to serine (*Af*PGM_C353∗_). Enzyme assays suggest that the activity of *Af*PGM_C353∗_ can be inhibited by ISFP1 with an *IC*_*50*_ value (4 μM) similar to that of the WT enzyme (3 μM) (Fig. 4*C*). These results suggest that targeting C353 indeed elicits inhibitory activity. To confirm the formation of the adduct on C353, *Af*PGM_C353∗_ was preincubated with ISFP1 in a 1:20 ratio and analyzed by intact protein electronspray ionization mass spectrometry. ISFP1 preincubation leads to an increase (232 Da) in the Mw of *Af*PGM_C353∗_ (Fig. S8*F*). The delta-mass corresponds to the Mw of ISFP1 (230 Da), indicating a single adduct formed on C353 in *Af*PGM_C353∗_ (Fig. S8*F*).

Having demonstrated that ISFP1 inhibits *Af*PGM by modifying C353, we sought to understand the inhibitory mechanism of ISFP1. As previously described, two speculated mechanisms, perturbing substrate binding or phosphoryl transfer, have been proposed based on the complex structure of *Ca*PGM-ISFP1. As binding affinity and phosphoryl transfer can be reflected by *K*_*m*_ and *k*_*cat*_, respectively, kinetic assays were carried out in the presence of ISFP1 (Fig. S11*H*). Results (Table S1) show that the *K*_m_ value of *Af*PGM remains at the same level with the presence of ISFP1 while the *k*_*cat*_ value decreases in 10-fold with 20 μM ISFP1. These results suggest that modification of C353 does not change the affinity toward substrate but decreases the substrate turnover, in agreement with the hypothesis of modulated domain I motion, as described previously. As such, the inhibitory mechanism is likely to be a consequence of slowing the rate of phosphoryl transfer. Taken together, these results suggest that ISFP1 inhibits *Af*PGM activity through covalent modification of C353.

### Exploration of the ISFP1 fragment scaffold modulates selectivity over *Hs*PGM

The absence of C353 in *Hs*PGM implies the potential for selectivity of ISFP1 toward *Af*PGM over *Hs*PGM. To validate any selectivity in observed inhibition, inhibitory activity of ISFP1 against *Hs*PGM was determined using a G6PDH-coupled PGM activity assay. The additional complexities of the G6PDH-coupled assay precluded its use as a viable approach for determining accurate parameters of covalent engagement (*e.g. k*_inact_/*K*_I_) in assessing isothiazolone derivatives against PGM enzymes. As such, time-dependent *IC*_*50*_ experiments were carried out as an expedient alternative.

ISFP1 inhibited *Hs*PGM (*IC*_*50*_ 80 μM) with approximately 20-fold lower potency than that against *Af*PGM (*IC*_*50*_ 3 μM) (Fig. 4*C*). Furthermore, ISFP1 did not significantly display a shift in *IC*_*50*_ values against *Af*PGM over a 15-min preincubation period (Fig. S11*C*), whereas *IC*_*50*_ values against *Hs*PGM decreased from 100 μM to 7.5 μM after preincubating *Hs*PGM and ISFP1 for 15 min (Fig. S11*E*). These results suggest that although a likely pan-reactive covalent moiety (101, 112, 113), ISFP1 preferentially inhibits *Af*PGM over *Hs*PGM under the experimental conditions tested, in agreement with the fact that the theoretical p*K*_a_ of C353 in *Af*PGM (p*K*_a_ 11) is lower than that of cysteines in *Hs*PGM (p*K*_a_ >12) (Table S2). To dissect contributions of the ISFP scaffold to potency and assess any potential for selectivity over *Hs*PGM, inhibitory activities of 29 isothiazolone derivatives were measured (Table S3). Results demonstrated that conjugation of the isothiazolone amido nitrogen to an unsaturated sp^3^ carbon does not inhibit enzymatic activity of the tested *Af*PGMs (WT and *Af*PGM_C353∗_) and *Hs*PGM, even when conjugated to a sterically similar aryl substituent, reminiscent of the originally identified ISFP1 scaffold (Table S3, *e.g.*, ISFP26). On the contrary, aryl substituents on the isothiazolone nitrogen lead to inhibitory activity against *Af*PGMs (WT and *Af*PGM_C353∗_) (Table S3). As can be readily hypothesized, the inhibitory activity of the isothiazolone fragments can be enhanced generally across both *Af*PGM, *Ca*PGM, and *Hs*PGM through the introduction electron withdrawing functionality on the aryl ring attached to the isothiazolone amido nitrogen, effectively rendering the isothiazolone N-S more readily cleavable by disulfide bond formation. For example, the introduction of halogenated substituents as exemplified by ISFP5 (Fig. 4*A*) demonstrate lower micromolar *IC*_*50*_ values against *Af*PGM (*IC*_*50*_ 1 μM, Table S3), *Af*PGM_C353∗_ (*IC*_*50*_ 8 μM, Table S3), and *Hs*PGM (*IC*_*50*_ 10 μM, Table S3).

Interestingly, para-substituted aryl groups on the isothiazolone nitrogen demonstrate that fungal PGM selectivity is moderately enhanced, with *IC*_*50*_ values against *Hs*PGM approximately five- to 50-fold higher (no preincubation) than the fungal orthologs tested. For example, with the absence of preincubation, ISFP10 exhibits low micromolar *IC*_*50*_ values against fungal WT *Af*PGM (2 μM, Fig. 4*E*) and *Af*PGM_C353∗_ (4 μM, Fig. 4*E*), whereas *IC*_*50*_ values against *Hs*PGM are approximately 50- and 25-fold higher, respectively (*IC*_*50*_ *>* 100 μM, Fig. 4*E*). If enzymes were preincubated with ISFP10 for 15 min, ISFP10 shows a shift of *IC*_*50*_ decreasing from over 100 μM to 6 μM against *Hs*PGM (Fig. S11*F*), whereas no shift in *IC*_*50*_ against *Af*PGM was observed (Fig. S11*D*). These results suggest that ISFP10 shows stronger inhibitory activity against *Af*PGM. Taken together, the ISFP1 fragment hit preferentially inhibits *Af*PGM over *Hs*PGM and exploration of the ISFP1 scaffold demonstrated that modulation of isothiazolone scaffold can modulate selectivity against fungal PGMs compared to the human ortholog.

## Discussion

Mortality rates of invasive aspergillosis remain around 50% despite the contribution of medical comorbidities (1). Only limited numbers of compound classes are in the pipeline of antifungal treatment with multiple issues including drug resistance, toxicity, and undesirable drug–drug interactions (12). In recent decades, pharmaceutical companies have carried out research for novel antifungal agents with modest progression (16, 114), which is partially due to the lack of genetically and chemically well-characterized targets (20). Here, we hypothesized that PGM is a possible antifungal target against *A. fumiga*tus.

To date, discretely targeting PGM in *A. fumigatus* as a means of eliciting antifungal activity has not been reported. Although PGM has long been considered as a target against pathogenic bacteria, it is unknown whether PGM extends to pathogenic fungi (91, 115, 116, 117). This is likely due to conflicting studies regarding essentiality of PGM function in fungi. Early studies in *Saccharomyces cerevisiae* have demonstrated that the function of PGM can be restored by the overexpression of *N*-acetylphosphoglucosamine mutase, another phosphohexomutase that also exhibits PGM activity (118). As a consequence, deletion of PGM does not affect the growth of *S. cerevisiae* (119). Recently, however, a study in *G. lucidum* (which contains two PGM isozymes) has demonstrated that knockdown of one PGM leads to defective growth and an impaired cell wall, suggesting that the *pgm* gene is essential for the viability of *G. lucidum* (64). Using a promoter replacement approach, here, we have genetically demonstrated that the *pgm* gene is also essential for *A. fumigatus* growth. To our knowledge, this is the first genetic validation of *pgm* as a potential antifungal target against *A. fumigatus*.

Although we have demonstrated that the *pgm* gene is essential, it is still unknown whether this essentiality is due to a nonenzymatic or enzymatic function of the PGM protein. One strategy to resolve this issue would be to identify small molecule inhibitors that phenocopy the PGM knockdown by inhibiting the activity of PGM, that is, chemical validation of *Af*PGM using small molecule inhibitors. The lack of a high-resolution structure hampers rationally designing inhibitors against *Af*PGM. Therefore, to help resolve this issue, we solved the first structure of *Af*PGM by X-ray crystallography. The structure of *Af*PGM is similar to that of the human ortholog. Unexpectedly, the *Af*PGM–Mg^2+^-Glc-1,6-2P complex has been trapped, revealing a snapshot of the reaction prior to nucleophilic attack between the C-6 phosphoryl group of the intermediate and the hydroxyl group of S114. Unlike previously reported PGM–Glc-1,6-2P complexes that were obtained by soaking Glc-1,6-2P into PGM crystals (77, 120), the complex of *Af*PGM–Mg^2+^-Glc-1,6-2P was obtained by soaking the substrate (Glc-6P) into *Af*PGM crystals, with subsequent conversion of this substrate to Glc-1,6-2P *in crystallo*. Although the identity of Glc-1,6-2P as a reaction intermediate is supported by biochemical assays and isotopic labeling (78, 81, 121, 122, 123, 124), this is the first direct structural evidence supporting formation of this intermediate by enzymes from the α-phosphohexomutase family. Furthermore, the structure of *Af*PGM-Mg^2+^-Glc-1,6-2P exhibits a conformation closed to the transition state of phosphoryl transfer. In contrast to previous studies that solved phosphohexomutase-Glc-1,6-2P structures using catalytic serine mutants (77, 125), the structure of *Af*PGM-Mg^2+^-Glc-1,6-2P was solved using the native protein. Therefore, the structure of *Af*PGM-Mg^2+^-Glc-1,6-2P serves as the first structural evidence showing the near-transition state conformation of phosphoryl transfer by enzymes from α-phosphohexomutase family. Interestingly, our work is similar to previous studies that obtain a reaction intermediate (β-Glc-1,6-2P) *in crystallo* by β-PGM, which specifically recognizes the β-configuration of the sugar anomeric centre and belongs to a distinct enzyme family (90, 126). Instead of using serine as the catalytic residue, β-PGM uses an aspartic acid to facilitate phosphoryl transfer (90). The 1-phosphate group of β-Glc-1,6-2P is adjacent to the catalytic aspartic acid, exhibiting a trigonal bipyramid conformation corresponding to the transition state of phosphoryl transfer (90). In addition to the work on β-PGM, our work serves as another paradigm that crystallography can trap conformations that closely mimic transition states of enzymatic reactions.

By superimposing the *Af*PGM structure onto that of *Hs*PGM, we speculate that the motion of domain I abrogates enzyme activity by destabilizing the transition state. Although previous research has demonstrated that domain motion is essential for the activity of PGM, most studies focused on the function of domain IV (77, 82, 83). Few studies have addressed whether the motion of domain I modulates PGM activity. As such, our work provides a new insight of the function of domain motion in modulating the PGM activity. Moreover, we observed that the substrate-recognition mode is conserved in *Af*PGM and *Hs*PGM. This conservation of substrate-recognition mode suggests that mechanism-inspired inhibitors are likely to inhibit both *Af*PGM and *Hs*PGM. As missense mutations in *Hs*PGM cause congenital glycosylation diseases, inhibition of *Hs*PGM is likely to be toxic for humans (71). Taken together, the structure of *Af*PGM further suggests that mechanism-inspired inhibitors are not suitable tools for chemical validation studies of *Af*PGM.

To circumvent drawbacks of mechanism-inspired inhibitors, initially, we sought to identify inhibitors bound to secondary pockets formed by residues absent from *Hs*PGM and located away from the active site of *Af*PGM. In the recent decade, great efforts have been made to identify secondary pockets in PGM. Using computational approaches, several potential allosteric pockets have been predicted on the PGM protein (83, 127). However, to date, no PGM allosteric inhibitor has been reported. Alternatively, we utilized a fragment-based approach to identify small molecule binders that reversibly bound to secondary pockets on the *Af*PGM protein, with the plan to develop such binders into potent inhibitors using conventional structural-based approaches. Unexpectedly, a thiol reactive fragment (ISFP1) of *Af*PGM was instead identified from fragment screening. ISFP1 contains an isothiazolone ring that is known to react with cysteine thiols as has been demonstrated for several proteins (Table S4) involved in a range of biological processes (101). As a consequence, isothiazolones are biocides against several classes of organisms (128, 129, 130). For instance, isothiazolones inhibit the growth of *Aspergillus niger* and *C. albicans* with MIC ranging from <0.01 to 100 mg/l (131, 132), although the mechanism of lethality is unknown. Similarly, while we have observed in preliminary experiments that ISFP1 inhibits the growth of *A. fumigatus* with an MIC of 10 mg/l, it is not clear if this is (solely) through inhibition of *Af*PGM. Due to their biocidal activity, isothiazolones have long been utilized as industrial biocides for cosmetic products (128, 133, 134). Recently, several studies have raised concerns about the safety of isothiazolones. For instance, methylchloroisothiazolone and methylisothiazolone cause allergic contact dermatitis (113, 133, 135). Typically, due to their perceived nonspecific thiol reactivity, isothiazolones are widely categorized as pan-assay interference compounds that are frequently identified as hits in high-throughput screening campaigns but are often difficult to further develop into lead compounds (136, 137). Therefore, although isothiazolones inhibit the activity of several enzymes (138, 139, 140, 141), isothiazolones are not widely considered as promising hits for follow up studies unless a structure-activity relationship is clear (136, 142, 143). However, a study in *Trypanosoma cruzi* has shown that a structural analog (without halogen substitution) of ISFP1 modifies a cysteine in spermidine synthase and elicits an allosteric effect, through which the enzymatic activity is inhibited (144). This study demonstrated the usefulness of a crystallographic approach to unravel the mode of action of isothiazolones. Further studies have shown that although harboring an isothiazolone moiety, derivatives of benzoisothiazolone preferentially inhibit phosphomannose isomerase over phosphomannomutase, exhibiting acceptable profiles of absorption, distribution, metabolism, and excretion (ADME) in mice (143). Similar phenomena have also been reported in a study showing that benzoisothiazolones selectively inhibit orphan phosphatase over phosphomannomutase and display acceptable ADME parameters (142). Furthermore, ebselen (a sulfur to selenium substitution in the benzoisothiazolone ring) is a low-toxicity drug for the treatment of several diseases such as stroke and hearing loss (145, 146, 147). Recently, ebselen has been considered as a therapeutic candidate against COVID-19 (148, 149, 150). In a murine model, ebselen prevents the pathogenesis of aspergillosis with comparable efficacy as voriconazole (151). The safety of benzoisothiazolones and ebselen suggests that the toxicity issues of isothiazolones are not universal (145, 152, 153). As the current aim of this research is to develop tools for chemical validation of antifungal targets, we decided to further study the interaction between ISFP1 and *Af*PGM despite previous concerns with the development and clinical use of isothiazolones. From an academic perspective, any insights garnered from isothiazolone tool compounds may inform future screening approaches with more focussed and reactivity-tempered cysteine targeting compound libraries, as is currently popular within the fragment screening field (154).

Here, we have demonstrated that ISFP1 can inhibit *Af*PGM activity (*IC*_*50*_ 3 μM) through modification of C353, which is conserved in PGMs across several fungal species but absent in the human ortholog. Through the *Ca*PGM–ISFP1 complex structure, we have demonstrated that modification of C359 (corresponding to C353 in *Af*PGM) places the adduct proximal (5 Å) to the carboxyl group of the sugar recognition glutamate (E372 in *Ca*PGM; E366 in *Af*PGM) *via* a conformational change of W355 (corresponding to W349 in *Af*PGM). The structure of *Af*PGM–Mg^2+^-Glc-1,6-2P complex reveals that the carboxyl group of E366 (E372 in *Ca*PGM) recognizes the sugar ring *via* 3-OH and 4-OH and E372Q (*Ca*PGM) and E366Q (*Af*PGM) mutations lead to inactivation of the enzyme. This suggests that electrostatic perturbation of the sugar recognition glutamate, by extending the adduct scaffold, may be an avenue to improve inhibitory potency of ISFP1 derivatives. Moreover, the induced pocket harbors F362 (F356 in *Af*PGM) corresponding to M366 in *Hs*PGM, which suggests that it may be possible, by growing the adduct scaffold, to enhance the selectivity through aromatic interactions such as π–π stacking.

In the structure of the *Ca*PGM–ISFP1 complex, the side chain of W355 flips to the interface between domains I and III. Our study suggests that the side chain of W355 may hinder the motion of domain I and in turn elicit inhibitory activity. As such, our work implies that modification of C359 may elicit an allosteric effect that has not yet been reported in the phosphohexomutase family.

Taken together, C353 of *Af*PGM is a handle with which to further develop covalent tool compounds against *Af*PGM and more broadly, a potential new avenue to inhibit enzymes of the phosphohexomutase family in addition to mechanism-inspired inhibitors. Moreover, C353 is conserved in PGM orthologs from other pathogenic fungi, suggesting that a covalent modification strategy may lead to compounds with a broad inhibitory spectrum.

The absence of C353 in *Hs*PGM reveals the possibility of enhancing the selectivity of *Af*PGM inhibitors over *Hs*PGM. Indeed, ISFP1 exhibits higher inhibitory activity against *Af*PGM over *Hs*PGM. However, *Hs*PGM still displays an inhibitory profile after ISFP1 preincubation for 15 min, presumably through reaction with other cysteines, likely negating the use of the isothiazolone scaffold beyond an investigative tool. We observed that the *Hs*PGM protein precipitated when incubated with ISFP1, which did not occur with *Af*PGM and *Ca*PGM. There are five cysteines in *Hs*PGM and four of them are exposed on the protein surface (71). Pan-cysteine modification of *Hs*PGM by ISFP1 could destabilize *Hs*PGM and lead to protein precipitation. Furthermore, although ISFP1 inhibits the activity of *Af*PGM, enzyme activity is rescued by the addition of DTT (Fig. S11*G*). On the contrary, the enzyme activity of *Hs*PGM could not be rescued by DTT treatment (Fig. S11*G*). As such, ISFP1 permanently inactivates *Hs*PGM, in agreement with the precipitation observed during the preincubation with ISFP1. Taken together, these results imply that ISFP1 inactivates *Hs*PGM through promiscuous thiol reactivity.

The reactivity of the isothiazolone moiety toward the cysteine thiol functionality of proteins is effected by isothiazolone chemical substitution, as has been demonstrated in the literature (101, 112) and in agreement with the fact that alteration of substituents on isothiazolones changes the observed rate constants (*k*_obs_) toward 2-methyl-2-propanethiol in model systems (112). Moreover, the reaction of isothiazolones can also be affected by the differential reactivity of the thiol groups of cysteine side chains in proteins. This is likely to be affected by their p*K*_a_ values, which may, in turn, be affected by the microenvironment around the thiol groups (155, 156). Our results show that among the limited panel of isothiazolone derivatives tested, para-aryl substituents (*e.g.*, ISFP10) on the amido nitrogen of the isothiazolone ring conservatively enhance selectivity toward *Af*PGM compared to *Hs*PGM. This does suggest that selectivity can be modulated to some degree by altering substitution patterns of the isothiazolone scaffold. Although the selectivity of isothiazolones is tuneable, we do not consider isothiazolones as overly developable scaffolds for chemical validation given their higher incidence of off-targets, in agreement with the lack of robust linear correlation between *IC*_*50*_ of isothiazolone derivatives and their MIC values against *A. fumigatus* (Fig. S12).

In summary, we have genetically validated the essentiality of PGM for the viability of *A. fumigatus*, suggesting it is an antifungal target. We have demonstrated that covalent modification of a cysteine unique to fungal PGMs (*Af*PGM C353) can elicit inhibitory activity, suggesting that it is possible to identify covalent inhibitors for chemical validation of PGM as an antifungal target against *A. fumigatus*. Even with known covalent reactive moieties like isothiazolones, moderate selectivity can be achieved, opening up the possibility of implementing broader targeted covalent screening campaigns against fungal PGMs to identify more tractable and selective covalent hit molecules (157).

## Experimental procedures

### Reagents, fungal strains and growth conditions

Glc-1P, Glc-6P dehydrogenase, and *Leuconostoc mesenteroides* (*Lm*G6PDH) were obtained from Sigma-Aldrich. Isothiazolone derivatives (ISFP1, ISFP2, ISFP3, ISFP4) were purchased from Maybridge and ISFP7 from Sigma-Aldrich. The parental strain of *A. fumigatus* KU80 *ΔpyrG*^-^ was cultivated at 37 °C on solid CM supplemented with 5 mM uridine and 5 mM uracil. Spore concentration was calculated using a hemocytometer.

### The construction of pgm conditional mutant strains

A DNA fragment (Genbank ID: AFUA_3G11830, from -109–1001 of *pgm*) was amplified from *A. fumigatus* genomic DNA using primers P1 and P2 (Table S5). The DNA fragment was cloned into the downstream region of the alcohol dehydrogenase promoter (P_*alcA*_) in plasmid pAL3 (86), which contains the P_*alcA*_ and a selective marker (*pyr-4*). The recombinant plasmid was named as pALPGMN and was transformed into protoplasts of the *A. fumigatus* KU80 *ΔpyrG*^-^ strain using the PEG-mediated approach (158). Positive transformants were identified by uridine/uracil autotrophy. Initially, the confirmation of mutant strains was carried out using PCR. Briefly, primers P3 and P4 (Table S5) were utilized to amplify the *pgm* gene (1.8 kb). Primers P5 and P6 (Table S5) were utilized to amplify the selective marker *pyr-4* (1.2 kb). Primers P7 and P8 (Table S5) were utilized to amplify the DNA fragment (2.8 kb) from P_*alcA*_ to a downstream region of the *pgm* gene. Positive strains from the PCR confirmation were further validated using Southern blot. The genomic DNA of strains was digested by *Eco*RV and separated by agarose gel electrophoresis. Samples in the agarose gel were transformed onto a nylon membrane. Probe 1 (Table S5) was utilized to hybridize the 5′ region of the *pgm* gene. Probe 2 (Table S5) was utilized to hybridize the selective marker (*pyr-4*). The visualization of bands on the nylon membrane was carried out using the DIG labeling and detection kits (Roche Applied Science).

### Phenotypic analysis for the pgm conditional mutant strain

Spores of the *pgm* conditional mutant strain and the WT strains (*A. fumigatus* KU80) were cultivated at 37 °C for 48 h on solid MM supplemented with glycerol (100 mM). Fresh spores were further cultivated at 37 °C on solid MM plates supplemented with ethanol (100 mM), glycerol (100 mM), and threonine (100 mM) respectively. In addition, fresh spores were also cultivated at 37 °C on agar plates of CM and YEPD media.

### The identification of fragment binders

Amino groups of lysine side chains in PGM were biotinylated (EZ-Link NHSPEG4-Biotin) with one-to-one stoichiometry (one adduct per protein molecule). Biotinylated protein molecules were loaded on streptavidin-immobilized sensors (Super Streptavidin Biosensor). BLI was carried out using an Octet Red 384 instrument (ForteBio). Screening cycles included the baseline phase (protein was incubated for 60 s in screening buffer [25 mM Hepes–NaOH (pH 7.5), 150 mM NaCl, 2% DMSO (v/v]), the association phase (protein was incubated for 60 s in wells containing 200 μM fragment compounds dissolved in the screening buffer), and the dissociation phase (protein was incubated for 60 s in the screening buffer without any fragment compounds). Sensors blocked by biocytin were utilized as a control. Fragment compounds were purchased from Maybridge (1000 fragment compounds). Fragment compounds whose signal values were higher than the median value plus three times robust SD were considered as hits. The confirmation of hits was carried out using dose-response assays. Briefly, a BLI assay was carried out with each hit in a series of concentrations (from 0 to 500 μM). Running cycles included the baseline phase (60 s in the screening buffer), the association phase (120 s in the screening buffer with fragment compounds), and the dissociation phase (120 s in the screening buffer without compounds).

### Recombinant protein expression and purification of PGM proteins

#### *Af*PGM

The ORF was amplified from *A. fumigatus* RNA using the Takara Primescript High fidelity RT-PCR kit using primers P13 and P14 (Table S5). The PCR product was cloned into a 6His-modified version of pGEX6P1 (GE Healthcare). The protein was expressed in *E. coli* BL21 (DE3) pLysS through the addition of 250 μM IPTG at 18 °C for 20 h. Cells were lysed using French press (at 500 psi). The insoluble fraction was also removed by centrifugation (40,000*g* for 30 min). Supernatant was collected and filtered by membrane (0.2 μm). The filtered supernatant was incubated with glutathione sepharose 4B beads (4 °C for 2 h). Then the supernatant was removed using an empty column. Beads were collected and washed with Hepes buffer (25 mM Hepes–NaOH (pH 7.5), 150 mM NaCl). Subsequently, beads were resuspended in Hepes buffer and proteins were released from the beads through incubation with PreScission protease overnight at 4 °C. Protein was then loaded onto a size exclusion column (HiLoad 26/600 Superdex 75 pg, GE Healthcare) and eluted using Hepes buffer (25 mM Hepes–NaOH (pH 7.5), 150 mM NaCl).

#### *Ca*PGM

A gBlocks Gene Fragment of the full length *Ca*PGM (NCBI ID: XP_715772.2) was synthesized by Integrated DNA Technologies. The gene of *Ca*PGM was amplified using primers P15 and P16 (Table S5) and cloned into a 6His-modified version of pGEX6P1 (GE Healthcare). The expression and purification method of *Ca*PGM adopted the same protocol as that for *Af*PGM.

#### *Hs*PGM

The gene of *Hs*PGM was amplified from human RNA using the Takara Primescript High fidelity RT-PCR kit using primers P17 and P18 (Table S5) and cloned into a 6His-modified version of pGEX6P1 (GE Healthcare). The expression and purification of *Hs*PGM adopted the same protocol as that for *Af*PGM with modification (supplemented 10% v/v glycerol when cleaving the protein from Glutathione Sepharose 4B beads).

### Determination of the crystal structure of *Af*PGM and *Ca*PGM

To solve the *Af*PGM structure, *Af*PGM (10 mg/ml) was dissolved in the protein buffer (25 mM Tris–HCl (pH 7.5), 150 mM NaCl, 0.5 mM TCEP, 10 mM Glc-6P, 5 mM MgCl_2_). Crystal trays were set up by mixing equal amounts (300 nl) of protein and mother liquor (90 mM sodium fluoride, 90 mM sodium bromide, 90 mM sodium iodide, 100 mM Tris–bicine pH 8.5, 20% v/v PEG 550 MME, 10% w/v PEG 20000) using the sitting drop approach. Crystals formed at 18 °C after 2 days and were harvested using cryo-loops. X-ray diffraction data were collected at the European Synchrotron Radiation Facility. Diffraction data were processed using autoPROC (159). The phase problem was solved by molecular replacement (molrep in CCP4) with *Hs*PGM (PDB ID 5EPC) as the search model (160).

To solve the apo *Ca*PGM structure, *Ca*PGM (10 mg/ml) was dissolved in the Hepes buffer (25 mM Hepes–NaOH (pH 7.5), 150 mM NaCl). Reservoir solution (60 μl; 200 mM lithium sulfate monohydrate, 100 mM Tris–HCl pH 8.5, 30% w/v PEG 4000) was added to reservoirs of sitting-drop crystal plates. The reservoir solution was mixed with the protein solution (300 nl each) and added to crystal plates. Crystals formed at 18 °C and harvested using cryo-loops. X-ray diffraction data were collected using an in-house diffractometer (Rigaku) and processed using iMosflm (161). The phase problem was solved by molecular replacement (molrep in CCP4) with a *Ca*PGM homology model (using *Hs*PGM (PDB ID 5EPC) as the template) as the search model (160).

To solve the structure of *Ca*PGM in complex with ISFP1, crystal plates were set up using the same protocol as described for apo *Ca*PGM. Initially, crystal plates were incubated at 18 °C until crystals formed. Then, crystal trays were transferred to 12 °C incubator. Crystals were soaked for 10 min in cryoprotectant (reservoir solution plus 10% glycerol) supplemented with ISFP1 (900 μM). X-ray diffraction data were collected at the Diamond Light Source and processed using autoPROC (159). The phase problem was solved by molecular replacement (molrep in CCP4) with the apo *Ca*PGM structure as the search model (160).

For the *Af*PGM structure, refmac5 in CCP4 was utilized for the refinement (162). The *Ca*PGM structures were refined by PHENIX (163, 164, 165). Models were built with COOT (166). Images of structures were generated using PyMOL (167).

### G6PDH coupled assay for PGM

The reaction mixture contained MOPS–NaOH (pH 7.4; 50 mM), MgSO_4_ (1.5 mM), NADP^+^ (1 mM), G6PDH (0.01 U) from *L. mesenteroides*, Glc-1P (500 μM), Glc-1,6-2P (50 μM), and enzyme (5 nM). The reaction was carried out at 25 °C for 6 min in a total volume of 100 μl. Formation of NADPH was detected in real-time (every 1.5 min), measuring fluorescence signal (excitation at 360 nm; emission at 460 nm). To measure the inhibitory activity, compounds with different concentrations (3% (v/v) DMSO) were added to the reaction system. *IC*_*50*_ values were calculated using the product (NADPH) concentration at 6 min with 500 μM Glc-1P.

### Covalent modification of protein by thiol-reactive small molecule compounds

PGM was dissolved to 20 μM in Hepes buffer (25 mM Hepes–NaOH (pH 7.5), 150 mM NaCl) and incubated with compound (protein:compound 1:20; 2% (v/v) DMSO) at 20 °C for 10 min (for *Af*PGM) or 4 °C overnight (for *Af*PGM_C353∗_). Excess compound molecules were removed using desalting column (CentriPure P2, Generon). PGM (20 μM) incubated with DMSO at the same condition was utilized as a control. The removal of isothiazolone adducts was carried out through the incubation with DTT (10 mM) at 20 °C for 10 min. DTT was removed using a desalting column (CentriPure P2, Generon). The ring-opened isothiazolone protein modification was confirmed using intact protein mass spectrometry.

### Synthesis of 2-(3,5-dichloro-2-fluorophenyl)isothiazol-3(2H)-one (ISFP5)

3,3′-Dithiodipropionic acid (1 g, 4.76 mmol) was placed under an argon atmosphere in a dry round bottom flask (sealed with a turnover septum) and was dissolved in anhydrous tetrahydrofuran (20 ml). The flask was then cooled on-ice followed by the dropwise addition of thionyl chloride (2.07 ml, 28.53 mmol) with stirring. *N*,*N*-dimethylformamide (74 μl, 0.95 mmol) was then added to the stirring solution, and the reaction was left to stir on ice for 20 min, at which point the ice was removed and the reaction was allowed to warm to room temperature. The reaction was stirred at room temperature for a further 3 h at which point the flask was purged with argon and the solvent was carefully removed *in vacuo* using a rotary evaporator (no heating). The resulting crude material was triturated twice using toluene followed by solvent removal *in vacuo* (no heating). The crude material was again dissolved in tetrahydrofuran (<10 ml) and was evaporated to dryness *in vacuo* (no heating). The crude material was subsequently used without further purification.

3,5-dichloro-2-fluoroaniline (1 g, 5.5 mmol) was dissolved in anhydrous dichloromethane (5 ml) under an argon atmosphere. Triethylamine (6.95 mmol, 969 μl) was then added to the stirring solution and cooled on-ice. Separately, 3,3′-disulfanediyldipropionyl chloride from the first reaction (344 mg, 1.39 mmol) was suspended in anhydrous dichloromethane (5 ml) and was then added slowly to the stirring 3,5-dichloro-2-fluoroaniline solution. The reaction was allowed to stir on-ice for 30 min, then warmed to room temperature, and allowed to stir overnight. The next day, a saturated aqueous solution of sodium hydrogen carbonate (approx. 40 ml) was added slowly to the crude reaction mixture and the resulting solution was extracted using dichloromethane (3 × 20 ml minimum). The organic phase was dried over sodium sulfate, filtered, and the solvent removed *in vacuo* (water bath at 35 °C). The crude material was dry loaded on silica and was purified by flash chromatography (Heptane/Ethyl acetate (2:1) → Ethyl acetate) (305 mg, 41% yield).

The product, 3,3′-disulfanediylbis(*N*-(3,5-dichloro-2-fluorophenyl)propanamide) (210 mg, 0.39 mmol) was suspended (not dissolved) in anhydrous dichloromethane under an argon atmosphere and was cooled on-ice. A solution of 1M sulfuryl chloride in dichloromethane (∼1 eq., 393 μl) was then added to the stirring flask and was stirred for 15 min. Over the course of the following 1 to 2 h, an additional 4 to 5 equivalents of sulfuryl chloride solution was added to the stirring solution until the solubility of the material in the flask improved. At this point, the reaction mixture was allowed to warm to room temperature and stirred overnight. The next day, saturated brine solution (approx. 20 ml) was added to the off-white suspension followed by extraction with ethyl acetate (3 × 20 ml minimum). The organic phase was dried over sodium sulfate, filtered, and the solvent removed *in vacuo* (water bath at 30 °C) to the point where the solid crude product became visible in the flask. At this point, the reaction mixture was resolubilized with the minimum amount of solvent and was dry loaded onto silica for flash purification (Heptane/ethyl acetate (1:1) → ethyl acetate). The flash purified material then underwent a further purification by reverse phase C18 HPLC (see supplementary information for methods) (R_f_: 0.65; heptane/ethyl acetate, 1:1) (19 mg, postlyophilisation, 18% yield).

^1^H NMR (500 MHz, CDCl_3_) δ 8.23 (d, J = 6.4 Hz, 1H), 7.46 to 7.33 (m, 2H), 6.27 (d, J = 6.4 Hz, 1H).

^13^ C NMR (126 MHz, CDCl_3_) δ 167.64, 153.98 to 151.88 (J = 254.9 Hz), 141.77, 130.76, 129.55 (m), 128.19, 125.51 (m), 123.57 (m), 113.43.

^19^ F NMR (471 MHz, CDCl_3_) δ -121.13.

HRMS: Theo. m/z 263.9453 Da; obs. m/z 263.9464 Da [M + H]. Error (4.2 ppm).

### Synthesis of benzyl 4-(3-oxoisothiazol-2(3H)-yl)benzoate (ISFP10)

4-Nitrobenzoic acid (5 g, 29.9 mmol) was dissolved in anhydrous acetonitrile. Potassium carbonate (4.96 g, 35.88 mmol) was then added, followed by the dropwise addition of benzyl bromide (3.73 ml, 31.95 mmol). The resulting mixture was then heated to 60 °C with stirring over 6 h. After cooling to room temperature, brine solution (approx. 150 ml) was added and the reaction mixture was then extracted with ethyl acetate (3 × 20 ml minimum). The organic phase was dried over sodium sulfate, filtered, and the solvent removed *in vacuo*. The crude product was then triturated with toluene to remove residual volatile components and was carried through to the next step without further purification (R_f_: 0.32; heptane/ethyl acetate, 5:1).

The crude product, benzyl 4-nitrobenzoate (6.42 g, approx. 24.95 mmol), was then dissolved in ACS grade ethanol (50 ml), followed by the addition of deionized water (16.7 ml), iron powder (4.18 g, 74.87 mmol), and ammonium chloride (4 g, 74.87 mmol). The heterogeneous reaction mixture was stirred rapidly and heated to 75 to 80 °C for 1.5 to 2 h, after which time TLC analysis revealed reaction completion (R_f_: 0.35; heptane/ethyl acetate, 1:1). The crude reaction mixture was filtered twice over Celite to remove solid material, and the resulting filtrate was evaporated to dryness *in vacuo*. The crude benzyl 4-aminobenzoate material was then dissolved in the minimum amount of warm methanol/ethyl acetate (2:1) and was passed over a silica plug. The resulting methanolic solution was then evaporated to dryness and the compound was subsequently used without further purification.

The 3,3′-disulfanediyldipropionyl chloride intermediate was then synthesized as described in the method above. Crude benzyl 4-aminobenzoate (2.86 g, 12.6 mmol) was dissolved in anhydrous dichloromethane (15 ml) under an argon atmosphere. Triethylamine (15.78 mmol, 2.2 ml) was then added to the stirring solution and cooled on-ice. Separately, 3,3′-disulfanediyldipropionyl chloride was suspended in anhydrous dichloromethane (15 ml) and was then added slowly to the stirring benzyl 4-aminobenzoate solution. The reaction was allowed to stir on-ice for 30 min and, then warmed to room temperature, and was allowed to stir overnight. The next day, a saturated aqueous solution of sodium hydrogen carbonate (approx. 100 ml) was added slowly to the crude reaction mixture and the resulting solution was extracted using dichloromethane (3 × 40 ml minimum). The organic phase was dried over sodium sulfate, filtered and the solvent removed *in vacuo* (water bath at 35 °C). The crude material began to crash out of solution on solvent removal. The solid material was collected and dried by filtration and overnight drying on a high vacuum line (R_f_: 0.40; heptane/ethyl acetate, 1:1).

The product, dibenzyl 4,4'-((3,3′-disulfanediylbis(propanoyl))bis(azanediyl))dibenzoate (240 mg, 0.382 mmol) was suspended (not dissolved) in anhydrous dichloromethane (10 ml) under an argon atmosphere and was cooled on-ice. A solution of 1M sulfuryl chloride in dichloromethane (approx. 1 eq., 0.38 ml) was then added to the stirring flask and was stirred for 15 min. Over the course of the following 1 to 2 h, an additional 4 to 5 equivalents of sulfuryl chloride solution was added to the stirring solution until the solubility of the material in the flask improved. At this point, the reaction mixture was allowed to warm to room temperature and was stirred overnight. The next day, saturated brine solution (approx. 20 ml) was added to the off-white suspension followed by extraction with ethyl acetate (3 × 20 ml minimum). The organic phase was dried over sodium sulfate, filtered, and the solvent removed *in vacuo* (water bath at 30 °C) to the point where the solid crude product became visible in the flask. At this point, the reaction mixture was resolubilized with the minimum amount of solvent and was dry loaded onto silica for flash purification (Heptane/ethyl acetate → ethyl acetate). The flash purified material then underwent a further purification by reverse phase C18 HPLC (see supplementary information for methods) (R_f_: 0.15; heptane/ethyl acetate, 1:1) (15 mg, postlyophilisation, 13% yield).

^1^H NMR (500 MHz, CDCl_3_) δ 8.08 (dd, J = 13.2, 7.4 Hz, 3H), 7.67 (d, J = 8.4 Hz, 2H), 7.38 to 7.23 (m, 5H), 7.17 (s, 1H), 6.25 (d, J = 6.4 Hz, 1H), 5.29 (s, 2H).

^13^ C NMR (126 MHz, CDCl_3_) δ 167.36, 165.51, 141.05, 139.61, 135.92, 130.99, 128.64, 128.33, 128.16, 123.11, 115.38, 66.90.

HRMS: Theo. m/z 312.0694 Da; obs. m/z 312.0721 Da [M + H]. Error (8.7 ppm).

## Data availability

Crystal structures reported in this article have been deposited in the Protein Data Bank with accession codes 7PIZ, 7PJC, and 7P5O. Other data are either shown in this article or can be provided upon request (Prof. Daan M. F. van Aalten, University of Dundee, d.m.f.vanaalten@dundee.ac.uk).

## Supporting information

This article contains supporting information (71, 95, 108, 138, 139, 140, 142, 143, 144, 167, 169, 170, 171, 172, 173, 174, 175, 176, 177, 178, 179, 180, 181, 182, 183, 184, 185, 186, 187, 188).

## Conflict of interest

The authors declare no conflict of interest.

## Supporting information

Figure_S12

NMR_data
