# Supplementary material for: Genetic validation of Aspergillus fumigatus phosphoglucomutase as a viable therapeutic target in invasive aspergillosis
Source: J Biol Chem. 2022 Apr 30;298(6):102003. doi: 10.1016/j.jbc.2022.102003 (PMC9168620; doi:10.1016/j.jbc.2022.102003)
Supplement: Supporting_information_revised2.Docx [file mmc2.docx]

**Supporting information**

**Genetic validation of *Aspergillus fumigatus* phosphoglucomutase as a viable therapeutic target in invasive aspergillosis**

**Supplemental figures**

**Figure S1: Size exclusion chromatography and kinetic analysis of *Af*PGM**

1. Elution profile of *Af*PGM from size exclusion chromatography. The main peak showed a single band (between 55 kDa and 72 kDa) in SDS-PAGE, in agreement with the M_w_ of an *Af*PGM monomer (61 kDa). The peak fraction corresponds to a M_w_ of 63 kDa, which suggests that *Af*PGM is a monomer in solution. Intact mass spectrometry indicates that the peak fraction was a mixture of phosphorylated (observed 60980 Da, theoretical 60992 Da) and dephosphorylated (observed 60898 Da, theoretical 60913 Da) *Af*PGM.
2. *Af*PGM activity for Glc-1P was determined using a G6PDH coupled assay. The determination of kinetic parameters (*K*_m_, *k*_cat_) was carried out through application of Michaelis–Menten kinetic analysis (Prism, GraphPad). Error bars represent standard deviation with three determinations.
3. MS/MS analysis demonstrates that the catalytic serine (TASHNP) is phosphorylated. Briefly, the protein sample was trypsinised and analysed using a Q-Exactive Plus (ThermoFisher). Peptides were fragmentated through high-energy collisional dissociation.

**Figure S2: Construction of the *pgm* conditional mutant strains in *A. fumigatus.***

1. The strategy for constructing *pgm* conditional mutant strains. The native promoter of *pgm* was replaced by a tightly regulated promoter P*_alcA_* (alcohol dehydrogenase promoter from *A. nidulans*).
2. PCR confirmation of *pgm* conditional mutant strains. The *pgm* gene was amplified from both WT and mutant strains. The selective marker *pyr-4* was only amplified from the conditional mutant. The region from P*_alcA_* to a region 500 bp downstream *pgm* was only amplified from the conditional mutant.
3. Southern blot confirmation of *pgm* conditional mutant strains. Probe 1 and probe 2 were utilized to hybridize the *pgmN* and the selective marker *pyr-4,* respectively. Potential mutant No. 25 showed correct profiles to both probes and was considered to be the correct conditional mutant strain.

**Figure S3: Surface representation of structures of PGMs.** The structure of *Af*PGM is shown in (A). The apo structure of *Hs*PGM (PDB code 5EPC) (1) is shown in (B). The structure of *Hs*PGM in complex with Glc-6P (PDB code 6UIQ) (2) is shown in C. The structure of *Hs*PGM in complex with Glc-1P (PDB code 6SNO) (3) is shown in (D). Domain I is shown in cyan. Domain II is shown in yellow. Domain III is shown in grey. Domain IV is shown in magenta.

**Figure S4: Hypothesized functions of T18 and R22 in the catalytic mechanism of *Af*PGM.**

1. Superposition of the structure of *Af*PGM onto that of *Hs*PGM (PDB code 6SNO). Grey indicates domain I of *Af*PGM, and the rest of *Af*PGM is shown in light blue. Magenta represents domain I of *Hs*PGM. Cyan indicates the rest of *Hs*PGM. The close-up panel is shown in stereoscopic view. Orange dash lines represent hydrogen bonds.
2. Scheme shows proposed functions of T18 and R22 in the mechanism of *Af*PGM. Dash arrows represent postulated direction of protein domain movement.

**Figure S5: The active site of *Af*PGM in complex with Glc-1,6-2P and Mg^2+^.** *Af*PGM is shown in grey. The Mg^2+^ ion is shown as a green sphere. Orange sticks represent Glc-1,6-2P. Magenta mesh represents 2F_o_-F_c_ map contoured at 1σ. Images are shown in stereoscopic view.

**Figure S6: Binding of ISFP1 to *Af*PGM using biolayer interferometry.**

1. Biolayer interferometry showed that ISFP1 can bind to *Af*PGM. Values of concentration range from 0 to 500 µM.

# **Figure S7: Sequence alignment of PGM from various organisms.**

Red frames and triangles indicate active site motifs. Cysteines in *Af*PGM are shown in yellow. The blue circle indicates C353 in *Af*PGM. Brown stars indicate residues forming the induced pocket. Red indicates W355 in *Ca*PGM. Magenta represents F362 in *Ca*PGM. Sequences were obtained from the Genbank, including *A. fumigatus* (XP_754438.1), *C. albicans* (XP_715772.2), *H. capsulatum* (EDN05153.1), *P. jirovecii* (KTW30985.1), *S. chartarum* (KFA71509.1), *C. neoformans* (OXH63671.1) and *H. sapiens* (NP_002624.2).

# **Figure S8: Deconvoluted mass spectrometry data from intact protein LC-MS analyses of *Af*PGM and *Af*PGM_C353_.**

1. Intact mass spectrometry of *Af*PGM. Two protein species are observed, corresponding to the phosphorylated (observed 60983 Da, theoretical 60992 Da) and the dephosphorylated (observed 60908 Da, theoretical 60913 Da) forms, respectively.
2. Intact mass spectrometry of *Af*PGM after incubation with fragment hit, ISFP1. The molecular weight (M_w_) difference between the main species (61215 Da) and phosphorylated *Af*PGM (60983 Da) is 232 Da, in agreement with a single ISFP1 adduct (230 Da). The second most abundant species has an M_w_ value of 61450 Da, which exhibits a 467 Da difference to the M_w_ of phosphorylated *Af*PGM (60983 Da), in agreement with a double ISFP1 adduct (230 Da).
3. Intact mass spectrometry of natural *Af*PGM after incubation with dithiothreitol (DTT). The M_w_ of the main species is 60981 Da corresponding to phosphorylated *Af*PGM (60983 Da). The second most abundant species shows an M_w_ of 60906 corresponding to dephosphorylated *Af*PGM (60908 Da).
4. Intact mass spectrometry of ISFP1-modified *Af*PGM incubated with DTT. The M_w_ of the main species is 60986 Da corresponding to phosphorylated *Af*PGM (60983 Da).
5. Intact mass spectrometry of *Af*PGM_C353*_, which reveals two protein species corresponding to the phosphorylated (60928 Da) and dephosphorylated (60851 Da) forms of protein.
6. Intact mass spectrometry of *Af*PGM_C353*_ after incubation with ISFP1. The molecular weight (M_w_) of the main species (61160 Da) shows a 232 Da difference to that of the phosphorylated (60928 Da) protein, in agreement with the modification by ISFP1 (230 Da). The M_w_ of the second most abundant species (61081 Da) exhibits a 230 Da difference to the dephosphorylated protein (60851 Da), in agreement with the modification by ISFP1 (230 Da).

**Figure S9: Crystal structures of *Af*PGM and *Ca*PGM**

1. C353 is located in a surface cleft within *Af*PGM. Domains I–IV are shown in cyan, yellow, grey and blue respectively. Residues absent from *Hs*PGM are shown in red. Close-up panel shows a stereoscopic view of the cleft.
2. Superposition of *Ca*PGM-ISFP1 complex (grey) and native *Ca*PGM (magenta). Images are shown as stereoscopic view.

**Figure S10: Domains I&III interface in the structure of *Ca*PGM.**

1. The side chain of W355 (green sticks) flips to the domain I&III interface in the *Ca*PGM-ISFP1 complex structure. The close-up panel is shown in stereoscopic view. The right panel (stereoscopic view) shows interactions between W355 and residues at the domain I&III interface. Cyan ribbon represents domain I. Grey ribbon indicates domain III. Light blue ribbon represents the structure of *Af*PGM superimposed onto that of *Ca*PGM-ISFP1 complex. Orange dash lines indicate hydrogen bonds.
2. Homology model of the “closed” *Ca*PGM (QMEANDisCo Global 0.73 ± 0.05) (4). The close-up panel is shown in stereoscopic view. The right panel (stereoscopic view) shows superposition of the structure of *Ca*PGM-ISFP1 (only shows W355) onto the homology model. Colour information is the same as described in (a).
3. Prediction of an allosteric pocket on the *Ca*PGM protein. The prediction was carried out using AlloSitePro (http://mdl.shsmu.edu.cn/AST/). The predicted allosteric pocket is shown as white spheres. The close-up panel is shown in stereoscopic view. Colour information of the protein is the same as that in (a).

**Figure S11: Representative enzyme activity assays for PGMs. Error bars represent standard deviation of three determinations.** Panel A and B show the time course of PGMs and PGM mutants in which sugar recognition glutamates are mutated to glutamine. Panel C to F show the shift of *IC_50_* values over pre-incubation (15 min) with ISFP1/ISFP10. Panel G shows the effect of DTT on the inhibitory activity of ISFP1. Briefly, ISFP1 (50 µM) was pre-incubated with 5 nM of *Af*PGM or *Hs*PGM for 15 min. Values in parentheses indicate the time at which DTT (1 mM) was added. Panel H shows the determination of *Af*PGM (2 nM) enzyme kinetics in the presence of varied ISFP1 and substrate (G1P) concentrations.

**Figure S12: Limited correlation between minimal inhibitory concentrations (MIC) and *IC_50_* values of isothiazolone derivatives against *Af*PGM.** The X-axis shows *IC_50_* values against *Af*PGM, and Y-axis shows MIC values against *A. fumigatus* (ATCC204305). The R^2^ value of linear correlation is 0.15.

**Supplemental tables**

**Table S1. Kinetic parameters of *Af*PGM determined in the presence of ISFP1.**

# **Table S2 Calculated p*K*_a_ values of cysteines in *Af*PGM and *Hs*PGM.** Calculation was carried out using H++ (<http://biophysics.cs.vt.edu>) (5–7).

**Table S3: *IC_50_* of derivatives against *Af*PGM, *Af*PGMC353 and *Hs*PGM^a^**

^a^ *IC_50_* values of derivatives (Fig. 4A) have been determined using a G6PDH-coupled assay without pre-incubation of enzymes (5 nM) and compounds. The core structure is shown in Fig. 4A. ND indicates no data.

^b^ The highest concentration at which the compound can solubilize.

**Table S4 Potential targets that are likely to be hit by isothiazolones**

**Table S5: Primers used in this research.**

**Movie S1. Pymol movie demonstrating conformational changes of *Af*PGM and the hypothesized impact of C353 modification by ISFP1 upon domain motion (illustrated using the *Ca*PGM-ISFP1 complex).** The structure of *Af*PGM-Mg^2+^-Glc-1,6-2P represents the “closed” conformation. The “open” conformation was obtained by homology modelling (SWISS-MODEL) using the structure of apo *Hs*PGM (PDB code 5EPC) as the template. Domains are coloured as cyan (I), yellow (II), grey (III) and magenta (IV) respectively. Red indicates the secondary structure component harbouring R22 and T18. W355 in *Ca*PGM (green sticks) is placed by superimposing the structure of *Ca*PGM-ISFP1 onto that of the “open” *Af*PGM. Orange sticks represent Glc-1,6-2P. The video was made using PyMOL (24).

**General and analytical procedures**

Chemicals were purchased from Merck-Sigma, TCI UK, Fluorochem, Alfa Aesar, Carbosynth and Maybridge and were used without further purification. All laboratory-reagent grade, analytical grade and anhydrous solvents were purchased from Merck-Sigma, Fisher Scientific and Acros Organics. Air- and moisture-sensitive reactions were carried out under an inert atmosphere of argon in dried glassware. Thin-layer chromatography (TLC) was performed on precoated TLC plates (POLYGRAM® SIL G/UV254, Macherey-Nagel). Developed plates were dried and analysed under a UV lamp (UV254/365 nm). Ultra-pure water was obtained from a Millipore milliQ (MQ) Advantage system.

Small molecule normal-phase FLASH chromatography and preparative small molecule C18 reversed-phase high-performance liquid chromatography (HPLC) were carried out using a Buchi PrepChrom C700 chromatography system. Normal-phase FLASH chromatography utilised standard silica dry loading procedures and column separation using RediSep pre-packed silica columns (Teledyne Isco) in conjunction with common combinations of organic solvents mobile phases (methanol, ethyl acetate, ethyl acetate/methanol, ethyl acetate/n-heptane). Preparative small molecule HPLC utilised a Buchi C18 column and a Phenomenex C18 guard column with acetonitrile (ACN) and MQ water (supplemented with 0.1% formic acid) as mobile phase. HPLC purified fractions were dried using a rotary evaporator or were lyophilised to dryness *in vacuo*.

Small molecule liquid chromatography mass spectrometry (LC-MS) was carried out using electrospray ionization (ESI) on an Agilent Technologies 1200 single quadrupole LC-MS system fitted with a Max-Light Cartridge flow cell coupled to a 6130 Quadrupole spectrometer. An Agilent ZORBAX Eclipse Plus C18, 4.6 x 100 mm, 3.5 μm column was used for separations. Variable wavelengths were used and MS acquisitions were carried out in positive and negative ion modes. Small molecule High-resolution mass spectrometry (HRMS) was carried out using ESI on a SCIEX Xevo-QTOF (Q-Time of Flight) MS instrument coupled to a Waters Acquity LC (liquid chromatography) system. HPLC-grade ACN and water were used as the mobile phase systems for LC separations on both systems, supplemented with trifluoroacetic acid (TFA) (Agilent system) or formic acid (SCIEX system).

Intact protein mass was carried out using the Agilent instrument using an Agilent ZORBAX 300SB-C3 5um, 2.1 x 150mm column, unless otherwise stated. Protein MS acquisition was carried out in positive ion mode and total protein masses were calculated by deconvolution within the MS Chemstation software (Agilent Technologies). The LC-MS solvent system consisted of 0.05 % TFA in water as buffer A, and 0.04 % TFA acid in ACN as buffer B. Protein UV absorbance was monitored at 214 nm, 254 nm and 280 nm.

NMR spectra were recorded on a Bruker Avance II 500 MHz spectrometer or a Bruker Ascend 400 MHz spectrometer at room temperature. Chemical shifts (δ) are expressed in ppm recorded using the residual solvent as the internal reference in all cases. The following abbreviations are used to indicate the signal multiplicity: s (singlet), bs (broad singlet), d (doublet), t (triplet), q (quartet), dd (doublet of doublets), m (multiplet).

**Synthetic procedures**

The synthesis of isothiazolone fragments highlighted in Table S2 was achieved using reported literature methods unless commercially sourced. NMR and HRMS analytical data have been provided for final target compounds.

**Synthetic procedure 1**

In brief, generation of the isothiazolone scaffold was achieved using widely reported literature procedures (10, 25, 26) by generating 3,3'-dithiodipropionic acid chloride from 3,3'-dithiodipropionic acid (1 eq.) through reaction with thionyl chloride (6 eq.) and N,N-dimethylformamide (0.2 eq.) in anhydrous tetrahydrofuran/dichloromethane. The resulting acid chloride intermediate (1 eq.) was then reacted with a suitable amino- or aryl anilino-containing functionality (3-4 eq.), forming the desired amide-conjugated disulfide precursor. Cyclisation of the amide-conjugated disulfide precursor to generate the 5-membered isothiazolone ring was achieved using sulfuryl chloride (1-3.5 eq.) in anhydrous dichloromethane, as previously reported. Where possible, the required aryl anilino-containing functionality was commercially sourced to expedite synthesis. However, if not commercially practical or possible, nitro-group containing synthetic precursors were used as anilino-synthons for use in amide forming reactions, using standard nitro-group reduction conditions to generate the free anilino-group (27, 28). Crude reaction products were purified by standard FLASH chromatography and preparative reversed phase HPLC methods using conditions described in the general and analytical procedures section.

**Synthetic procedure 2**

Appropriate aryl anilino starting material (1 eq.) was conjugated to methacrylic acid using standard amide coupling chemistry using N,N,N′,N′-Tetramethyl-O-(1H-benzotriazol-1-yl)uronium hexafluorophosphate (HBTU) (1.2 eq.) in N,N-dimethylformamide. The resulting amido-conjugate product (1 eq.) then underwent palladium catalysed C-H bond activation in the presence of palladium dichloride (20 mol%) and thiocyanate donor, 2-thiocyanatoisoindoline-1,3-dione, as reported in the literature (29). Crude reaction products were purified by standard FLASH chromatography and preparative reversed phase HPLC methods using conditions described in the general and analytical procedures section.

**Synthetic procedure 3**

Appropriate aryl anilino starting material (1 eq.) was conjugated to a suitably functionalised propiolic acid using standard amide coupling chemistry using N,N,N′,N′-Tetramethyl-O-(1H-benzotriazol-1-yl)uronium hexafluorophosphate (HBTU) (1.2 eq.) in N,N-dimethylformamide. The resulting amido-conjugate product (1 eq.) then underwent copper-catalysed thioannulation in the presence of sodium sulfide (2.5 eq.) and iodine (1 eq.), as reported in the literature (30). Crude reaction products were purified by standard FLASH chromatography and preparative reversed phase HPLC methods using conditions described in the general and analytical procedures section.

**Synthesis of isothiazolone derivatives**

**ISFP8**

*tert*-butyl 4-(3-oxoisothiazol-2(3H)-yl)benzoate

Method: Synthetic procedure 1

^1^H NMR (500 MHz, CDCl_3_) δ 8.06 (d, J = 6.3 Hz, 1H), 7.93 (d, J = 8.5 Hz, 2H), 7.60 (d, J = 8.7 Hz, 2H), 6.21 (d, J = 6.4 Hz, 1H).

^13^C NMR (126 MHz, CDCl_3_) δ 167.41, 164.83, 140.44, 139.56, 130.66, 130.36, 123.07, 115.36, 81.38, 28.20, 2 x 13C resonances not observed.

HRMS: Theo. m/z 278.0851 Da; obs. m/z 278.086 Da [M+H]. Error (3.2 ppm).

**ISFP11**

isopropyl 4-(3-oxoisothiazol-2(3H)-yl)benzoate

Method: Synthetic procedure 1

^1^H NMR (400 MHz, CDCl_3_) δ 8.13 (d, J = 6.4 Hz, 1H), 8.07 – 7.99 (m, 2H), 7.70 – 7.62 (m, 2H), 6.26 (d, J = 6.4 Hz, 1H), 5.17 (h, J = 6.2 Hz, 1H), 1.30 (d, J = 6.3 Hz, 6H).

^13^C NMR (101 MHz, CDCl_3_) δ 167.42, 165.17, 140.68, 139.82, 130.76, 130.61, 129.21, 123.16, 115.26, 68.70, 21.92.

HRMS: Theo. m/z 264.0694 Da; obs. m/z 264.0701 Da [M+H]. Error (2.7 ppm).

**ISFP12**

2-(3-nitrophenyl)isothiazol-3(2H)-one

Method: Synthetic procedure 1

^1^H NMR (500 MHz, MeOH-D4) δ 8.54 (d, J = 6.4 Hz, 1H), 8.47 (t, J = 2.2 Hz, 1H), 8.13 (ddd, J = 8.3, 2.2, 0.9 Hz, 1H), 7.90 (ddd, J = 8.1, 2.1, 0.9 Hz, 1H), 7.65 (t, J = 8.2 Hz, 1H), 6.25 (d, J = 6.4 Hz, 1H).

^13^C NMR (126 MHz, MeOH-D4) δ 168.71, 143.51, 137.77, 130.31, 129.90, 121.58, 119.01, 113.65.

HRMS: Theo. m/z 223.0177 Da; obs. m/z 223.0186 Da [M+H]. Error (4.0 ppm).

**ISFP13**

2-(3-chlorophenyl)isothiazol-3(2H)-one

Method: Synthetic procedure 1

^1^H NMR (500 MHz, CDCl_3_) δ 8.17 (d, J = 6.4 Hz, 1H), 7.66 (t, J = 2.1 Hz, 1H), 7.51 (ddd, J = 8.1, 2.1, 1.0 Hz, 1H), 7.37 (t, J = 8.0 Hz, 1H), 7.29 (ddd, J = 8.1, 2.0, 1.0 Hz, 1H), 6.32 (d, J = 6.4 Hz, 1H).

^13^C NMR (126 MHz, CDCl_3_) δ 167.35, 139.61, 137.80, 135.04, 130.31, 127.42, 124.56, 122.44, 115.07, 115.04.

HRMS: Theo. m/z 211.9937 Da; obs. m/z 211.9947 Da [M+H]. Error (4.7 ppm).

**ISFP14**

3-(3-oxoisothiazol-2(3H)-yl)benzonitrile

Method: Synthetic procedure 1

^1^H NMR (500 MHz, CDCl_3_) δ 8.26 (d, J = 6.4 Hz, 1H), 8.00 (t, J = 1.8 Hz, 1H), 7.89 (dt, J = 7.6, 2.1 Hz, 1H), 7.63 – 7.53 (m, 2H), 6.36 (d, J = 6.4 Hz, 1H).

^13^C NMR (126 MHz, CDCl_3_) δ 167.39, 140.24, 137.70, 130.45, 130.36, 128.29, 127.35, 117.77, 117.31, 114.95, 113.62.

HRMS: Theo. m/z 203.0279 Da; obs. m/z 203.0279 Da [M+H]. Error (0.0 ppm)

**ISFP15**

ethyl 5-(3-oxoisothiazol-2(3H)-yl)benzofuran-2-carboxylate

Method: Synthetic procedure 1

^1^H NMR (400 MHz, Acetone) δ 8.65 (d, J = 6.4 Hz, 1H), 8.05 (t, J = 1.4 Hz, 1H), 7.78 – 7.69 (m, 3H), 6.29 (d, J = 6.4 Hz, 1H), 4.44 (q, J = 7.1 Hz, 2H), 1.42 (t, J = 7.1 Hz, 3H).

^13^C NMR (126 MHz, Acetone) δ 167.19, 158.59, 153.86, 147.27, 141.53, 133.50, 127.63, 124.84, 119.21, 114.13, 113.59, 112.60, 61.19, 13.63.

HRMS: Theo. m/z 290.0487 Da; obs. m/z 290.0504 Da [M+H]. Error (5.9 ppm).

**ISFP16**

2-(2-fluorophenyl)isothiazol-3(2H)-one

Method: Synthetic procedure 1

^1^H NMR (500 MHz, CDCl_3_) δ 8.19 (d, J = 6.4 Hz, 1H), 7.43 – 7.29 (m, 2H), 7.21 – 7.11 (m, 2H), 6.28 (d, J = 6.4 Hz, 1H).

^13^C NMR (126 MHz, CDCl_3_) δ 168.15, 159.07 – 157.05 (d, J = 253.58 Hz), 141.32, 130.73 – 130.66 (d, J = 7.9 Hz), 129.94, 124.78 – 124.74 (d, J = 4.0 Hz), 123.45 – 123.34 (d, J = 12.7 Hz), 117.06 – 116.91 (d, J = 19.6 Hz), 113.67.

19F NMR (471 MHz, CDCl_3_) δ -118.67.

HRMS: Theo. m/z 196.0232 Da; obs. m/z 196.0242 Da [M+H]. Error (5.1 ppm).

**ISFP17**

2-(naphthalen-1-yl)isothiazol-3(2H)-one

Method: Synthetic procedure 1

^1^H NMR (500 MHz, Acetone) δ 8.66 (d, J = 6.3 Hz, 1H), 7.95 – 7.88 (m, 2H), 7.56 – 7.42 (m, 5H), 6.21 (d, J = 6.3 Hz, 1H).

^13^C NMR (126 MHz, Acetone) δ 205.17, 168.23, 142.77, 134.53, 133.15, 130.97, 129.55, 128.39, 127.50, 127.07, 126.64, 125.47, 123.00, 113.11.

HRMS: Theo. m/z 228.0483 Da; obs. m/z 228.0497 Da [M+H]. Error (6.1 ppm).

**ISFP18**

2-(4-(morpholine-4-carbonyl)phenyl)isothiazol-3(2H)-one

Method: Synthetic procedure 1

^1^H NMR (500 MHz, MeOD) δ 8.49 (d, J = 6.3 Hz, 1H), 7.61 (d, J = 8.6 Hz, 2H), 7.46 (d, J = 8.5 Hz, 2H), 6.22 (d, J = 6.3 Hz, 1H), 3.71 – 3.33 (bm, 8H).

^13^C NMR (126 MHz, MeOD) δ 170.03, 168.74, 143.18, 138.03, 134.23, 128.12, 124.60, 113.67, 66.37.

HRMS: Theo. m/z 291.0803 Da; obs. m/z 291.0826 Da [M+H]. Error (7.9 ppm).

**ISFP19**

3-(3-oxoisothiazol-2(3H)-yl)benzyl acetate

Method: Synthetic procedure 1

^1^H NMR (500 MHz, Acetone) δ 8.62 (d, J = 6.4 Hz, 1H), 7.72 (m, 1H), 7.64 (dd, J = 8.0, 1.8 Hz, 1H), 7.49 (t, J = 7.9 Hz, 1H), 7.36 (d, J = 7.6 Hz, 1H), 6.27 (d, J = 6.4 Hz, 1H), 5.17 (s, 2H), 2.09 (s, 3H).

^13^C NMR (126 MHz, Acetone) δ 169.97, 169.94, 167.04, 141.42, 138.11, 137.77, 129.33, 126.14, 123.39, 123.27, 114.45, 65.03, 19.90.

HRMS: Theo. m/z 250.0538 Da; obs. m/z 250.0554 Da [M+H]. Error (6.4 ppm).

**ISFP20**

2-(2,6-difluorophenyl)isothiazol-3(2H)-one

Method: Synthetic procedure 1

^1^H NMR (500 MHz, CDCl_3_) δ 8.25 (d, J = 6.4 Hz, 1H), 7.33 (tt, J = 8.5, 6.1 Hz, 1H), 7.01 – 6.93 (m, 2H), 6.30 (d, J = 6.5 Hz, 1H).

^13^C NMR (126 MHz, CDCl_3_) δ 167.95, 160.71 – 158.64 (dd, J = 248.9, 3.5 Hz), 142.38, 131.05 (t, J = 9.8, 10.0 Hz), 113.35, 112.44 – 112.25 (dd, J = 3.9, 19.3 Hz) or 112.44 – 112.23 (dd, J = 19.4, 4.1 Hz), missing 1 x 13C resonance.

^19^F NMR (471 MHz, CDCl_3_) δ -115.89.

HRMS: Theo. m/z 214.0138 Da; obs. m/z 214.0144 Da [M+H]. Error (2.8 ppm).

**ISFP21**

2-(3-(hydroxymethyl)phenyl)isothiazol-3(2H)-one

Method: 3-(3-oxoisothiazol-2(3H)-yl)benzyl acetate (ISFP19), was underwent standard Zemplén deactylation conditions. Deacetylated material was neutralised with Amberlite IR-120 (H^+^) resin and filtered. Standard isolation and purification procedures were then applied to obtain the final product.

^1^H NMR (500 MHz, Acetone) δ 8.46 (d, J = 6.4 Hz, 1H), 7.51 (s, 1H), 7.41 (d, J = 8.0 Hz, 1H), 7.29 (t, J = 7.8 Hz, 1H), 7.18 (d, J = 7.6 Hz, 1H), 6.11 (d, J = 6.4 Hz, 1H), 4.56 (d, J = 5.9 Hz, 2H), 4.22 (t, J = 5.9 Hz, 1H).

^13^C NMR (126 MHz, Acetone) δ 205.22, 167.05, 144.13, 141.25, 137.52, 128.92, 124.72, 122.35, 121.90, 114.46, 63.30.

HRMS: Theo. m/z 208.0432 Da; obs. m/z 208.0442 Da [M+H]. Error (4.8 ppm).

**ISFP22**

2-phenethylisothiazol-3(2H)-one

Method: Synthetic procedure 1

^1^H NMR (500 MHz, CDCl_3_) δ 7.98 (d, J = 6.2 Hz, 1H), 7.30 (dd, J = 8.0, 6.6 Hz, 2H), 7.26 – 7.19 (m, 3H), 6.21 (d, J = 6.3 Hz, 1H), 4.02 (t, J = 7.4 Hz, 2H), 3.02 (t, J = 7.3 Hz, 2H).

^13^C NMR (126 MHz, CDCl_3_) δ 168.80, 138.90, 137.63, 128.87, 128.76, 128.65, 126.80, 126.46, 114.44, 45.22, 35.76.

HRMS: Theo. m/z 206.0640 Da; obs. m/z 206.0628 Da [M+H]. Error (-5.8 ppm).

**ISFP23**

ethyl 2-(3-oxoisothiazol-2(3H)-yl)acetate

Method: Synthetic procedure 1

^1^H NMR (400 MHz, CDCl_3_) δ 8.10 (d, J = 6.3 Hz, 1H), 6.21 (d, J = 6.3 Hz, 1H), 4.44 (s, 2H), 4.17 (q, J = 7.1 Hz, 2H), 1.22 (t, J = 7.1 Hz, 3H).

^13^C NMR (101 MHz, CDCl_3_) δ 169.03, 167.66, 140.58, 113.49, 61.90, 44.31, 14.07.

HRMS: Theo. m/z 188.0381 Da; obs. m/z 188.0387 Da [M+H]. Error (3.2 ppm).

**ISFP24**

ethyl 3-(3-oxoisothiazol-2(3H)-yl)propanoate

Method: Synthetic procedure 1

^1^H NMR (400 MHz, CDCl_3_) δ 8.06 (d, J = 6.3 Hz, 1H), 6.21 (d, J = 6.3 Hz, 1H), 4.16 (q, J = 7.1 Hz, 2H), 4.06 (t, J = 6.5 Hz, 2H), 2.72 (t, J = 6.5 Hz, 2H), 1.25 (t, J = 7.1 Hz, 3H).

^13^C NMR (101 MHz, CDCl_3_) δ 171.02, 168.90, 139.62, 114.20, 60.95, 39.75, 34.12, 14.11.

HRMS: Theo. m/z 202.0538 Da; obs. m/z 202.0530 Da [M+H]. Error (-4.0 ppm).

**ISFP25**

2-(2-ethoxyethyl)isothiazol-3(2H)-one

Method: Synthetic procedure 1

^1^H NMR (500 MHz, CDCl_3_) δ 8.07 (d, J = 6.3 Hz, 1H), 6.24 (d, J = 6.3 Hz, 1H), 3.98 (t, J = 5.0 Hz, 2H), 3.66 (t, J = 5.0 Hz, 2H), 3.54 (q, J = 7.0 Hz, 2H), 1.22 (t, J = 7.0 Hz, 3H).

^13^C NMR (126 MHz, CDCl_3_) δ 169.05, 139.97, 113.76, 68.89, 66.61, 43.84, 15.06.

HRMS: Theo. m/z 174.0589 Da; obs. m/z 174.0593 Da [M+H]. Error (2.3 ppm).

**ISFP26**

2-benzylisothiazol-3(2H)-one

Method: Synthetic procedure 1

^1^H NMR (500 MHz, CDCl_3_) δ 8.04 (d, J = 6.3 Hz, 1H), 7.41 – 7.23 (m, 6H + CHCl_3_), 6.29 (d, J = 6.2 Hz, 1H), 4.94 (s, 2H).

^13^C NMR (126 MHz, CDCl_3_) δ 168.88, 139.35, 136.28, 135.71, 128.88, 128.32, 114.45, 47.41.

HRMS: Theo. m/z 192.0483 Da; obs. m/z 192.0483 Da [M+H]. Error (0.0 ppm).

**ISFP27**

2-(3-chlorophenyl)-5-phenylisothiazol-3(2H)-one

Method: Synthetic procedure 3

^1^H NMR (500 MHz, Acetone) δ 7.81 (t, J = 2.1 Hz, 1H), 7.65 – 7.59 (m, 2H), 7.55 (ddd, J = 8.1, 2.1, 0.9 Hz, 1H), 7.49 – 7.43 (m, 3H), 7.40 (t, J = 8.1 Hz, 1H), 7.24 (ddd, J = 8.1, 2.0, 0.9 Hz, 1H), 6.61 (s, 1H).

^13^C NMR (126 MHz, Acetone) δ 205.15, 166.91, 155.91, 138.88, 134.26, 131.49, 130.70, 129.77, 129.54, 126.35, 125.86, 123.18, 121.47, 110.43, 29.37, 29.22, 29.06, 28.91, 28.75, 28.60, 28.45.

HRMS: Theo. m/z 288.025 Da; obs. m/z 288.0263 Da [M+H]. Error (4.5 ppm).

**ISFP28**

2-(3-chlorophenyl)-4-methylisothiazol-3(2H)-one

Method: Synthetic procedure 2

^1^H NMR (500 MHz, Acetone) δ 8.15 (t, J = 1.3 Hz, 1H), 7.79 (t, J = 2.1 Hz, 1H), 7.51 – 7.47 (m, 1H), 7.37 (t, J = 8.1 Hz, 1H), 7.21 (ddd, J = 8.0, 2.1, 0.9 Hz, 1H), 1.88 (m, 3H).

^13^C NMR (126 MHz, Acetone) δ 166.99, 139.51, 134.41, 134.19, 130.62, 126.15, 122.92, 121.23, 12.11.

HRMS: Theo. m/z 226.0093 Da; obs. m/z 226.0102 Da [M+H]. Error (4.0 ppm).

**ISFP30**

2-(3-chlorophenyl)-5-methylisothiazol-3(2H)-one

Method: Synthetic procedure 3

^1^H NMR (500 MHz, Acetone) δ 7.73 (t, J = 2.1 Hz, 1H), 7.42 (ddd, J = 8.2, 2.2, 1.0 Hz, 1H), 7.35 (t, J = 8.1 Hz, 1H), 7.19 (ddd, J = 8.0, 2.1, 1.0 Hz, 1H), 5.97 (q, J = 1.2 Hz, 1H), 2.38 (s, 3H).

^13^C NMR (126 MHz, Acetone) δ 166.92, 154.90, 139.06, 134.16, 130.60, 125.98, 122.91, 121.18, 113.29, 13.78.

HRMS: Theo. m/z 226.0093 Da; obs. m/z 226.0114 Da [M+H]. Error (9.3 ppm).

**References**

1. Stiers, K. M., Kain, B. N., Graham, A. C., and Beamer, L. J. (2016) Induced structural disorder as a molecular mechanism for enzyme dysfunction in phosphoglucomutase 1 deficiency. *Journal of Molecular Biology*. **428**, 1493–1505

2. Stiers, K. M., and Beamer, L. J. (2018) A hotspot for disease-associated variants of human PGM1 is associated with impaired ligand binding and loop dynamics. *Structure*. **26**, 1337-1345

3. Backe, P. H., Laerdahl, J. K., Kittelsen, L. S., Dalhus, B., Mørkrid, L., and Bjørås, M. (2020) Structural basis for substrate and product recognition in human phosphoglucomutase-1 (PGM1) isoform 2, a member of the α-D-phosphohexomutase superfamily. *Scientific Reports*. **10**, 1–14

4. Studer, G., Rempfer, C., Waterhouse, A. M., Gumienny, R., Haas, J., and Schwede, T. (2020) QMEANDisCo—distance constraints applied on model quality estimation. *Bioinformatics*. **36**, 1765–1771

5. Anandakrishnan, R., Aguilar, B., and Onufriev, A. V. (2012) H++ 3.0: automating pK prediction and the preparation of biomolecular structures for atomistic molecular modeling and simulations. *Nucleic Acids Research*. **40**, w537–w541

6. Myers, J., Grothaus, G., Narayanan, S., and Onufriev, A. (2006) A simple clustering algorithm can be accurate enough for use in calculations of pKs in macromolecules. *Proteins: Structure, Function and Genetics*. **63**, 928–938

7. Gordon, J. C., Myers, J. B., Folta, T., Shoja, V., Heath, L. S., and Onufriev, A. (2005) H++: a server for estimating pKas and adding missing hydrogens to macromolecules. *Nucleic Acids Research*. **33**, W368–W371

8. L’Enfant, M., Domon, J. M., Rayon, C., Desnos, T., Ralet, M. C., Bonnin, E., Pelloux, J., and Pau-Roblot, C. (2015) Substrate specificity of plant and fungi pectin methylesterases: identification of novel inhibitors of PMEs. *International Journal of Biological Macromolecules*. **81**, 681–691

9. Furdas, S. D., Hoffmann, I., Robaa, D., Herquel, B., Malinka, W., ͆wiątek, P., Akhtar, A., Sippl, W., and Jung, M. (2014) Pyrido- and benzisothiazolones as inhibitors of histone acetyltransferases (HATs). *MedChemComm*. **5**, 1856–1862

10. Ghizzoni, M., Haisma, H. J., and Dekker, F. J. (2009) Reactivity of isothiazolones and isothiazolone-1-oxides in the inhibition of the PCAF histone acetyltransferase. *European Journal of Medicinal Chemistry*. **44**, 4855–4861

11. Trevillyan, J. M., Chiou, X. G., Ballaron, S. J., Tang, Q. M., Buko, A., Sheets, M. P., Smith, M. L., Putman, C. B., Wiedeman, P., Tu, N., Madar, D., Smith, H. T., Gubbins, E. J., Warrior, U. P., Chen, Y. W., Mollison, K. W., Faltynek, C. R., and Djuric, S. W. (1999) Inhibition of p56(lck) tyrosine kinase by isothiazolones. *Archives of Biochemistry and Biophysics*. **364**, 19–29

12. Hayakawa, N., Nozawa, K., Ogawa, A., Kato, N., Yoshida, K., Akamatsu, K., Tsuchiya, M., Nagasaka, A., and Yoshida, S. (1999) Isothiazolone derivatives selectively inhibit telomerase from human and rat cancer cells in vitro. *Biochemistry*. **38**, 11501–11507

13. Chen, Y. H., Hsu, H. Y., Yeh, M. T., Chen, C. C., Huang, C. Y., Chung, Y. H., Chang, Z. F., Kuo, W. C., Chan, N. L., Weng, J. H., Chung, B. C., Chen, Y. J., Jian, C. B., Shen, C. C., Tai, H. C., Sheu, S. Y., and Fang, J. M. (2016) Chemical inhibition of human thymidylate kinase and structural insights into the phosphate binding loop and ligand-induced degradation. *Journal of Medicinal Chemistry*. **59**, 9906–9918

14. Cooper, I. R., McCarroll, A. J., McGarry, D., Kirkham, J., Pichowicz, M., Walker, R., Warrilow, C., Salisbury, A. M., Savage, V. J., Moyo, E., Forward, H., Cheung, J., Metzger, R., Gault, Z., Nelson, G., Hughes, D., Cao, S., Maclean, J., Charrier, C., Craighead, M., Best, S., Stokes, N. R., and Ratcliffe, A. J. (2016) Discovery and structure-activity relationships of a novel isothiazolone class of bacterial type II topoisomerase inhibitors. *Bioorganic & Medicinal Chemistry Letters*. **26**, 4179–4183

15. Abdul-Hay, S. O., Bannister, T. D., Wang, H., Cameron, M. D., Caulfield, T. R., Masson, A., Bertrand, J., Howard, E. A., McGuire, M. P., Crisafulli, U., Rosenberry, T. R., Topper, C. L., Thompson, C. R., Schurer, S. C., Madoux, F., Hodder, P., and Leissring, M. A. (2015) Selective targeting of extracellular insulin-degrading enzyme by quasi-irreversible thiol-modifying inhibitors. *ACS Chemical Biology*. **10**, 2716–2724

16. Amano, Y., Namatame, I., Tateishi, Y., Honboh, K., Tanabe, E., Niimi, T., and Sakashita, H. (2015) Structural insights into the novel inhibition mechanism of *Trypanosoma cruzi* spermidine synthase. *Acta Crystallographica Section D-Structural Biology*. **71**, 1879–1889

17. Bravo, Y., Teriete, P., Dhanya, R. P., Dahl, R., Lee, P. S., Kiffer-Moreira, T., Ganji, S. R., Sergienko, E., Smith, L. H., Farquharson, C., Millán, J. L., and Cosford, N. D. P. (2014) Design, synthesis and evaluation of benzoisothiazolones as selective inhibitors of PHOSPHO1. *Bioorganic and Medicinal Chemistry Letters*. **24**, 4308–4311

18. Lu, J., Vlamis-Gardikas, A., Kandasamy, K., Zhao, R., Gustafsson, T. N., Engstrand, L., Hoffner, S., Engman, L., and Holmgren, A. (2013) Inhibition of bacterial thioredoxin reductase: an antibiotic mechanism targeting bacteria lacking glutathione. *FASEB Journal*. **27**, 1394–1403

19. Dahl, R., Bravo, Y., Sharma, V., Ichikawa, M., Dhanya, R. P., Hedrick, M., Brown, B., Rascon, J., Vicchiarelli, M., Mangravita-Novo, A., Yang, L., Stonich, D., Su, Y., Smith, L. H., Sergienko, E., Freeze, H. H., and Cosford, N. D. P. (2011) Potent, selective, and orally available benzoisothiazolone phosphomannose isomerase inhibitors as probes for congenital disorder of glycosylation la. *Journal of Medicinal Chemistry*. **54**, 3661–3668

20. Vasan, M., Neres, J., Williams, J., Wilson, D. J., Teitelbaum, A. M., Remmel, R. P., and Aldrich, C. C. (2010) Inhibitors of the salicylate synthase (MbtI) from *Mycobacterium tuberculosis* discovered by high-throughput screening. *Chemmedchem*. **5**, 2079–2087

21. King, A. R., Lodola, A., Carmi, C., Fu, J., Mor, M., and Piomelli, D. (2009) A critical cysteine residue in monoacylglycerol lipase is targeted by a new class of isothiazolinone-based enzyme inhibitors. *British Journal of Pharmacology*. **157**, 974–983

22. Devos, R., Guisez, Y., Plaetinck, G., Cornelis, S., Tavernier, J., Vanderheyden, J., Foley, L. H., and Scheffler, J. E. (1994) Covalent modification of the interleukin-5 receptor by isothiazolones leads to inhibition of the binding of interleukin-5. *European Journal of Biochemistry*. **225**, 635–640

23. Burger, M., Schmitt-Koopmann, C., and Leroux, J. C. (2020) DNA unchained: two assays to discover and study inhibitors of the DNA clustering function of barrier-to-autointegration factor. *Scientific Reports*. **10**, 1–13

24. DeLano, W. L. (2004) Use of PyMOL as a communications tool for molecular science. *Abstracts of Papers of the American Chemical Society*. **228**, U313–U314

25. Dekker, F. J., Ghizzoni, M., van der Meer, N., Wisastra, R., and Haisma, H. J. (2009) Inhibition of the PCAF histone acetyl transferase and cell proliferation by isothiazolones. *Bioorganic and Medicinal Chemistry*. **17**, 460–466

26. Wisastra, R., Ghizzoni, M., Maarsingh, H., Minnaard, A. J., Haisma, H. J., and Dekker, F. J. (2011) Isothiazolones; Thiol-reactive inhibitors of cysteine protease cathepsin B and histone acetyltransferase PCAF. *Organic and Biomolecular Chemistry*. **9**, 1817–1822

27. Ramadas, K., and Srinivasan, N. (1992) Iron-ammonium chloride - a convenient and inexpensive reductant. *Synthetic Communications*. **22**, 3189-3195

28. Bellamy, F. D., and Ou, K. (1984) Selective reduction of aromatic nitro compounds with stannous chloride in non acidic and non aqueous medium. *Tetrahedron Letters*. **25**, 839-842

29. Chen, M. Y., Pannecoucke, X., Jubault, P., and Besset, T. (2019) Access to Isothiazolones from simple acrylamides by Pd-catalyzed C-H bond activation. *Journal of Organic Chemistry*. **84**, 13194–13202

30. Wang, S., Hu, B., and Zhang, X. (2019) Copper‐catalyzed thioannulation of propynamides with sodium sulfide for the synthesis of isothiazol‐3‐ones. *Advanced Synthesis & Catalysis*. **361**, 1459-1462
