# Supplementary material for: Genetic validation of Aspergillus fumigatus phosphoglucomutase as a viable therapeutic target in invasive aspergillosis
Source: J Biol Chem. 2022 Apr 30;298(6):102003. doi: 10.1016/j.jbc.2022.102003 (PMC9168620; doi:10.1016/j.jbc.2022.102003)
Supplement: Table_S1 [file mmc3.docx]

| **ISFP1 (µM)** | ***K*_m_ (µM)** | ***k*_cat_ (s^-1^)** | ***k*_cat_/*K*_m_ (s^-1^ M^-1^)** |
| --- | --- | --- | --- |
| 0 | 31.7 ± 7.5 | 90.5 ± 6.7 | 2.9 ☓ 10^6^ |
| 0.3 | 20.8 ± 5.5 | 57.5 ± 4.0 | 2.8 ☓ 10^6^ |
| 0.6 | 17.2 ± 6.7 | 50.1 ± 4.8 | 2.9 ☓ 10^6^ |
| 1.3 | 11.8 ± 6.7 | 36.9 ± 4.3 | 3.1 ☓ 10^6^ |
| 2.5 | 14.8 ± 3.8 | 28.1 ± 1.7 | 1.9 ☓ 10^6^ |
| 5 | 17.5 ± 5.9 | 19.7 ± 1.6 | 1.1 ☓ 10^6^ |
| 10 | 14.3 ± 5.1 | 13.0 ± 1.0 | 9.1 ☓ 10^5^ |
| 20 | 13.1 ± 5.7 | 9.1 ± 0.9 | 6.9 ☓ 10^5^ |
