# Supplementary material for: Genetic validation of Aspergillus fumigatus phosphoglucomutase as a viable therapeutic target in invasive aspergillosis
Source: J Biol Chem. 2022 Apr 30;298(6):102003. doi: 10.1016/j.jbc.2022.102003 (PMC9168620; doi:10.1016/j.jbc.2022.102003)
Supplement: Table_S2 [file mmc4.docx]

| ***Af*PGM** |  |  |  |  |  |
| --- | --- | --- | --- | --- | --- |
| Cys | **C131** | **C242** | **C353** | **C364** |  |
| pKa | 10.2 | 9.7 | 11.1 | >12.0 |  |
| ***Hs*PGM** |  |  |  |  |  |
| Cys | **C101** | **C160** | **C238** | **C251** | **C374** |
| pKa | >12.0 | >12.0 | >12.0 | >12.0 | >12.0 |
