# Supplementary material for: Genetic validation of Aspergillus fumigatus phosphoglucomutase as a viable therapeutic target in invasive aspergillosis
Source: J Biol Chem. 2022 Apr 30;298(6):102003. doi: 10.1016/j.jbc.2022.102003 (PMC9168620; doi:10.1016/j.jbc.2022.102003)
Supplement: Table_S3 [file mmc5.docx]

| ID | R_1_ | R_2_ | R_3_ | *IC_50_* (μM) | | |
| --- | --- | --- | --- | --- | --- | --- |
|  |  |  |  | *Af*PGM | *Af*PGM_C353_ | *Hs*PGM |
|  |  |  |  |  |  |  |
| ISFP1 | 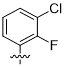 | H | H | 2.0 | 3.0 | 80.0 |
| ISFP2 | 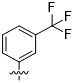 | H | H | 9.0 | 10.0 | > 100^b^ |
| ISFP3 | 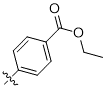 | H | H | 2.0 | 9.8 | > 100 ^b^ |
| ISFP4 | 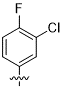 | H | H | 3.7 | 8.8 | > 100^b^ |
| ISFP5 | 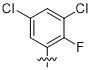 | H | H | 0.8 | 7.8 | 10.0 |
| ISFP6 | 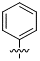 | H | H | 20.0 | 21.0 | > 400^b^ |
| ISFP7 | 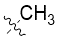 | H | H | > 400^b^ | ND | ND |
| ISFP8 | 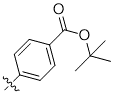 | H | H | 3.1 | 6.7 | > 100^b^ |
| ISFP9 | 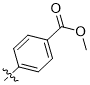 | H | H | 2.0 | 5.7 | > 100^b^ |
| ISFP10 | 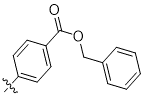 | H | H | 2.0 | 3.6 | > 100^b^ |
| ISFP11 | 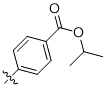 | H | H | 3.1 | 6.0 | > 100^b^ |
| ISFP12 | 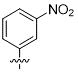 | H | H | 5.7 | 9.5 | >100^b^ |
| ISFP13 | 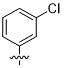 | H | H | 5.8 | 12.2 | 100.0 |
| ISFP14 | 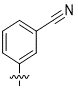 | H | H | 5.7 | 9.5 | > 100^b^ |
| ISFP15 | 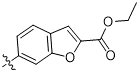 | H | H | 2.3 | 15.0 | > 100^b^ |
| ISFP16 | 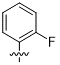 | H | H | 21.0 | 23.0 | > 400^b^ |
| ISFP17 | 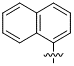 | H | H | 3.3 | 16.0 | > 100^b^ |
| ISFP18 | 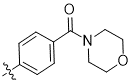 | H | H | 19.0 | 23.0 | > 100^b^ |
| ISFP19 | 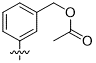 | H | H | 36.0 | 115.0 | > 400^b^ |
| ISFP20 | 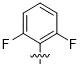 | H | H | 19.0 | 14.0 | 86.0 |
| ISFP21 | 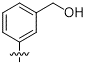 | H | H | 23.0 | 25.0 | > 400^b^ |
| ISFP22 | 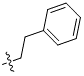 | H | H | no inhibition | ND | ND |
| ISFP23 | 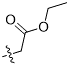 | H | H | no inhibition | ND | ND |
| ISFP24 | 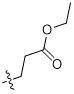 | H | H | no inhibition | ND | ND |
| ISFP25 | 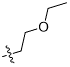 | H | H | no inhibition | ND | ND |
| ISFP26 | 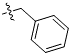 | H | H | no inhibition | ND | ND |
| ISFP27 | 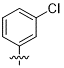 | 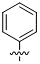 | H | no inhibition | ND | ND |
| ISFP28 | 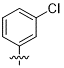 | H | 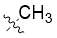 | no inhibition | ND | ND |
| ISFP30 | 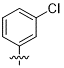 | 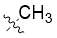 | H | 5.2 | no inhibition | no inhibition |
