# Supplementary material for: Genetic validation of Aspergillus fumigatus phosphoglucomutase as a viable therapeutic target in invasive aspergillosis
Source: J Biol Chem. 2022 Apr 30;298(6):102003. doi: 10.1016/j.jbc.2022.102003 (PMC9168620; doi:10.1016/j.jbc.2022.102003)
Supplement: Table_S4 [file mmc6.docx]

| **Target** | **Function** | **Reference** |
| --- | --- | --- |
| Phosphoglucomutase | UDP-Glc biosynthesis | This study |
| ﻿pectin methylesterase | Cell wall remodelling | (8) |
| ﻿Histone acetyltransferase | Gene transcription | (9, 10) |
| ﻿p56^lck^ tyrosine kinase | Activation of ﻿T lymphocytes | (11) |
| ﻿Telomerase | Cell ﻿proliferation | (12) |
| ﻿Thymidylate kinase | ﻿dTTP biosynthesis | (13) |
| ﻿Type II topoisomerase | Managing DNA supercoils | (14) |
| ﻿Insulin-degrading enzyme | Insulin homeostasis | (15) |
| ﻿Spermidine synthase | ﻿Polyamine–trypanothione pathway | (16) |
| ﻿Orphan phosphatase | Phosphate homeostasis | (17) |
| ﻿Thioredoxin reductase | DNA biosynthesis | (18) |
| ﻿Phosphomannose Isomerase | Glycosylation | (19) |
| ﻿Salicylate Synthase | ﻿Iron acquisition | (20) |
| ﻿Monoacylglycerol lipase | Lipid metabolism | (21) |
| ﻿Interleukin-5 receptor | Differentiation and growth of WBCs | (22) |
| ﻿BAF protein | ﻿Cytoplasmic DNA defense | (23) |
|  |  |  |
