# Supplementary material for: Genetic validation of Aspergillus fumigatus phosphoglucomutase as a viable therapeutic target in invasive aspergillosis
Source: J Biol Chem. 2022 Apr 30;298(6):102003. doi: 10.1016/j.jbc.2022.102003 (PMC9168620; doi:10.1016/j.jbc.2022.102003)
Supplement: Table_S5 [file mmc7.docx]

| **Description** | **Primers** | **Sequence (5' to 3')** |
| --- | --- | --- |
| Conditional mutant | P1 | GTACCCGGG ACTCCCTTCAAATCCCTTACTTTTCTGC |
|  | P2 | TCCTCTAGA ACCGTAGATCATGTTGCGGTCACCG |
| *pgm* gene | P3 | ATGTCGGTCCAGACGGTCTCCATTC |
|  | P4 | TTAAGTCTTGACATCAGGGTCCTCCC |
| *pyr-4* | P5 | AAACGCAAATCACAACAGCCAAC |
|  | P6 | CTATGCCAGACGCTCCCGG |
| P*alcA*-gene-downstream | P7 | CTGAAAAGCTGATTGTGATAGTTCCCAC |
|  | P8 | CCATCTCCATCTATGATTTCGCGACC |
| Probe 1 for Southern blot | P9 | GTACCCGGG ACTCCCTTCAAATCCCTTACTTTTCTGC |
|  | P10 | TCCTCTAGA ACCGTAGATCATGTTGCGGTCACCG |
| Probe 2 for Southern blot | P11 | CGGTGCCCTTGATGTTGACAAGG |
|  | P12 | AAACGCA AATCACAACAGCCAAC |
| Full length *Af*PGM | P13 | AAAGGATCCATGTCGGTCCAGACGGTCTCCATTCAG |
|  | P14 | AAAGCGGCCGCTTAAGTCTTGACATCAGGGTCCTCCCG |
| Full length *Ca*PGM | P15 | AAAGGATCCATGTCAGAATTGTCAATCAAGACAATC |
|  | P16 | AAAGCGGCCGCTTACGTGCGTACGTCGGGTTCTTCC |
| Full length *Hs*PGM | P17 | AAAGGATCCATGGTGAAGATCGTGACAGTTAAGACC |
|  | P18 | AAAGCGGCCGCTTAGGTGATGACAGTGGGTGCAGTGC |
