# Supplementary figures and images for: Genetic validation of Aspergillus fumigatus phosphoglucomutase as a viable therapeutic target in invasive aspergillosis
Source: J Biol Chem. 2022 Apr 30;298(6):102003. doi: 10.1016/j.jbc.2022.102003 (PMC9168620; doi:10.1016/j.jbc.2022.102003)

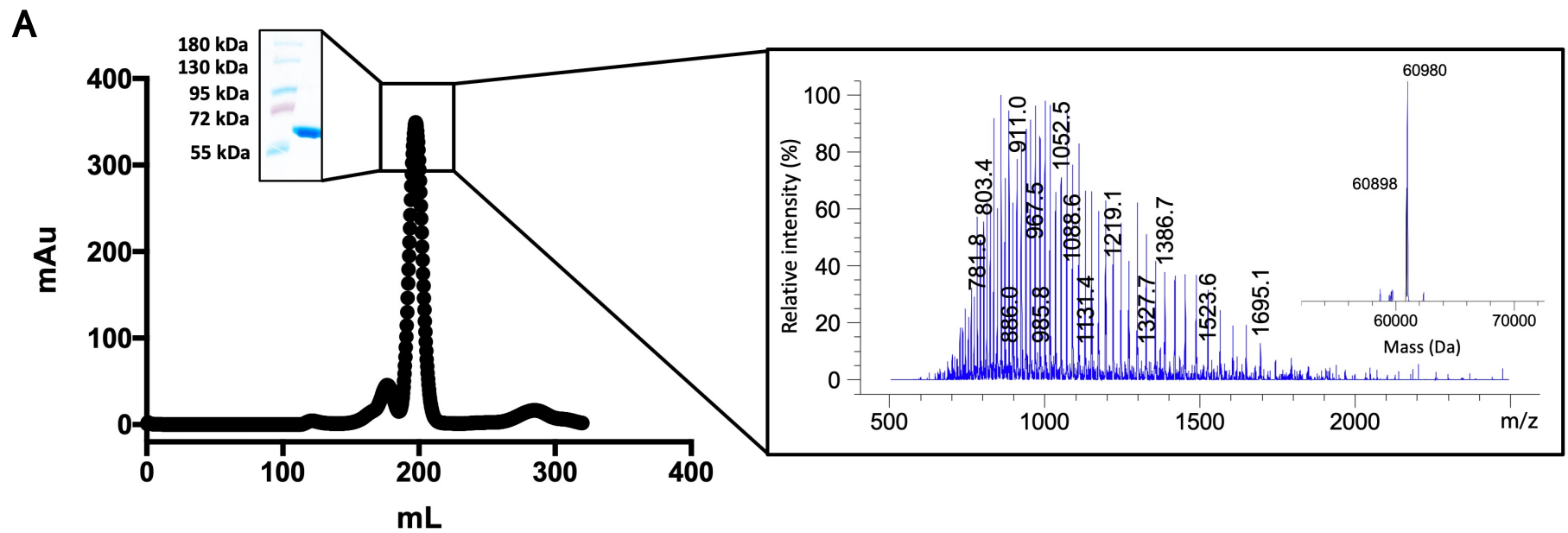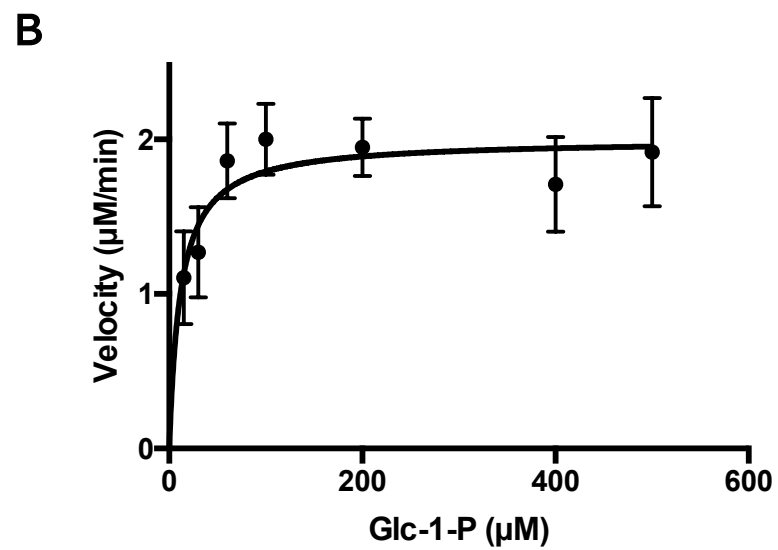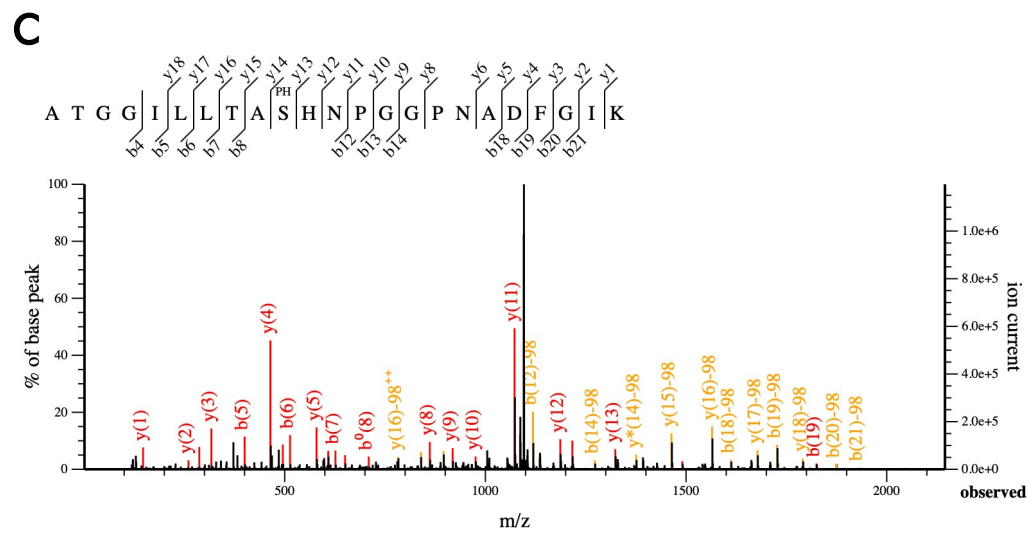

Supplement: Figure_S1 [file mmc8.pdf]

**A**

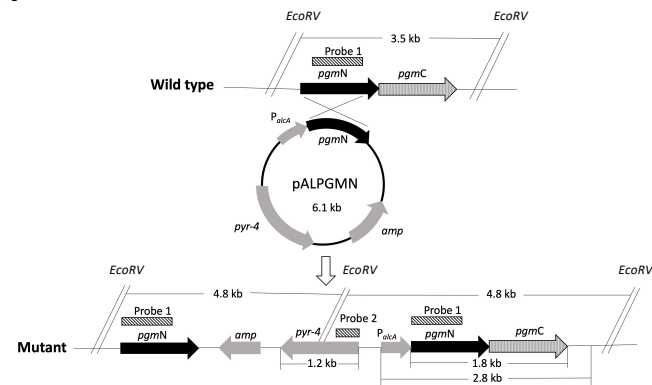

**B**

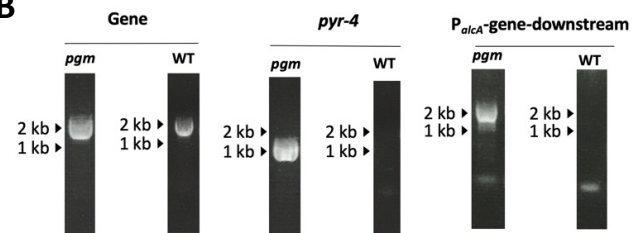

**C**

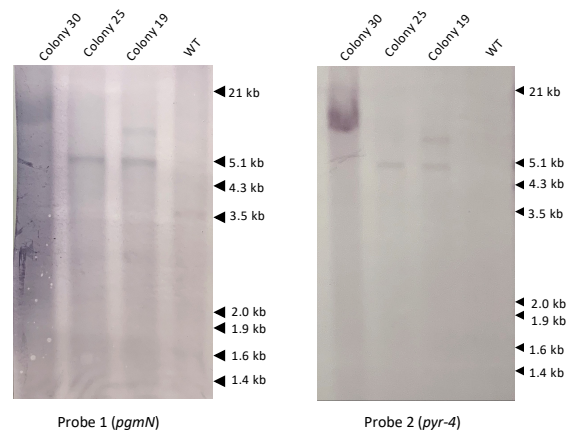

Supplement: Figure_S2 [file mmc9.pdf]

**A**

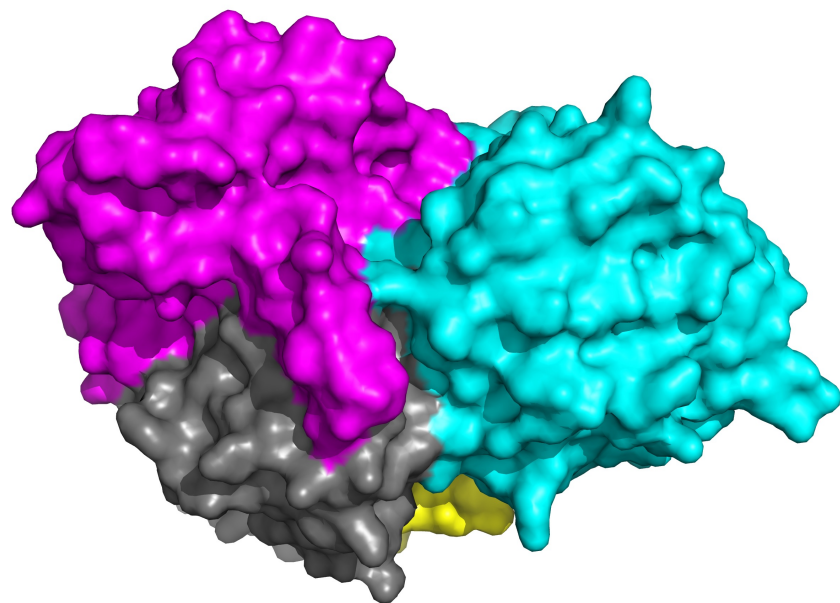

**B**

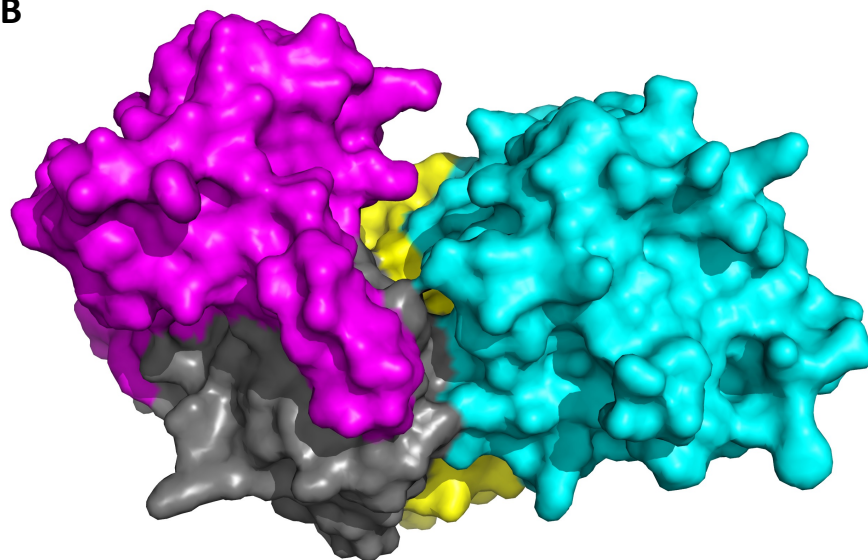

**C**

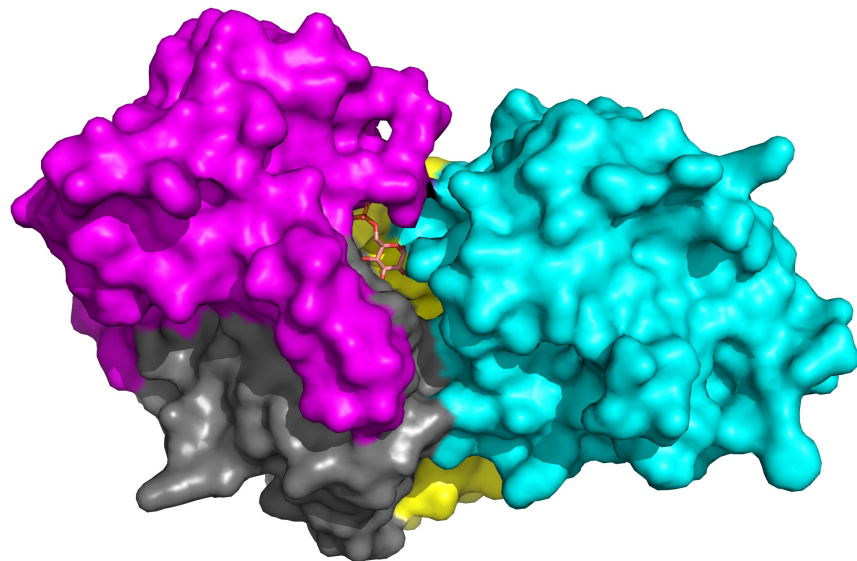

**D**

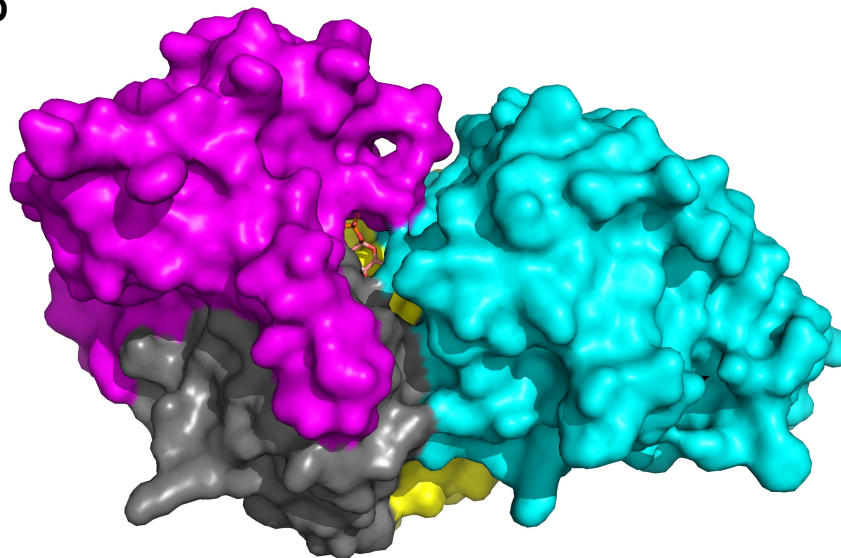

Supplement: Figure_S3 [file mmc10.pdf]

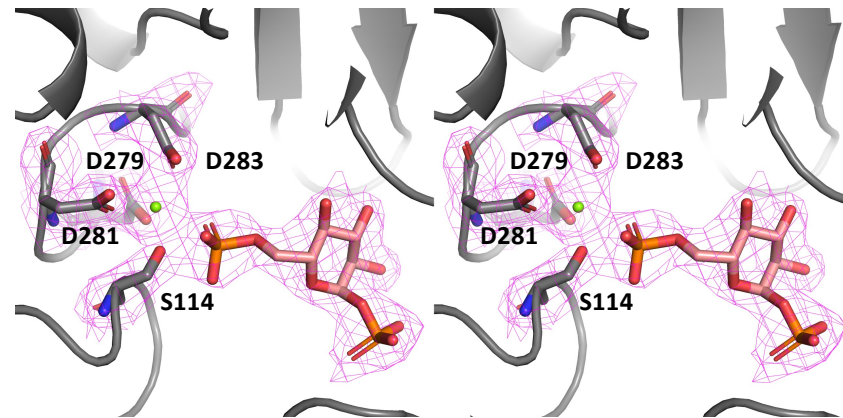

Supplement: Figure_S5 [file mmc12.pdf]

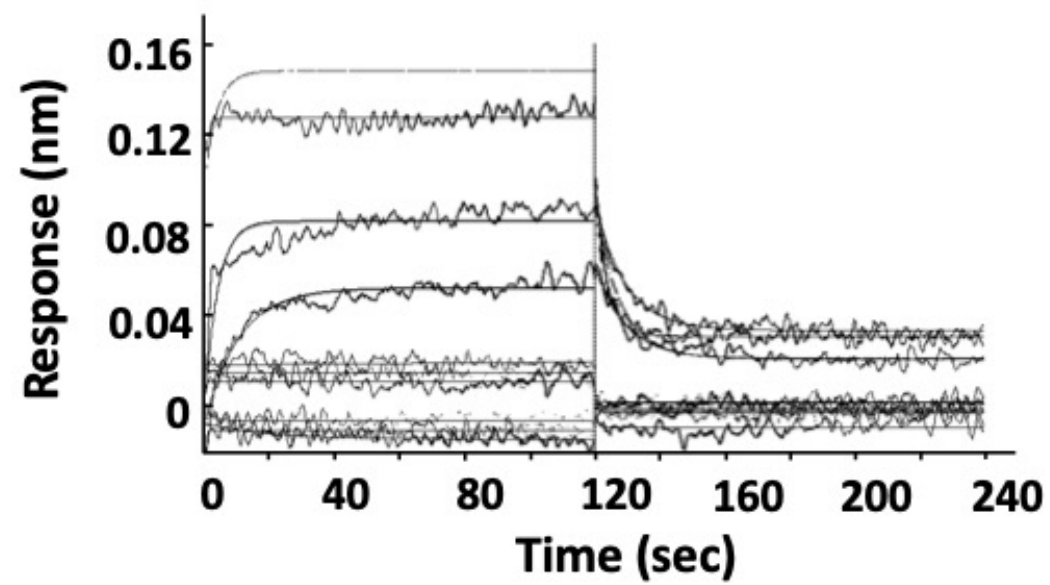

Supplement: Figure_S6 [file mmc13.pdf]

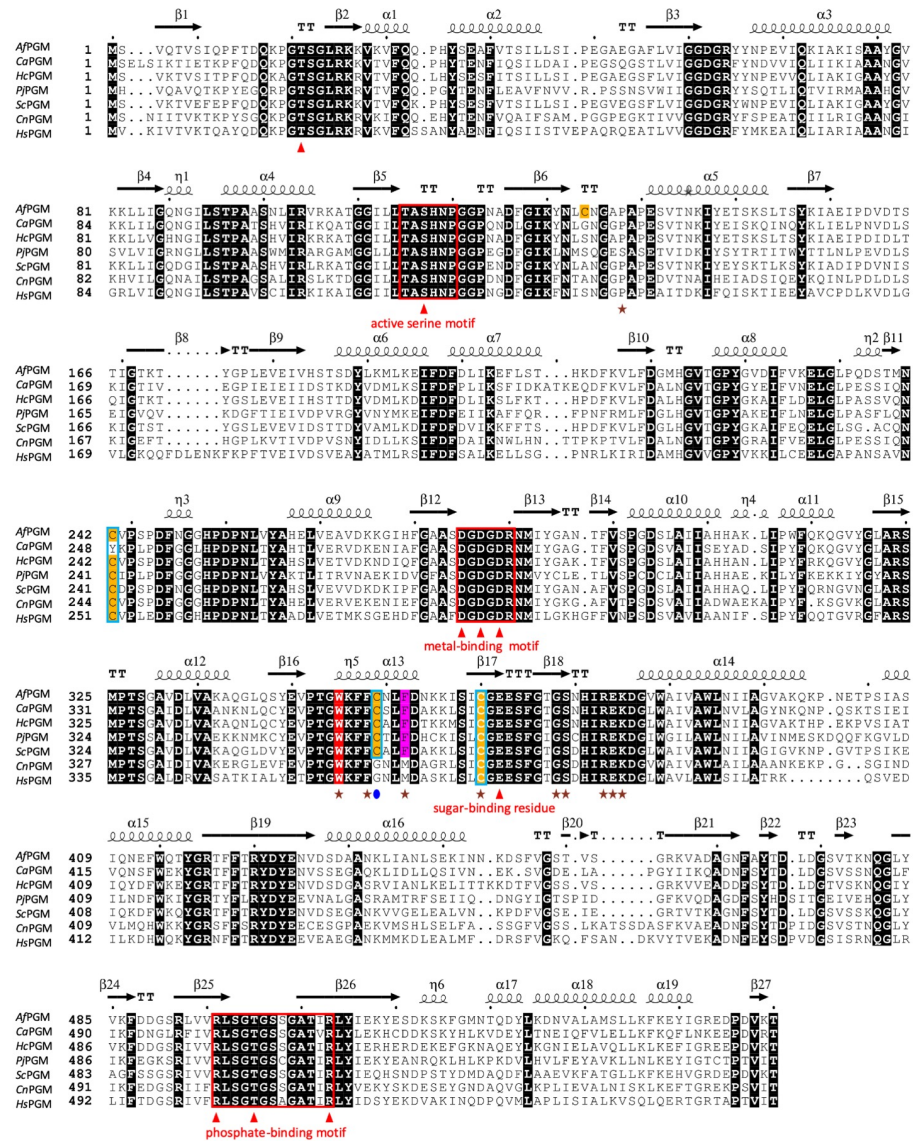

Supplement: Figure_S7 [file mmc14.pdf]

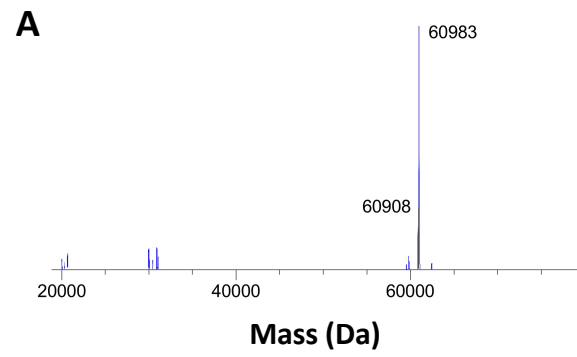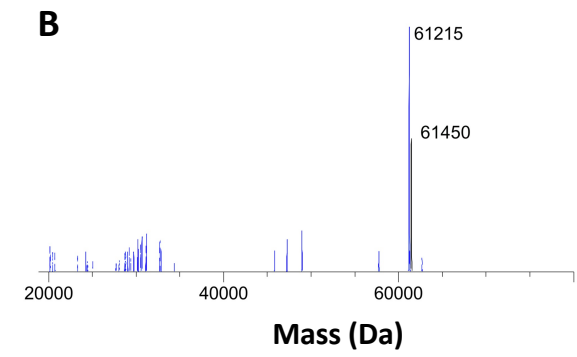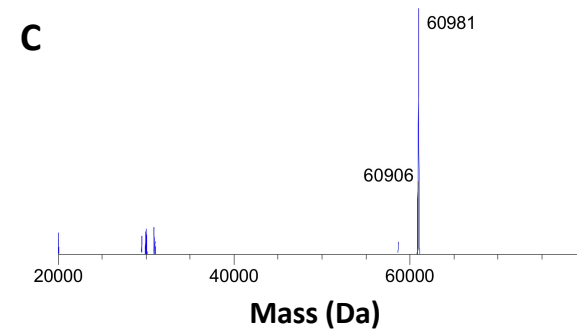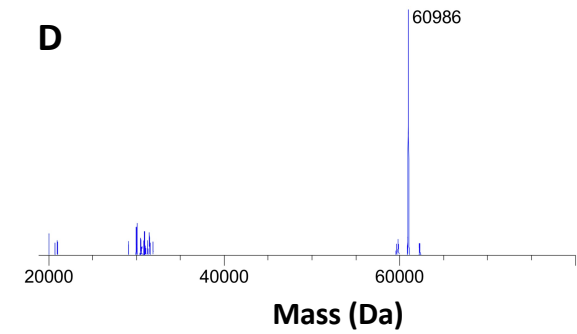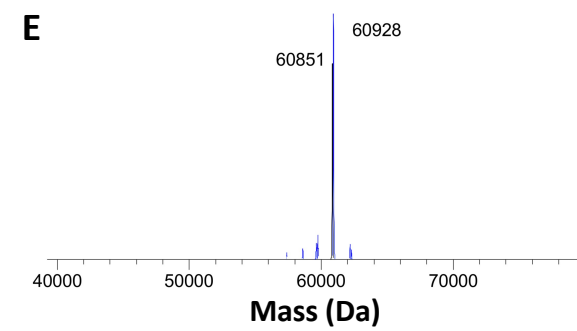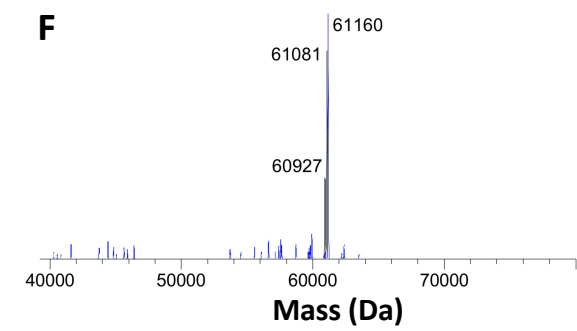

Supplement: Figure_S8 [file mmc15.pdf]

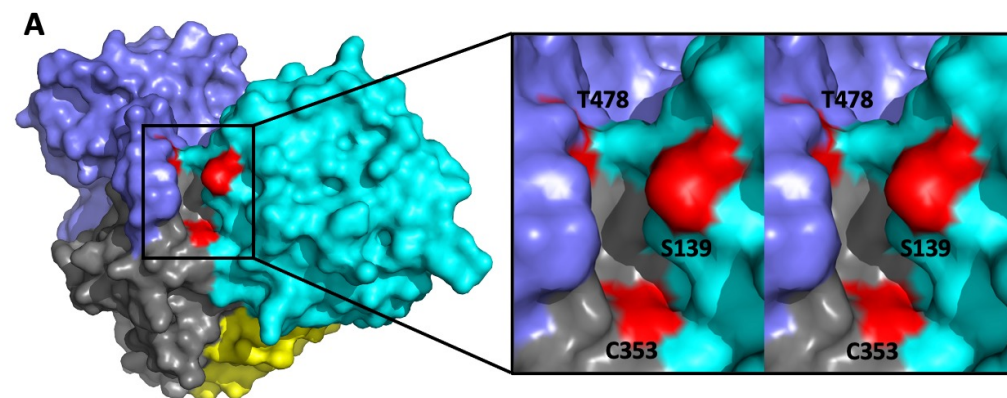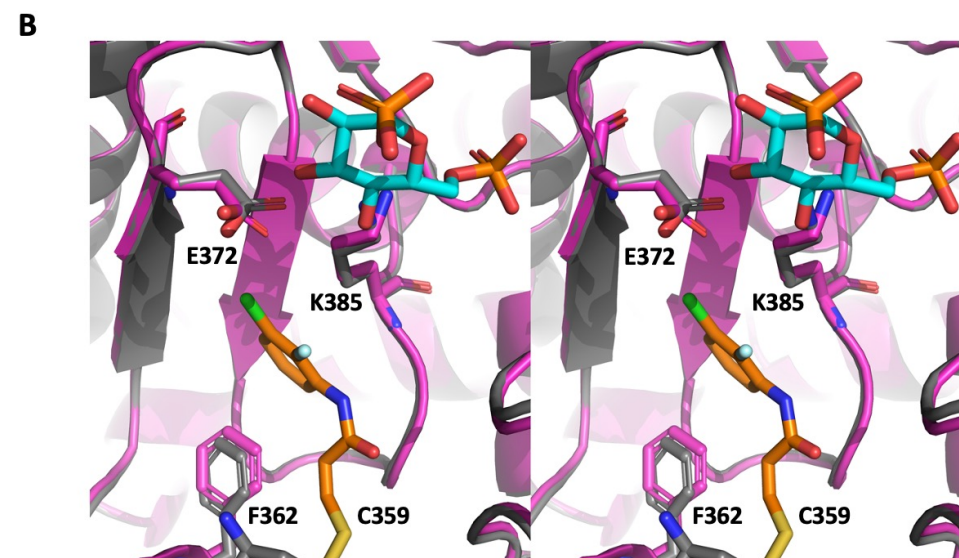

Supplement: Figure_S9 [file mmc16.pdf]

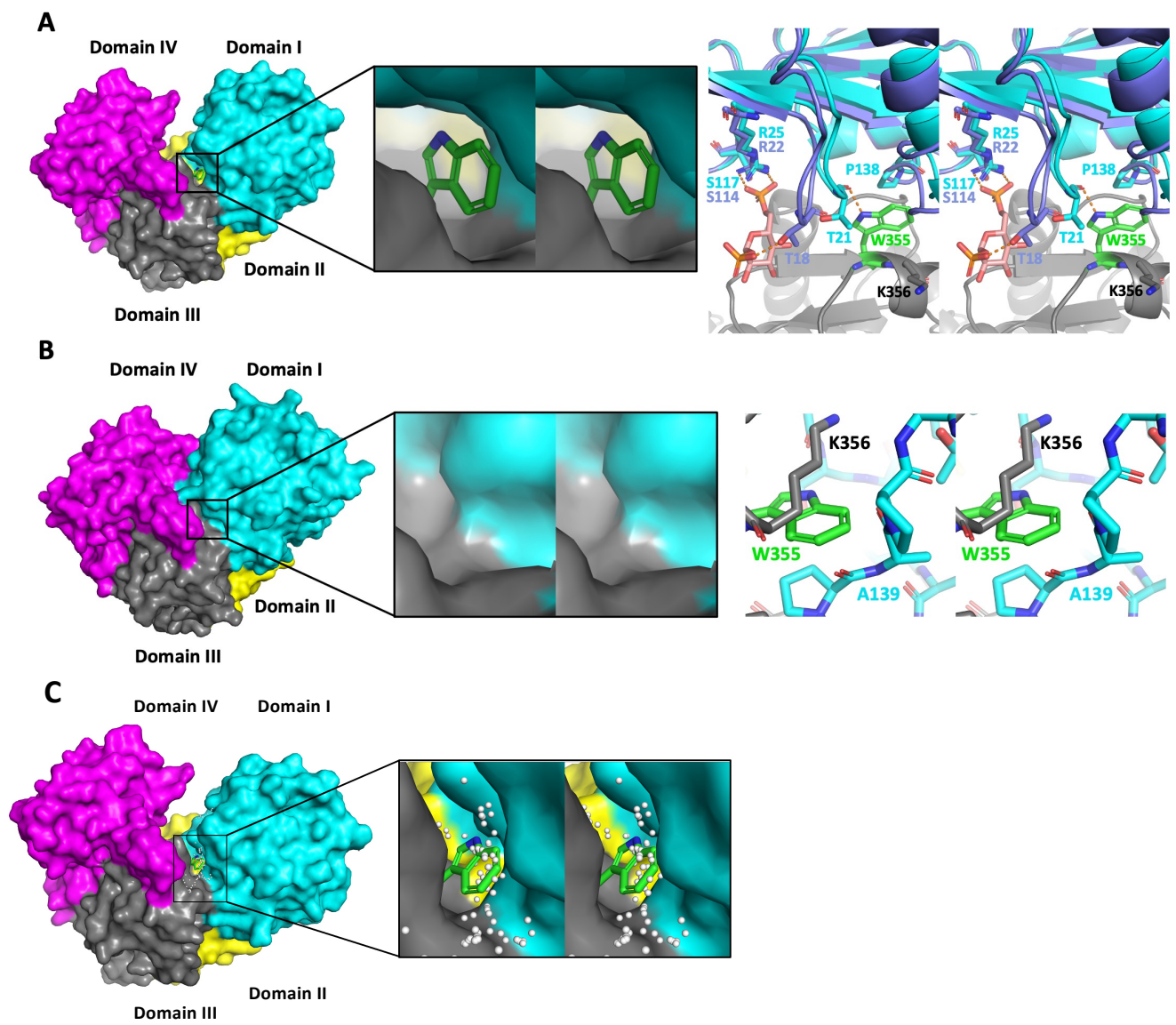

Supplement: Figure_S10 [file mmc17.pdf]

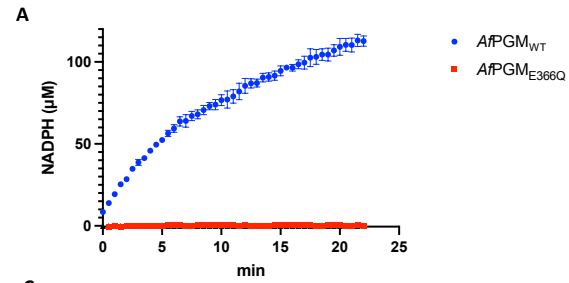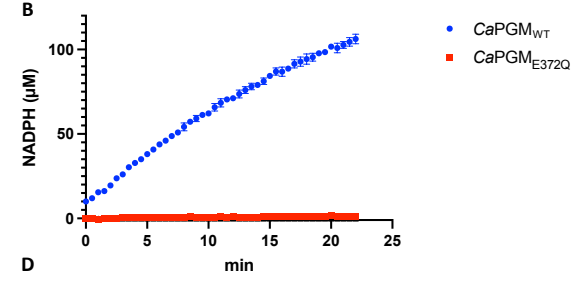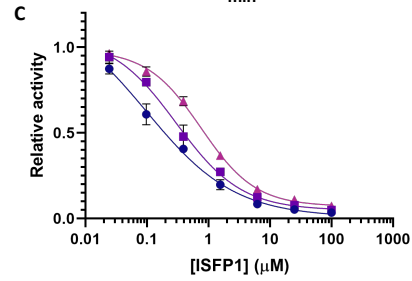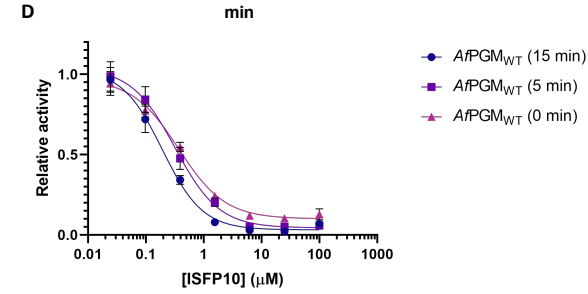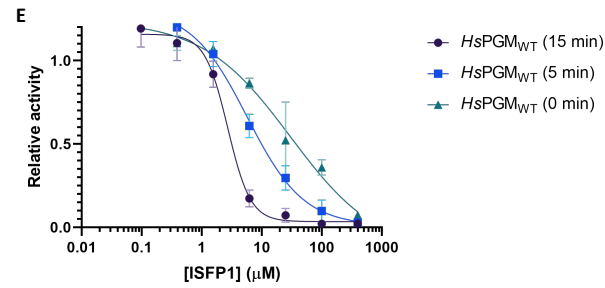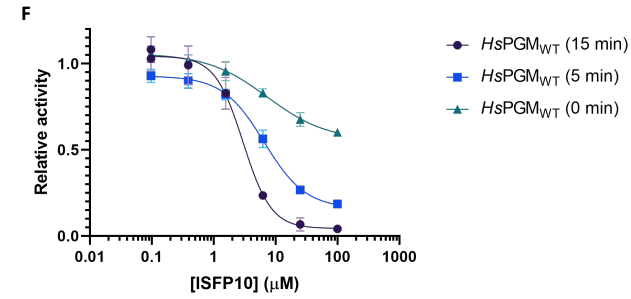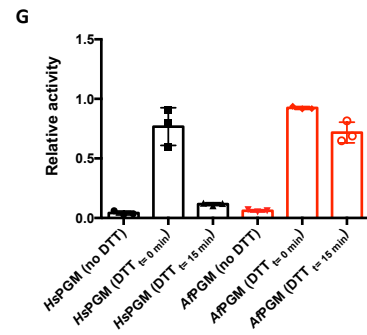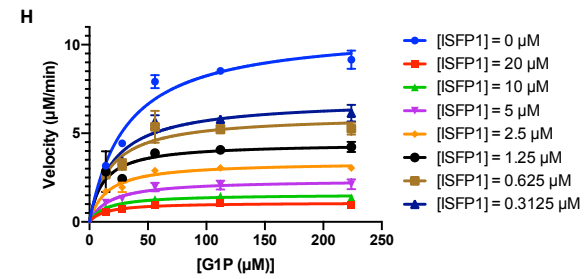

Supplement: Figure_S11 [file mmc18.pdf]

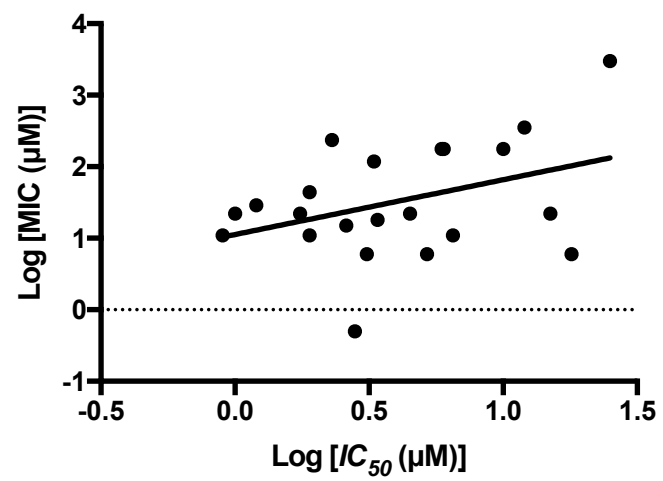

Supplement: Figure_S12 [file mmc19.pdf]
