# Supplementary material for: Genetic validation of Aspergillus fumigatus phosphoglucomutase as a viable therapeutic target in invasive aspergillosis
Source: J Biol Chem. 2022 Apr 30;298(6):102003. doi: 10.1016/j.jbc.2022.102003 (PMC9168620; doi:10.1016/j.jbc.2022.102003)

ISFP5 1H NMR

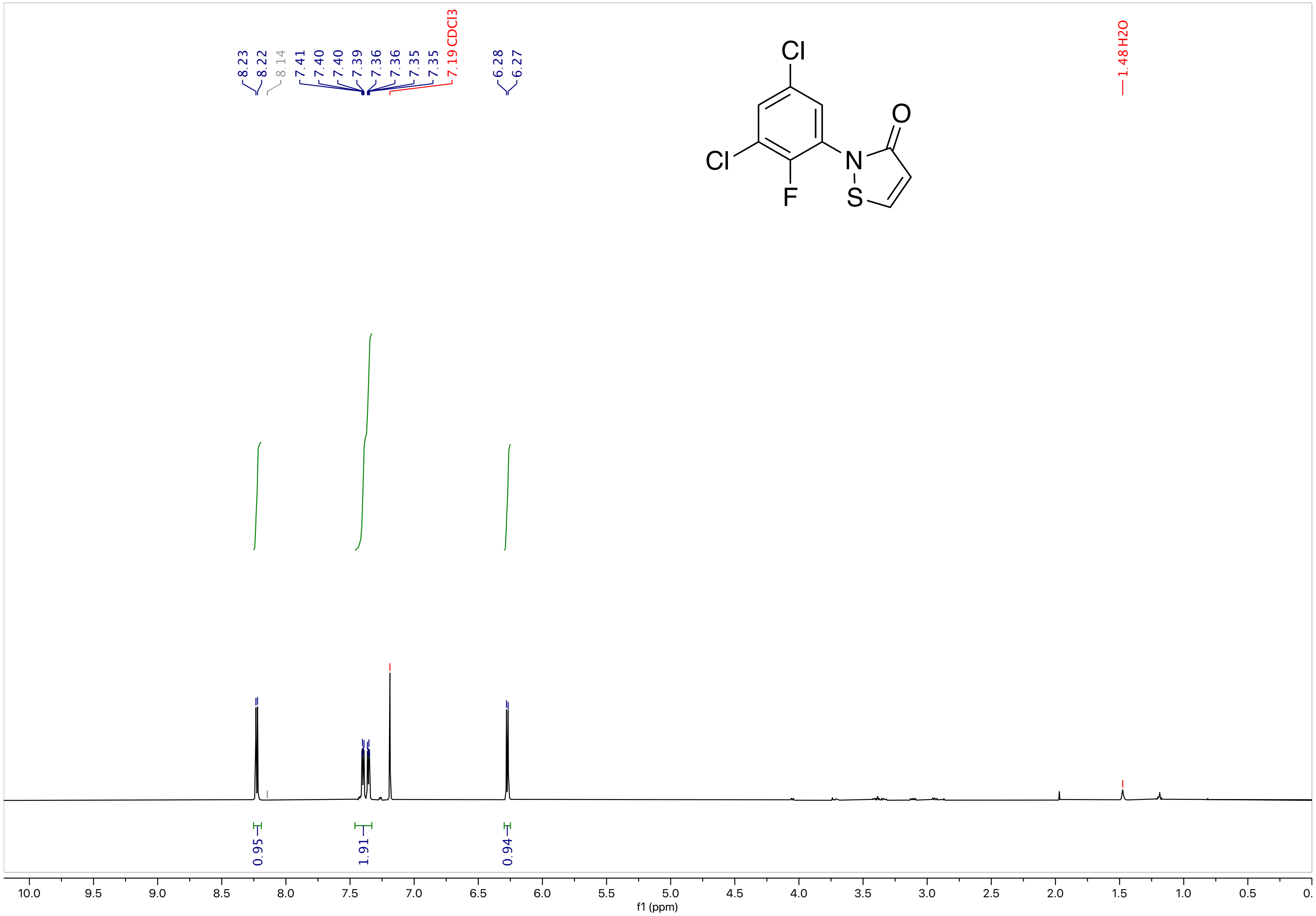
8.23
8.22
8.14
7.41
7.40
7.40
7.39
7.36
7.36
7.35
7.35
7.19 CDCl3
6.28
6.27
1.48 H2O
Cl
Cl
F
N
S
O
0.95
1.91
0.94
10.0
9.5
9.0
8.5
8.0
7.5
7.0
6.5
6.0
5.5
5.0
4.5
4.0
3.5
3.0
2.5
2.0
1.5
1.0
0.5
0.
f1 (ppm)

ISFP5 13C NMR

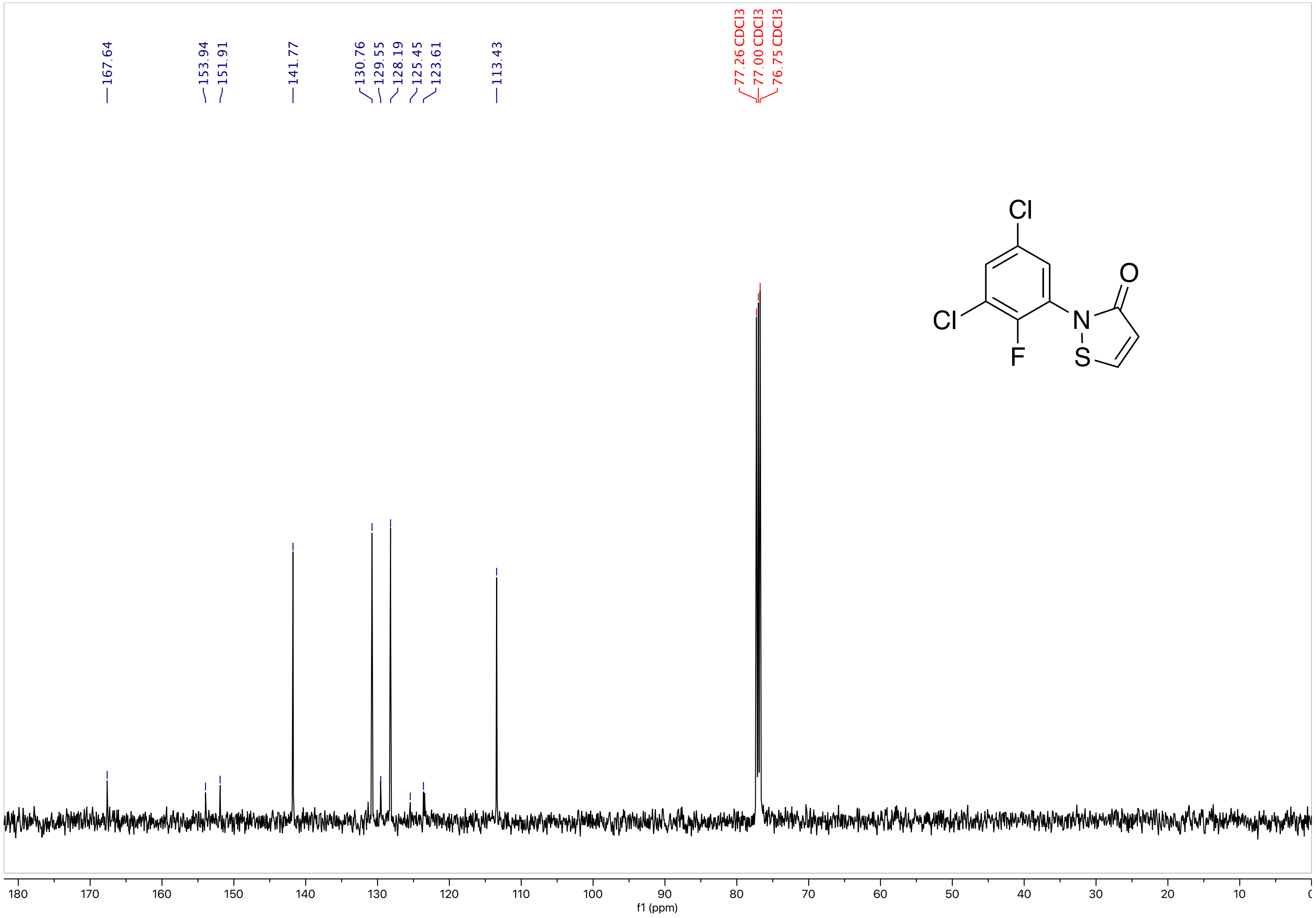

ISFP5 19F NMR

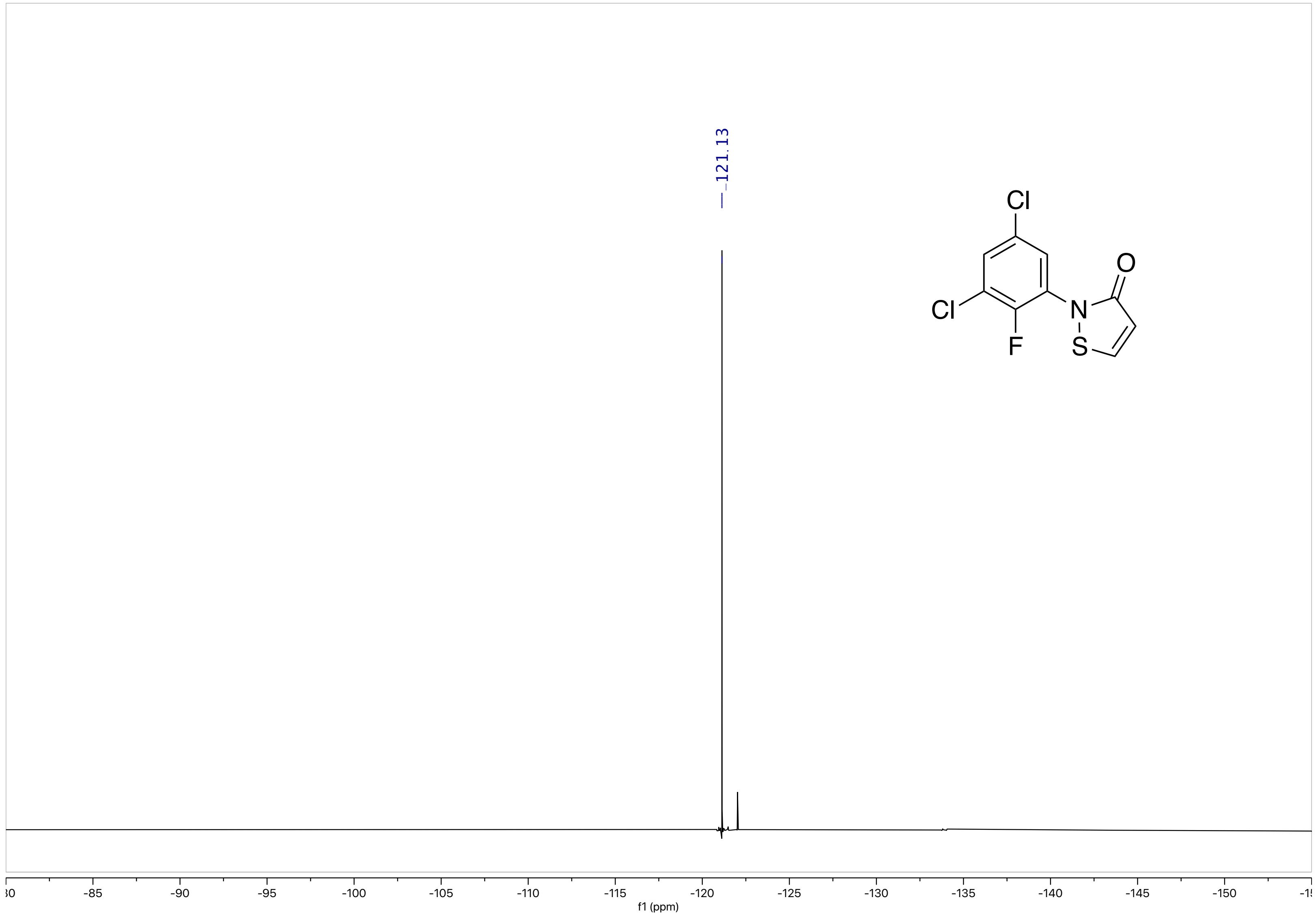

ISFP8 1H NMR

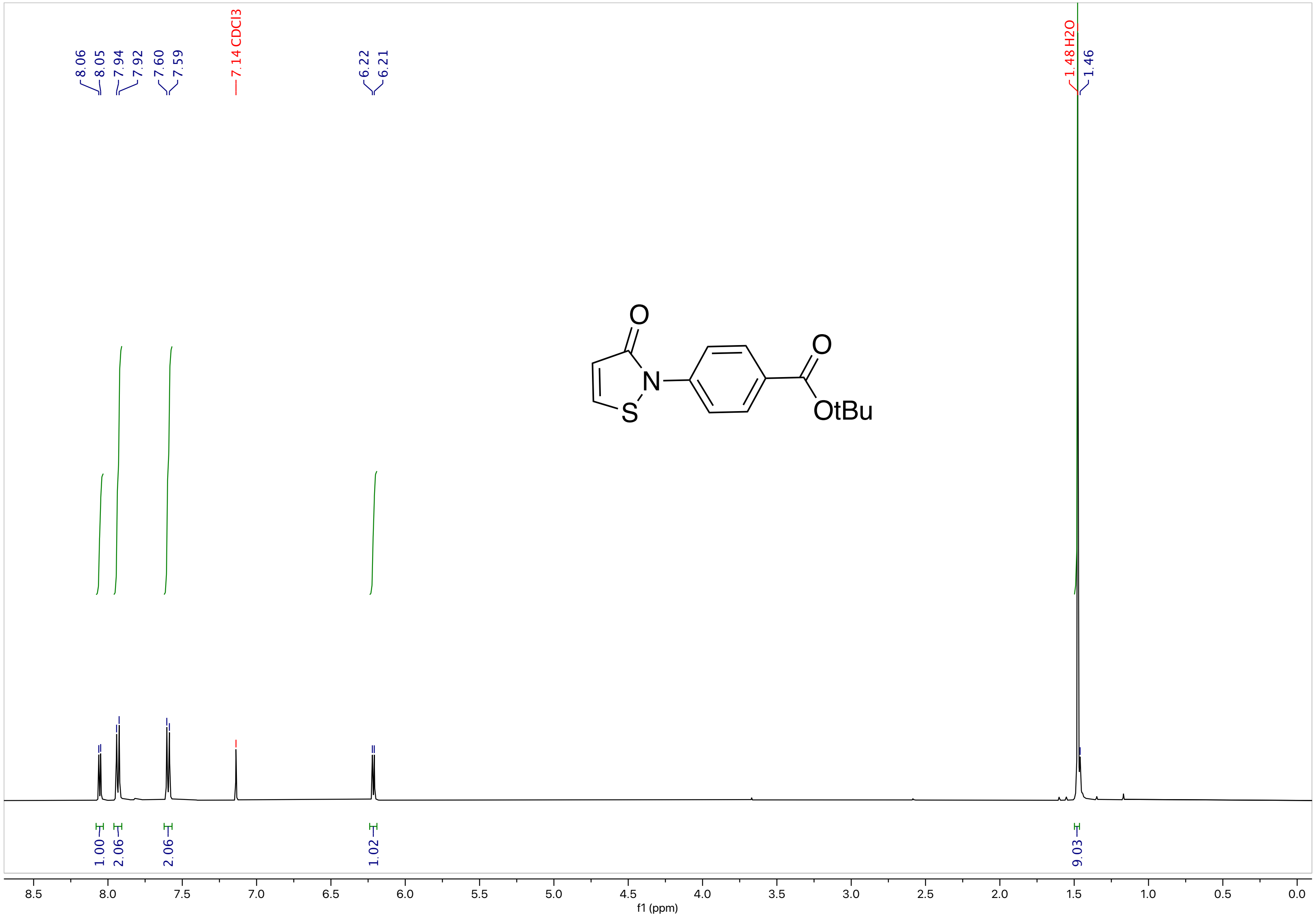
8.06
8.05
7.94
7.92
7.60
7.59
7.14 CDCl3
6.22
6.21
1.48 H2O
1.46
O
N
S
O
OtBu
1.00
2.06
2.06
1.02
9.03
8.5
8.0
7.5
7.0
6.5
6.0
5.5
5.0
4.5
4.0
3.5
3.0
2.5
2.0
1.5
1.0
0.5
0.0
f1 (ppm)

ISFP8 13C NMR

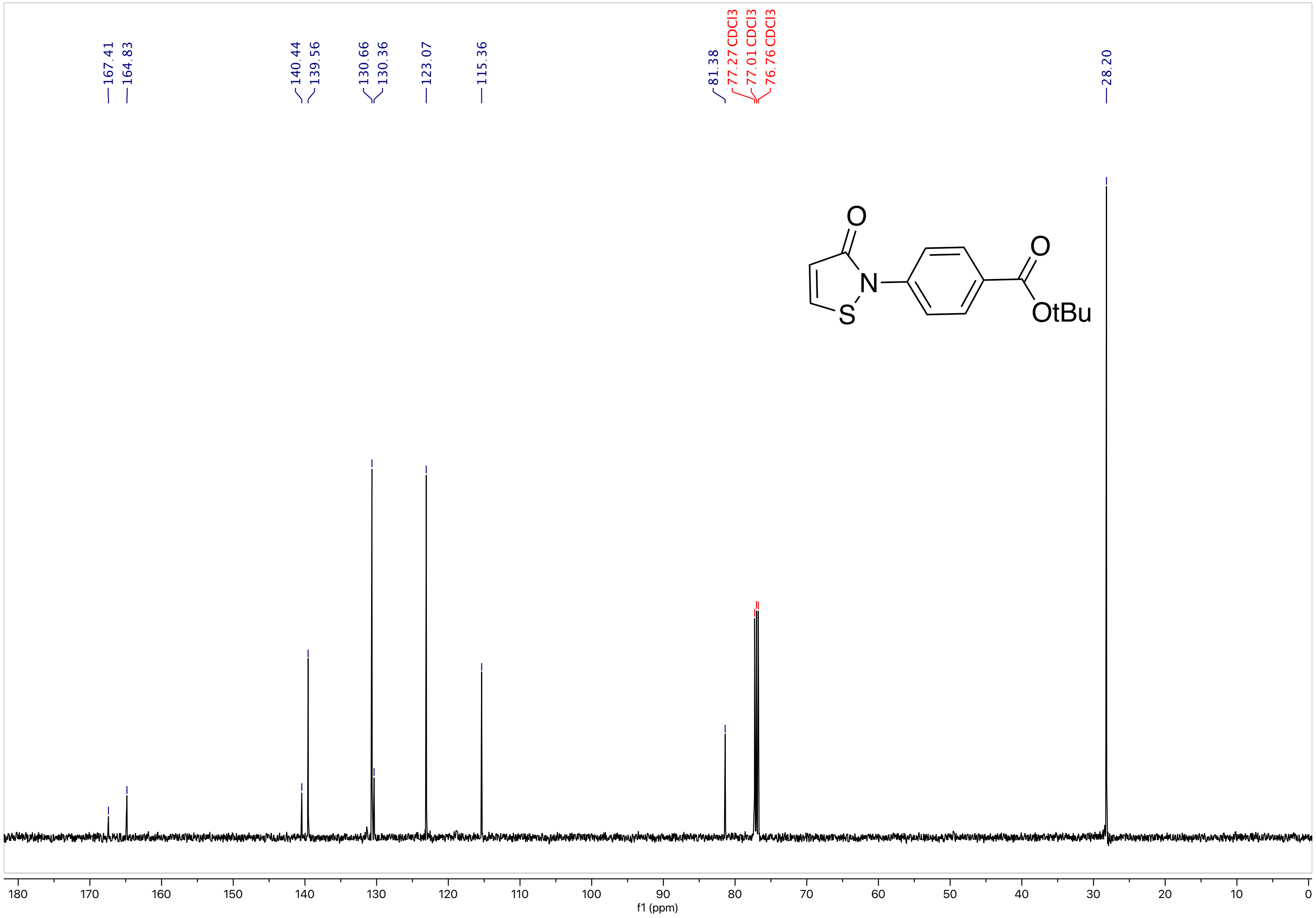

ISFP10 1H NMR

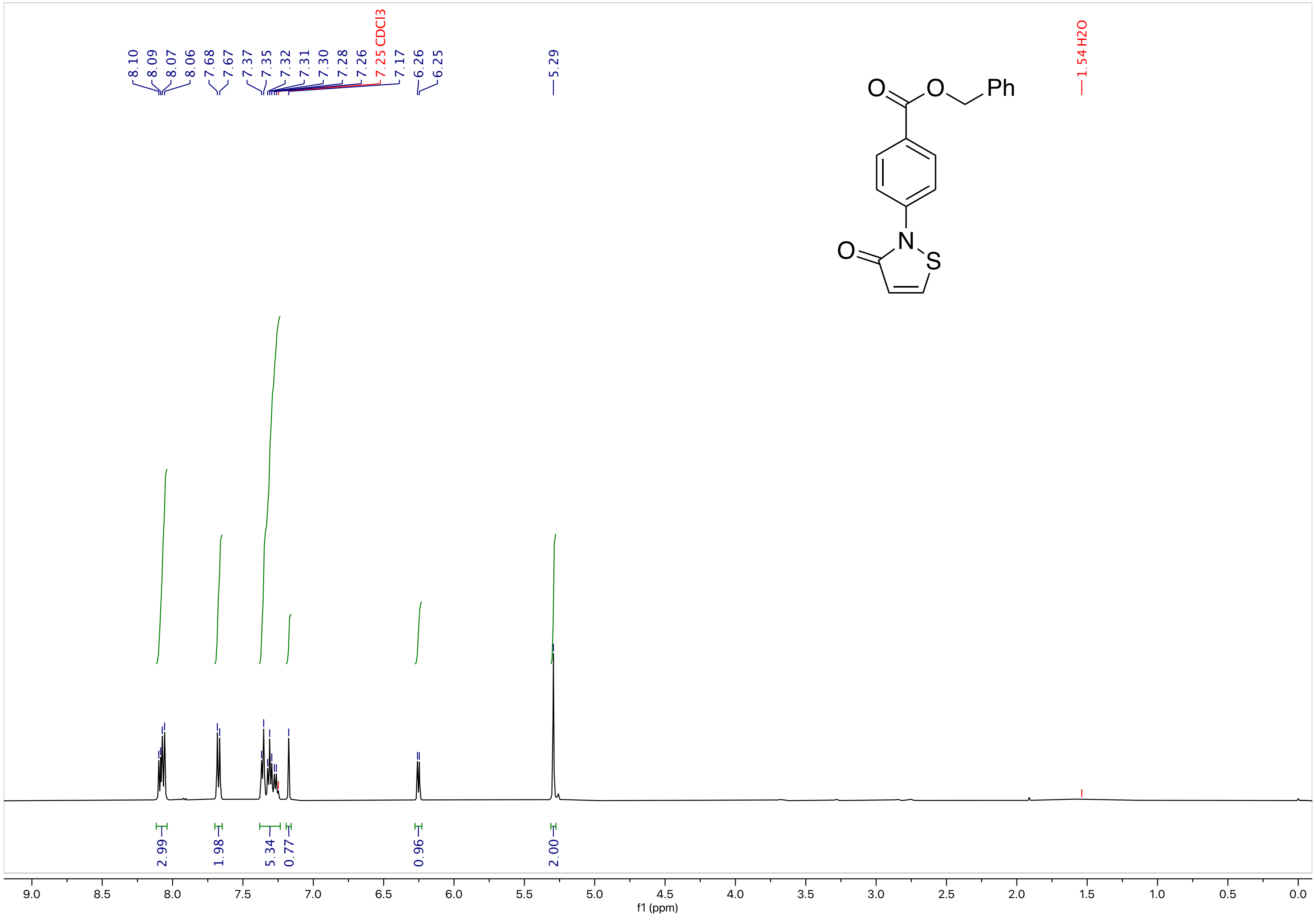

ISFP10 13C NMR

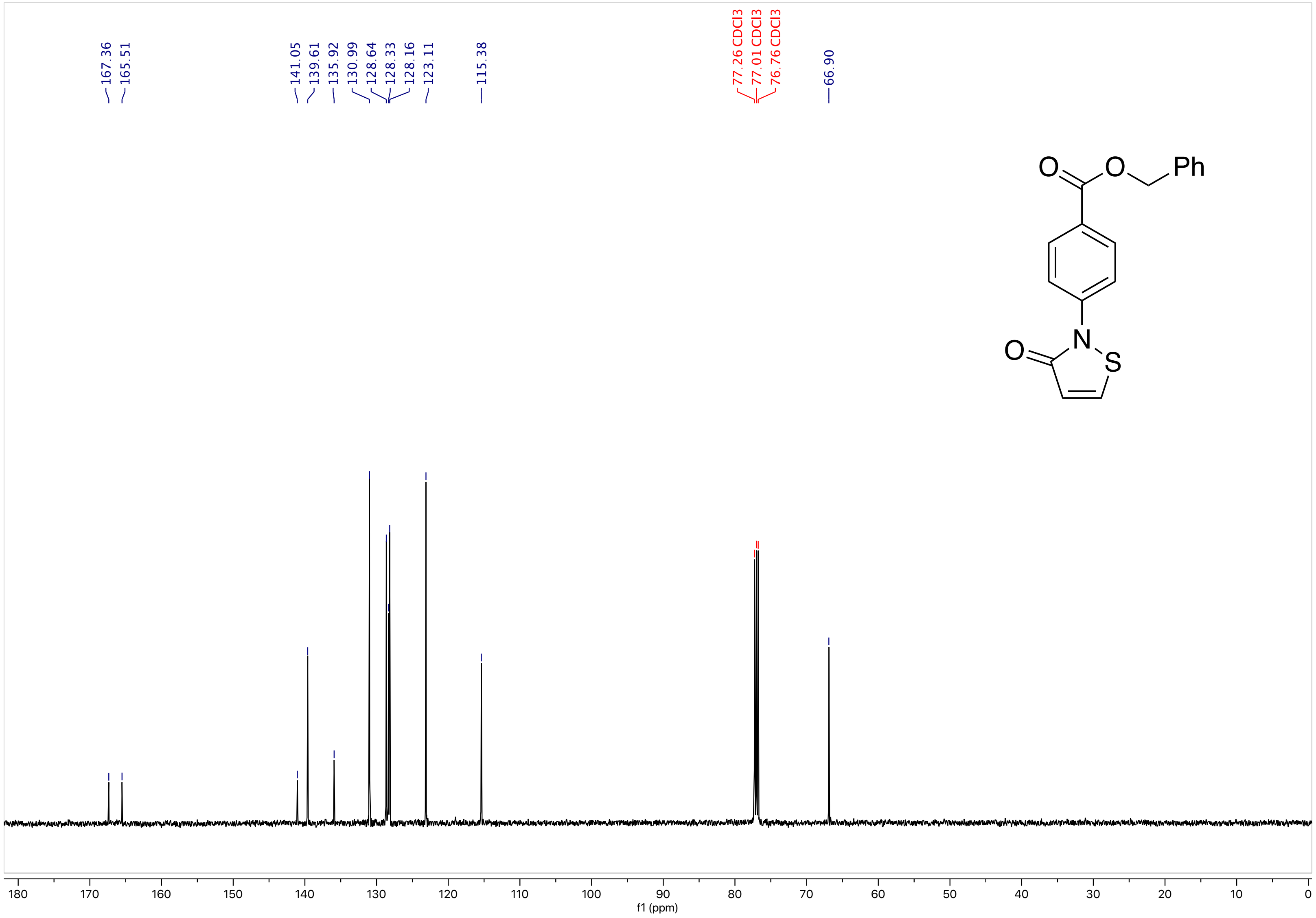

ISFP11 1H NMR

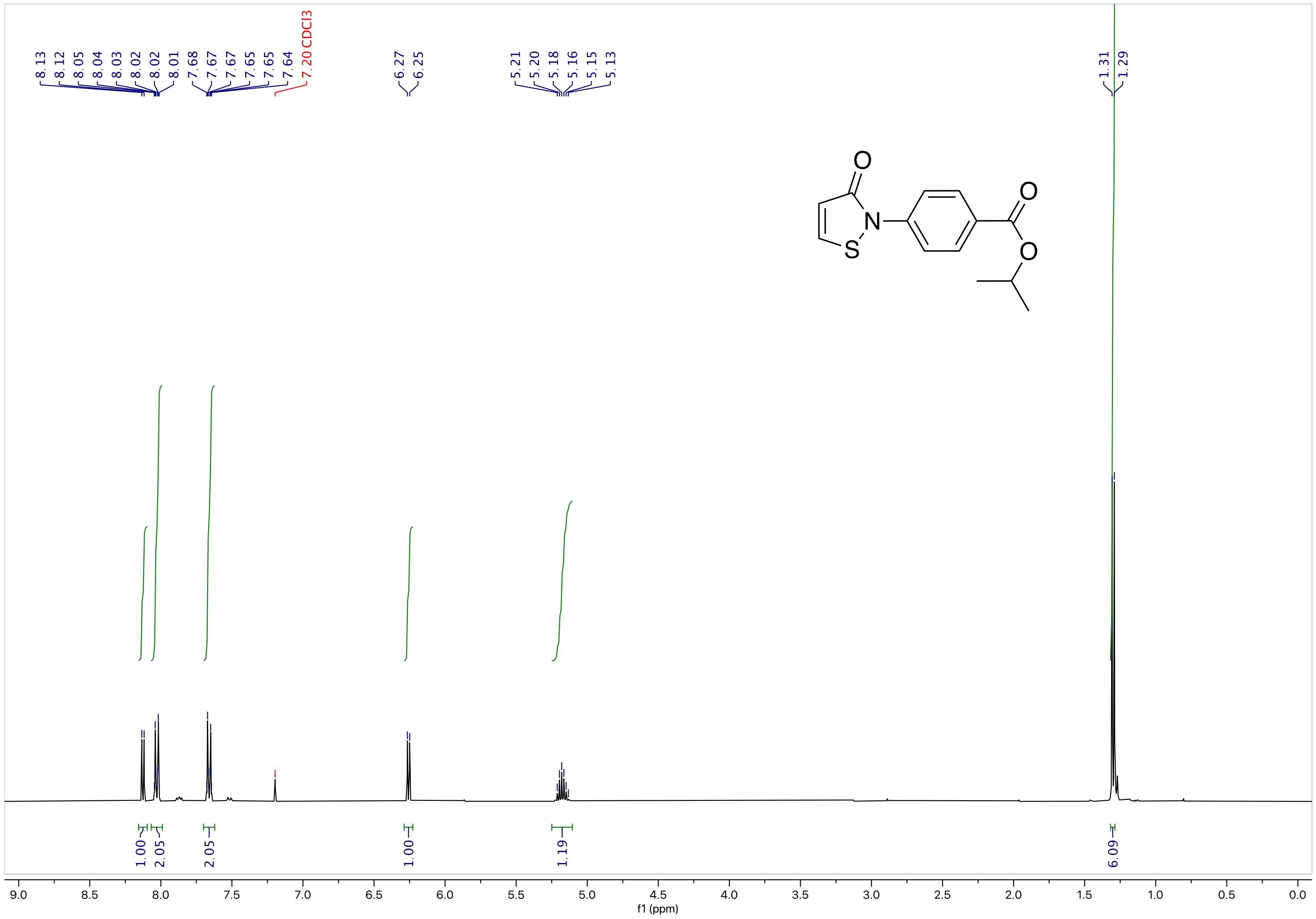

ISFP11 13C NMR

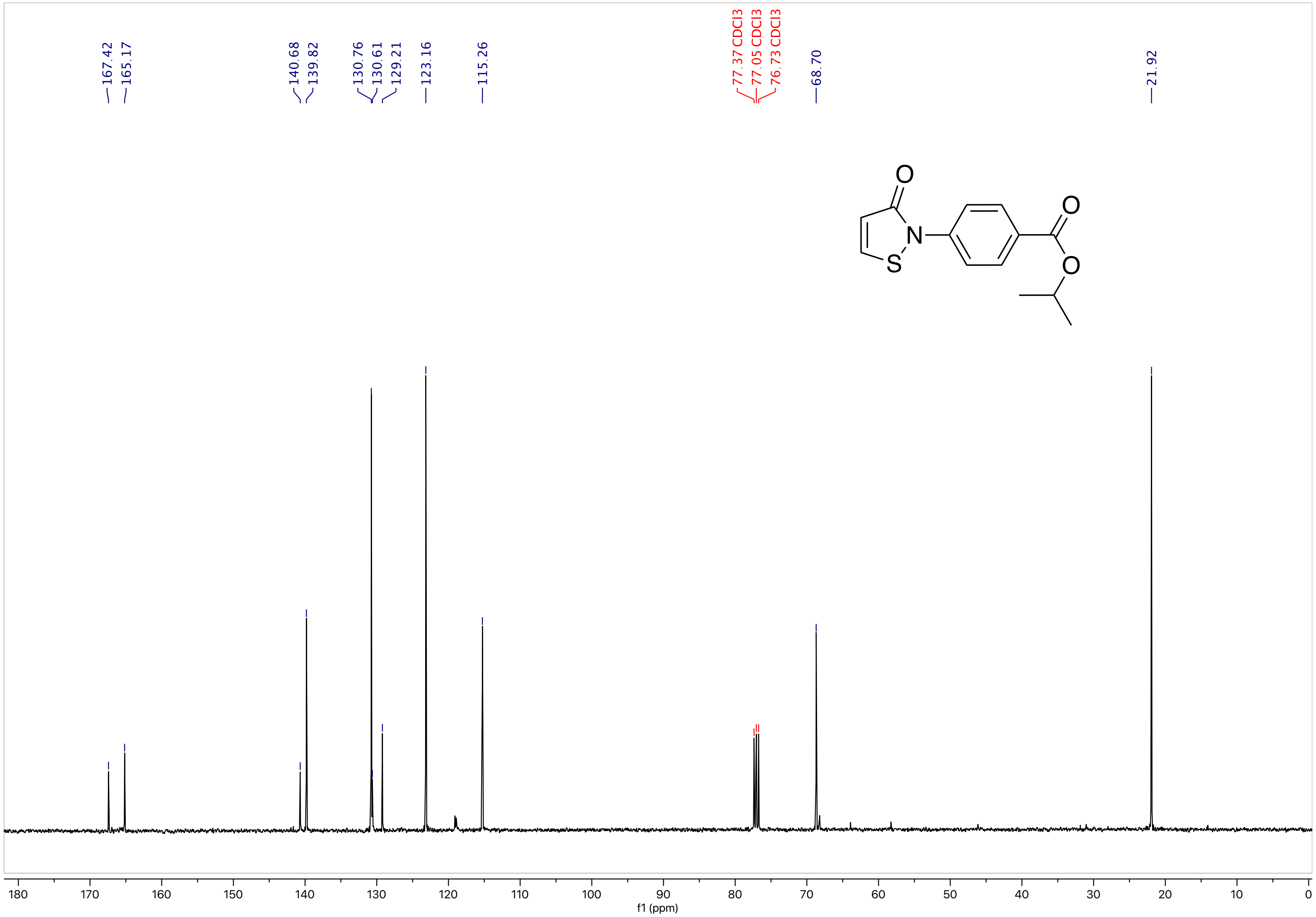

ISFP12 1H NMR

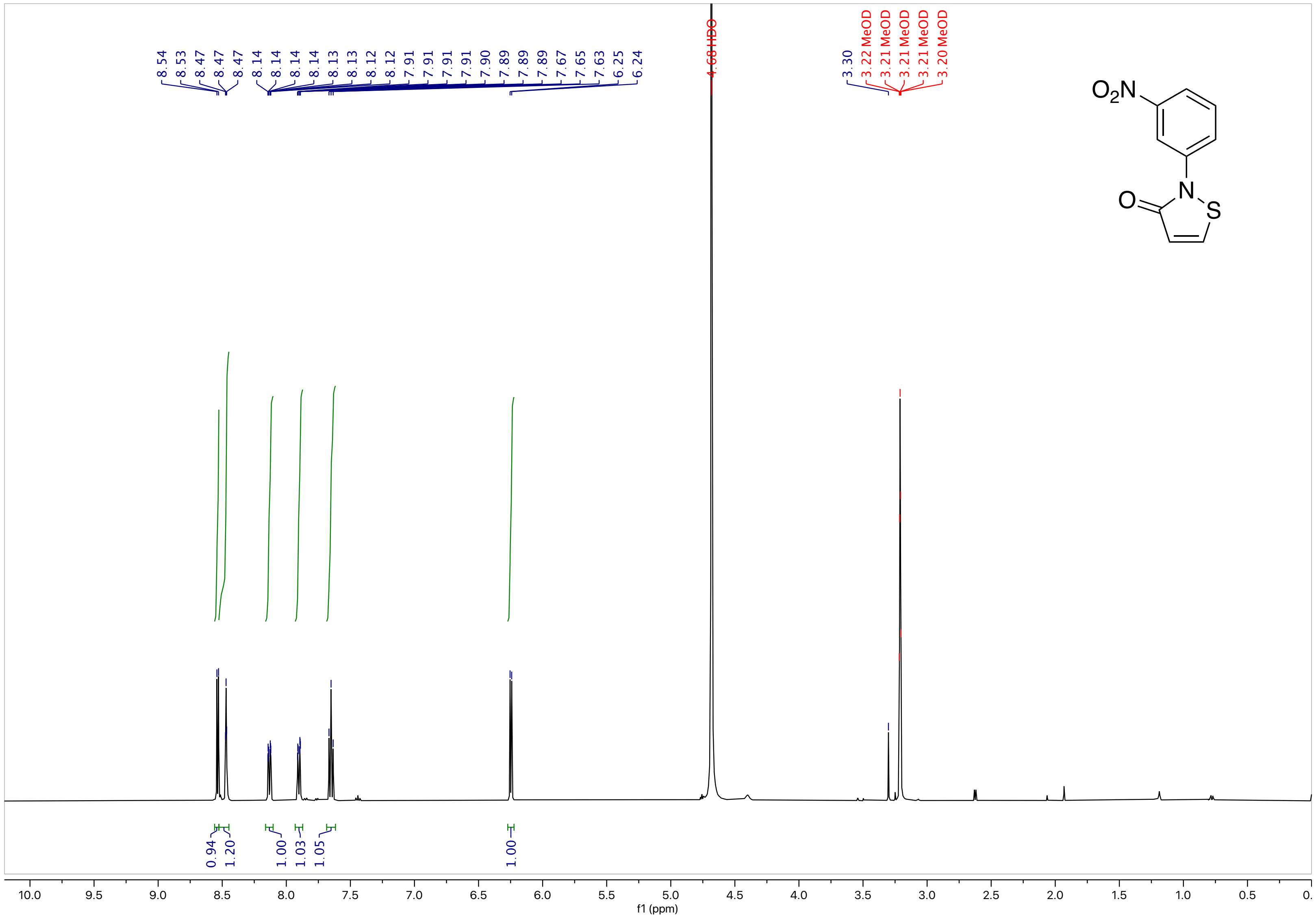

ISFP12 13C NMR

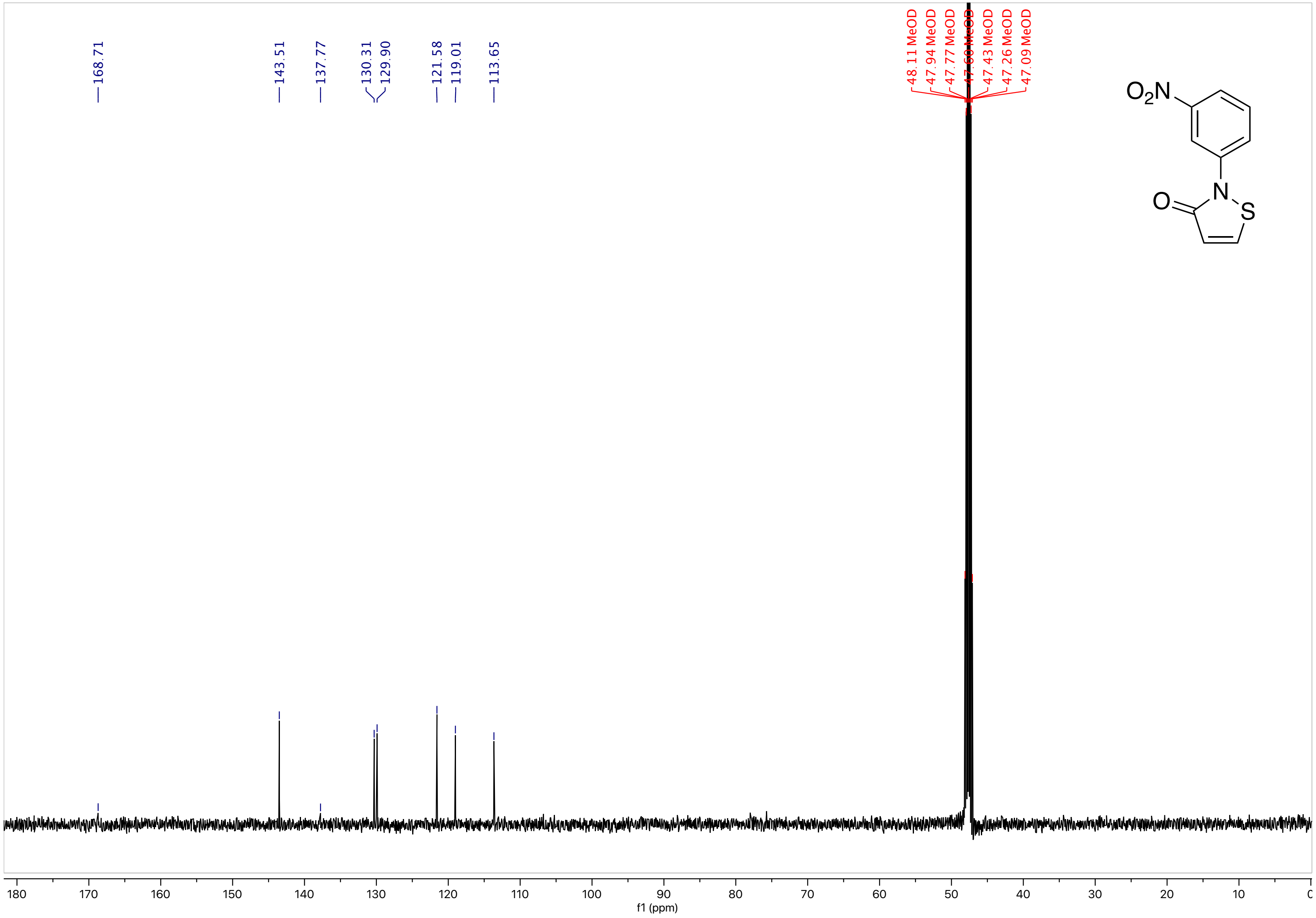

ISFP13 1H NMR

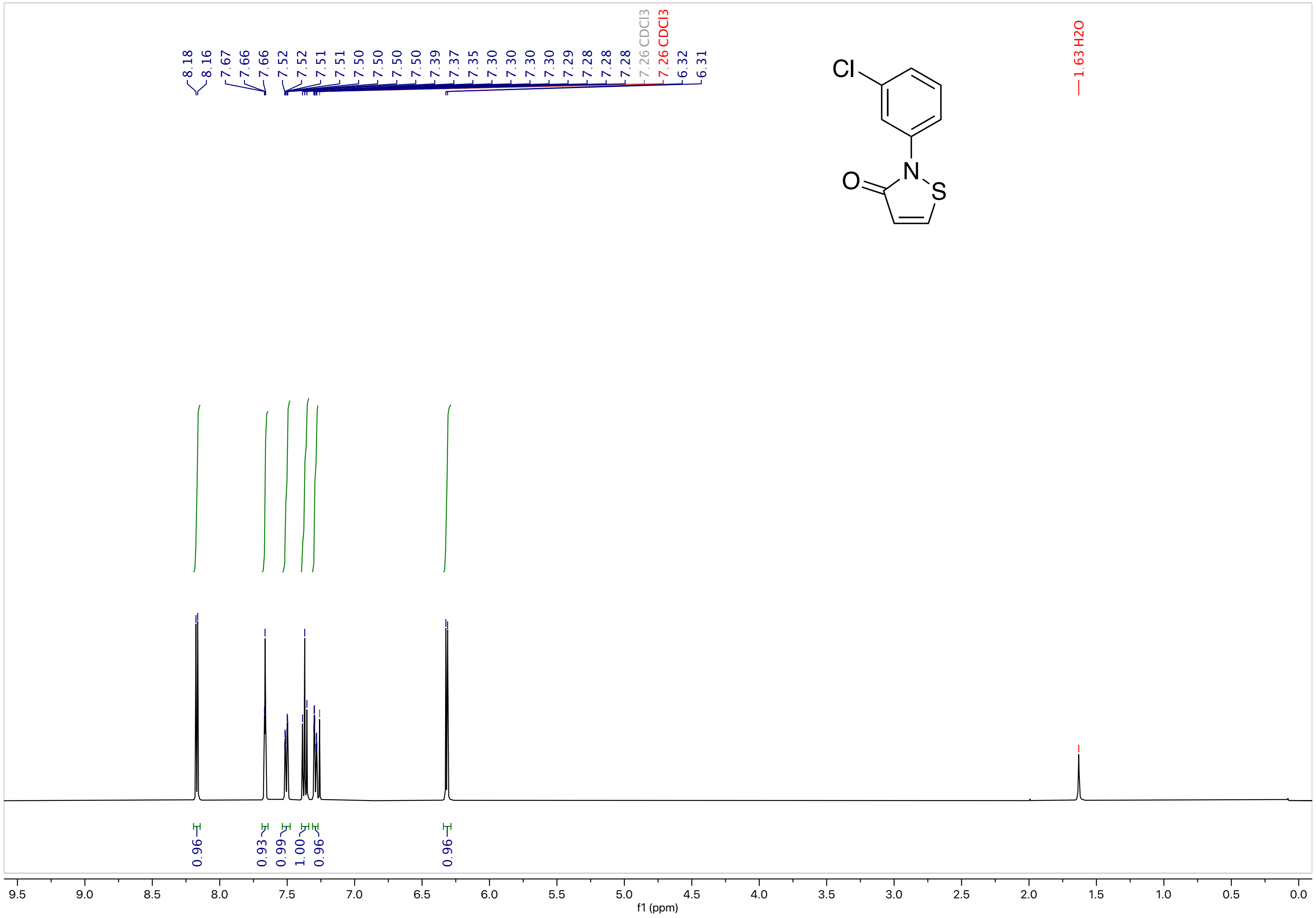

ISFP13 13C NMR

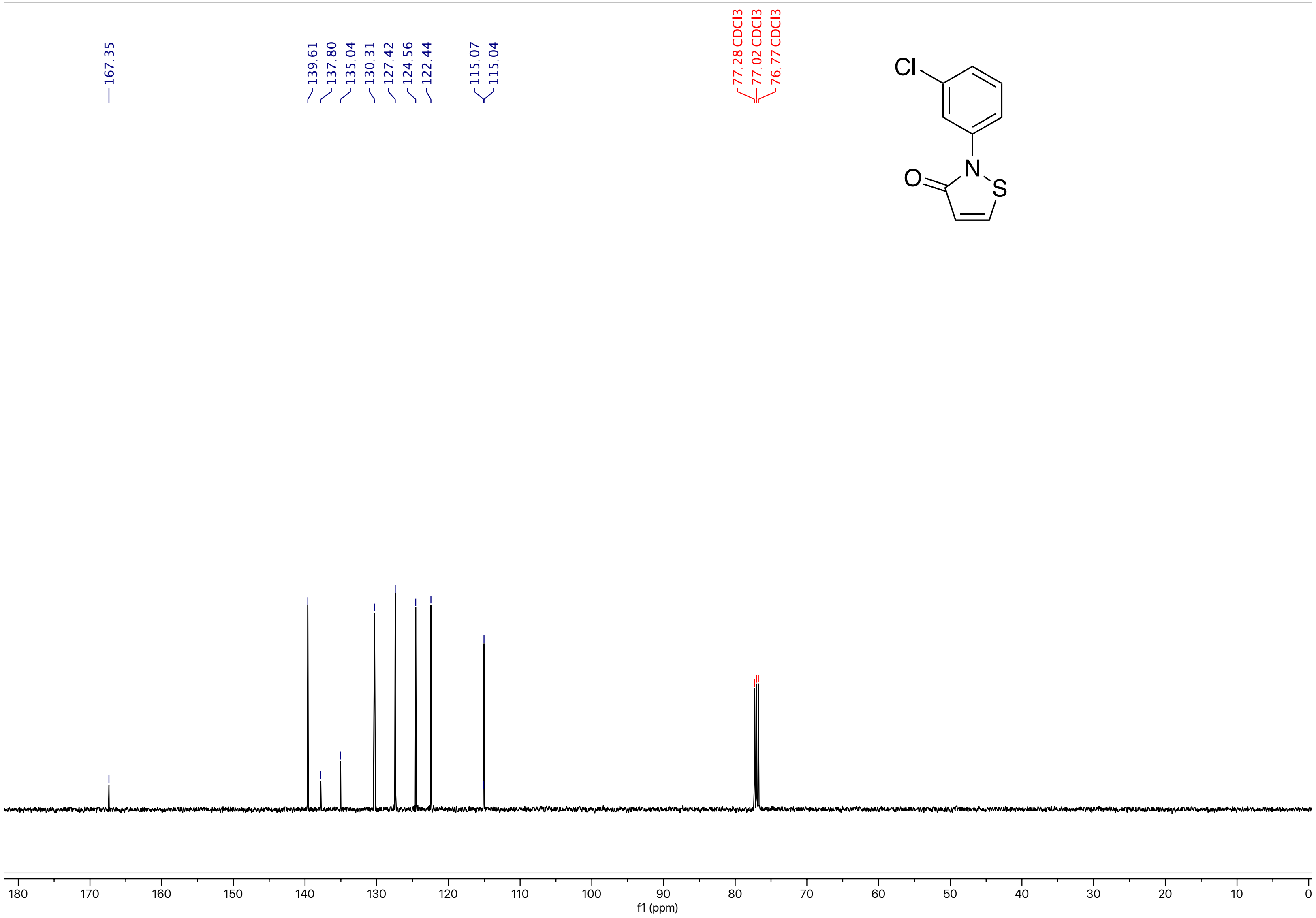

ISFP14 1H NMR

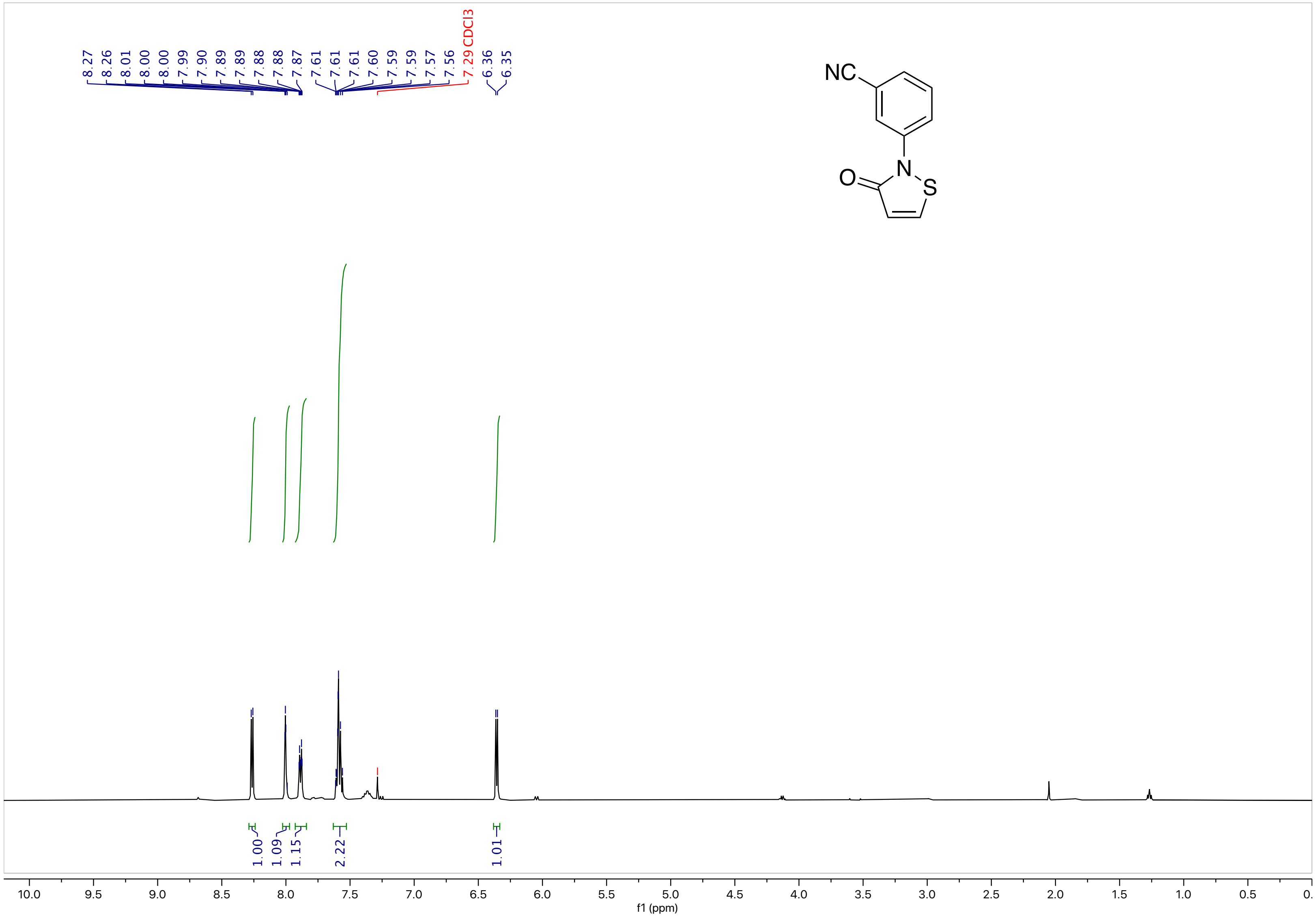

ISFP14 13C NMR

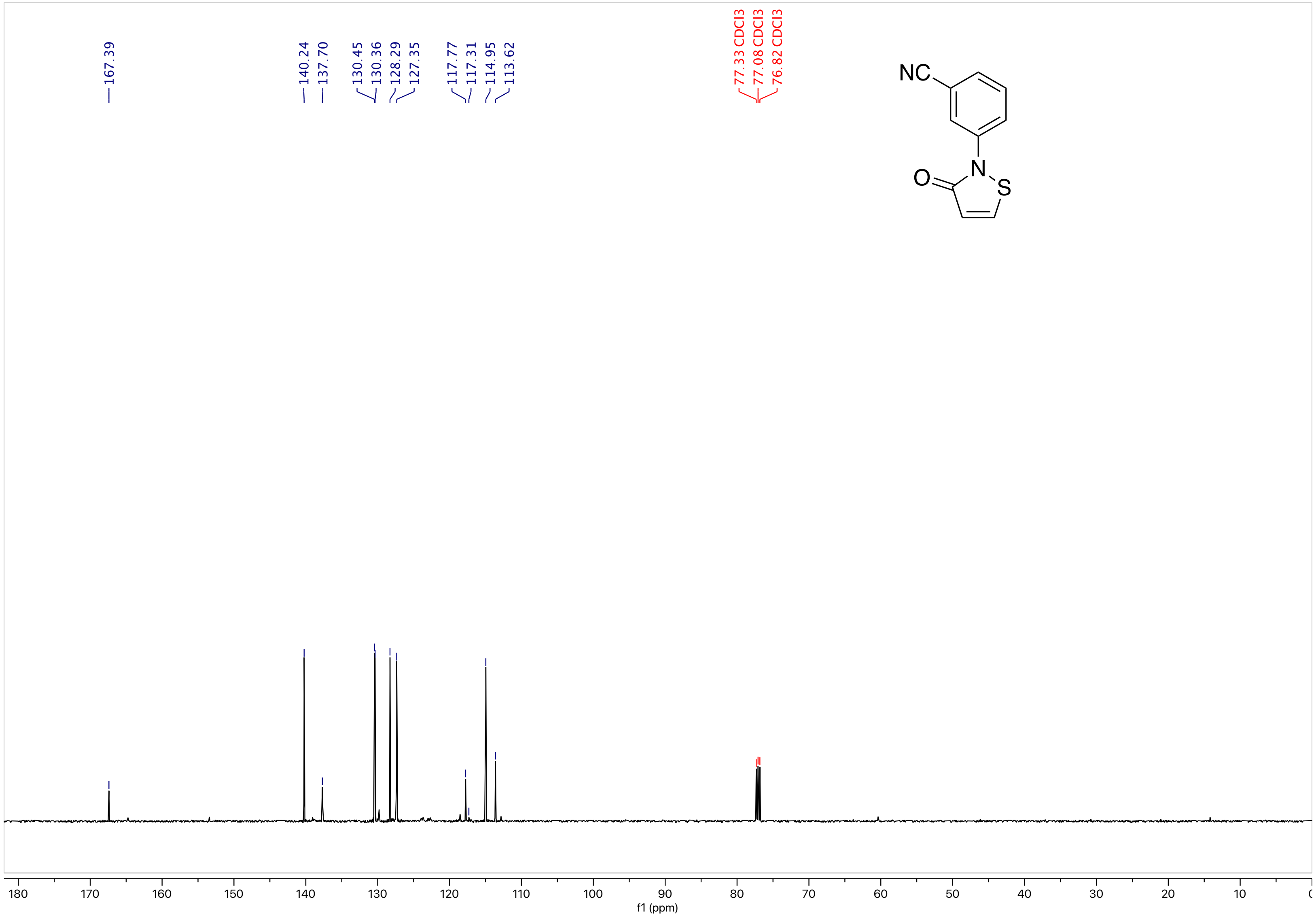

ISFP15 1H NMR

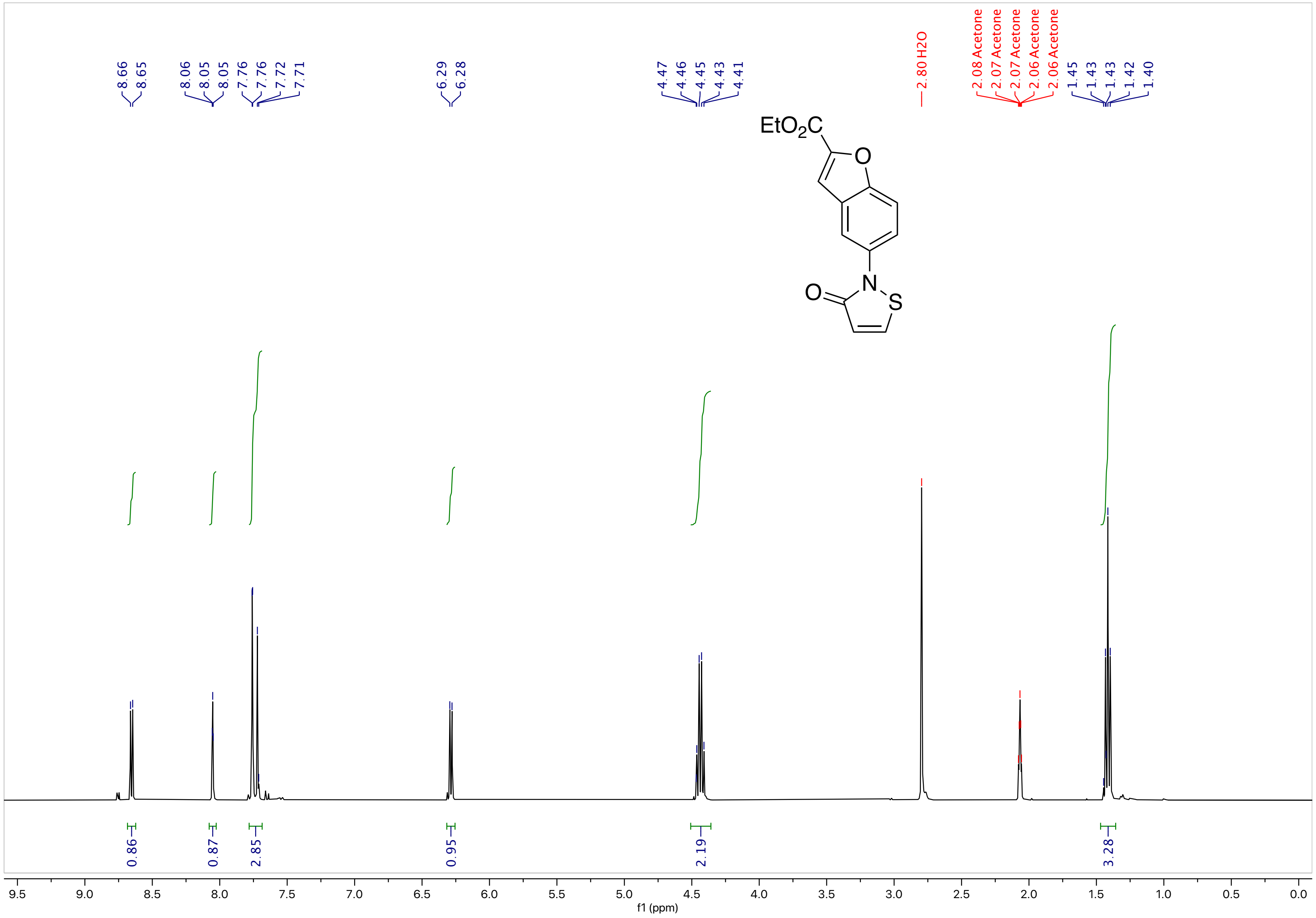

ISFP15 13C NMR

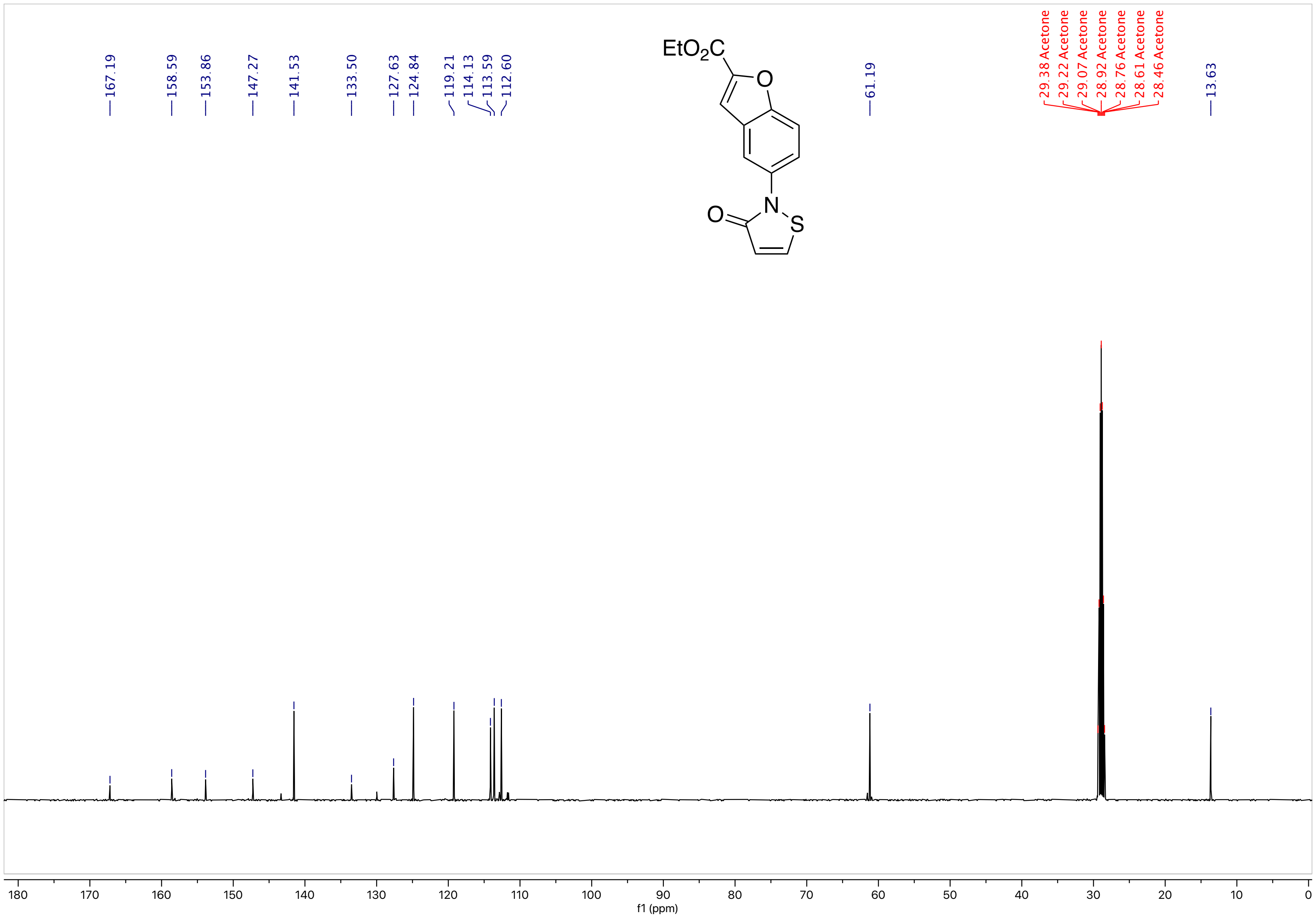

ISFP16 1H NMR

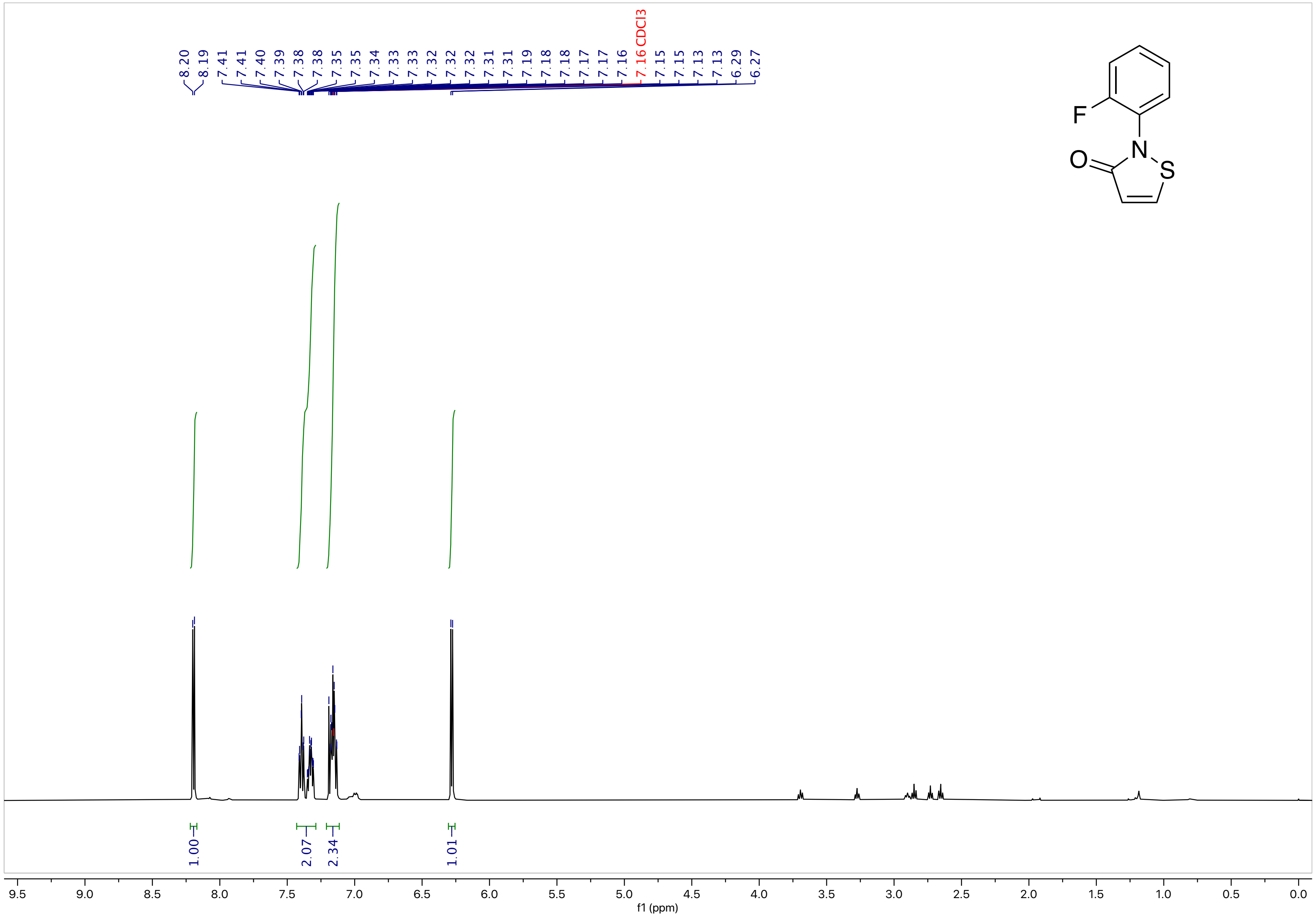

ISFP16 13C NMR

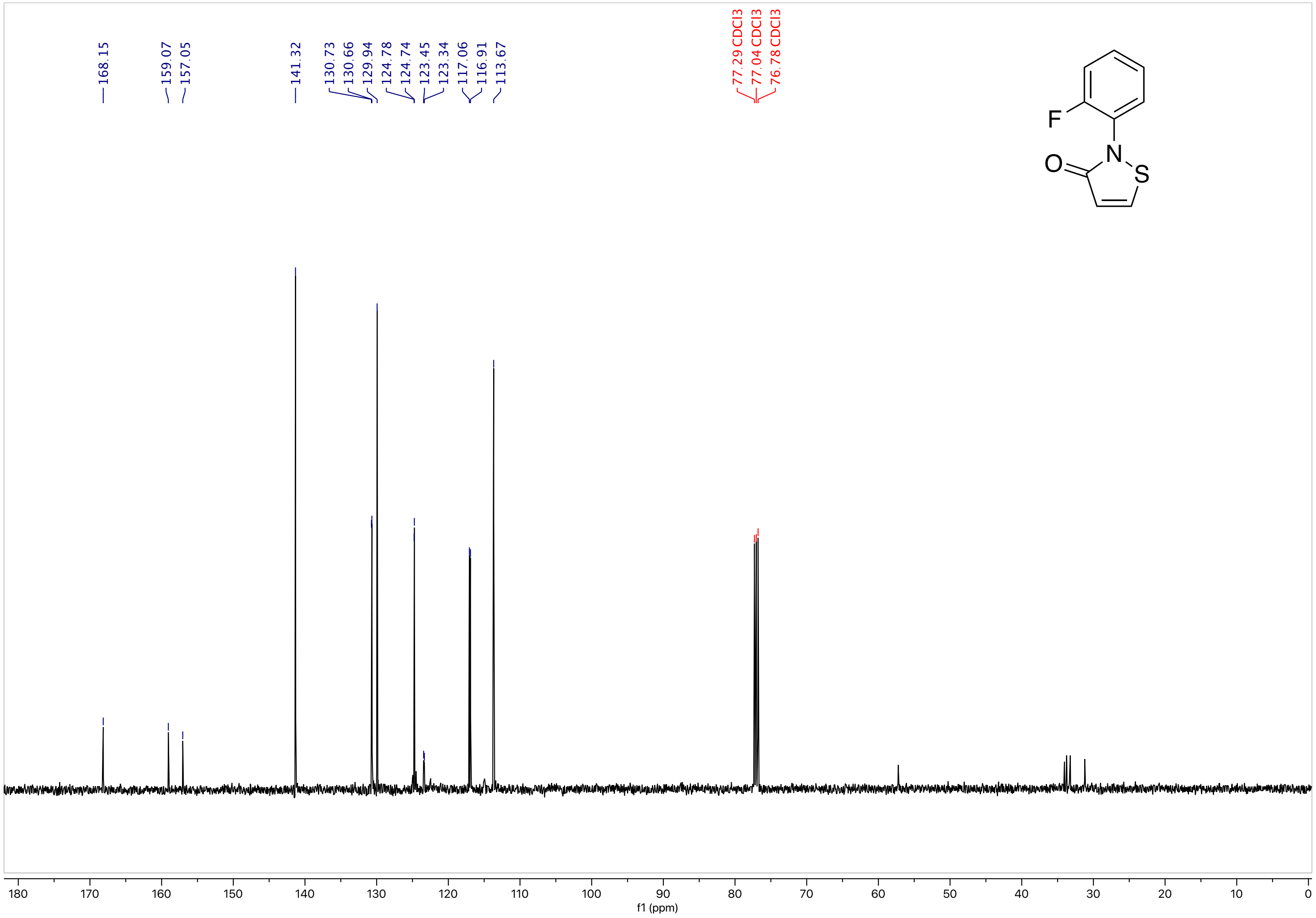

ISFP16 19F NMR

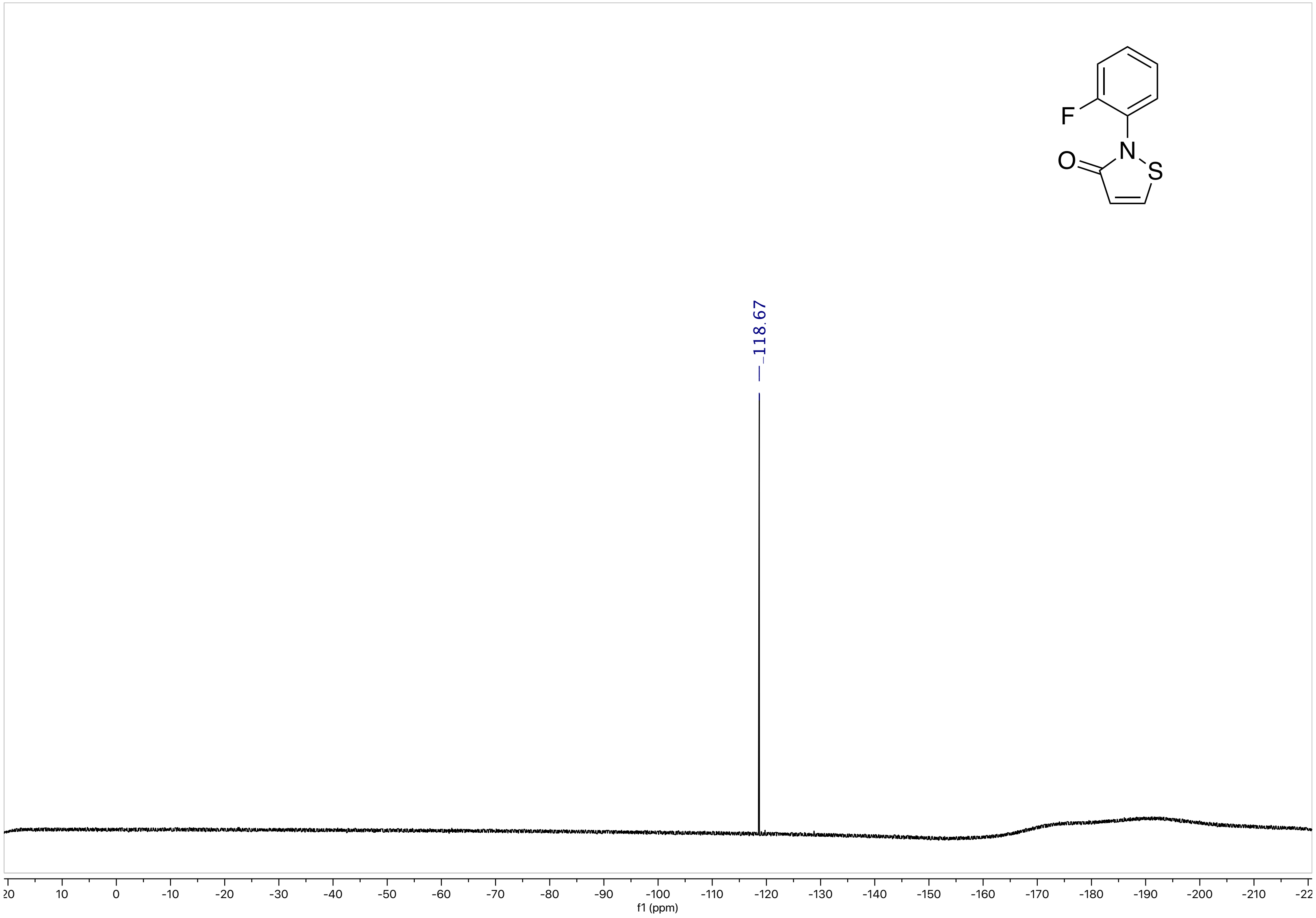

ISFP17 1H NMR

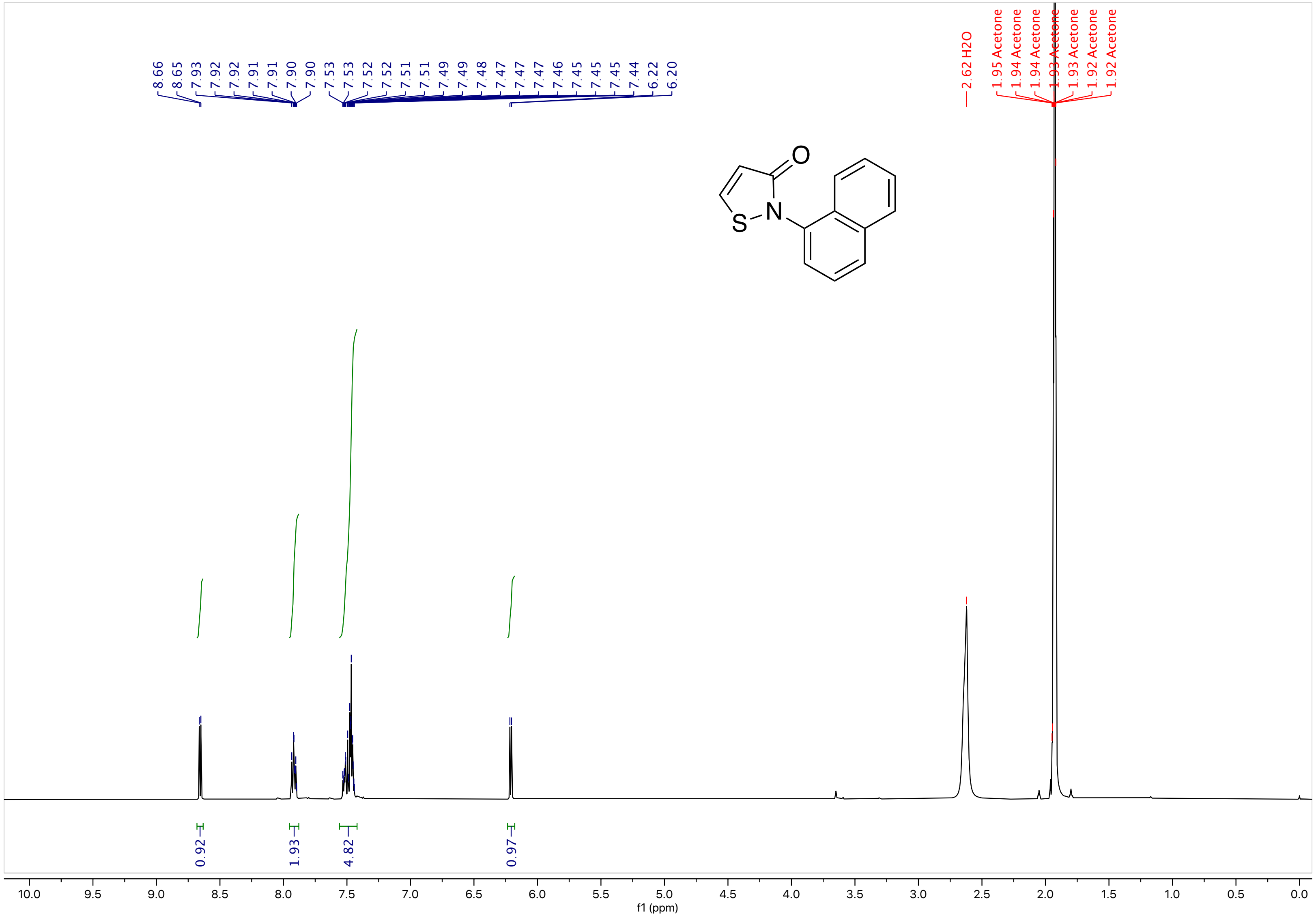

ISFP17 13C NMR

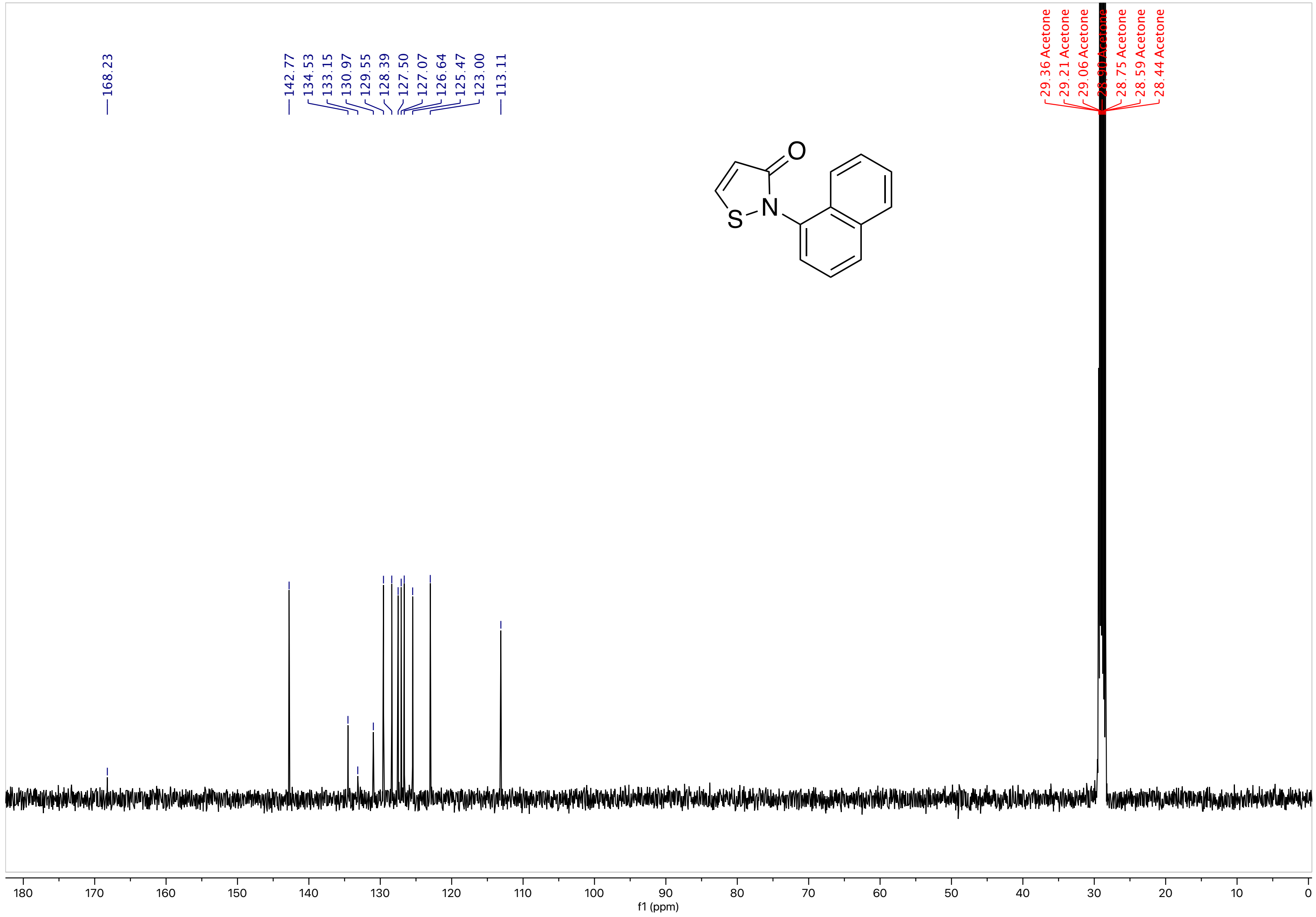

ISFP18 1H NMR

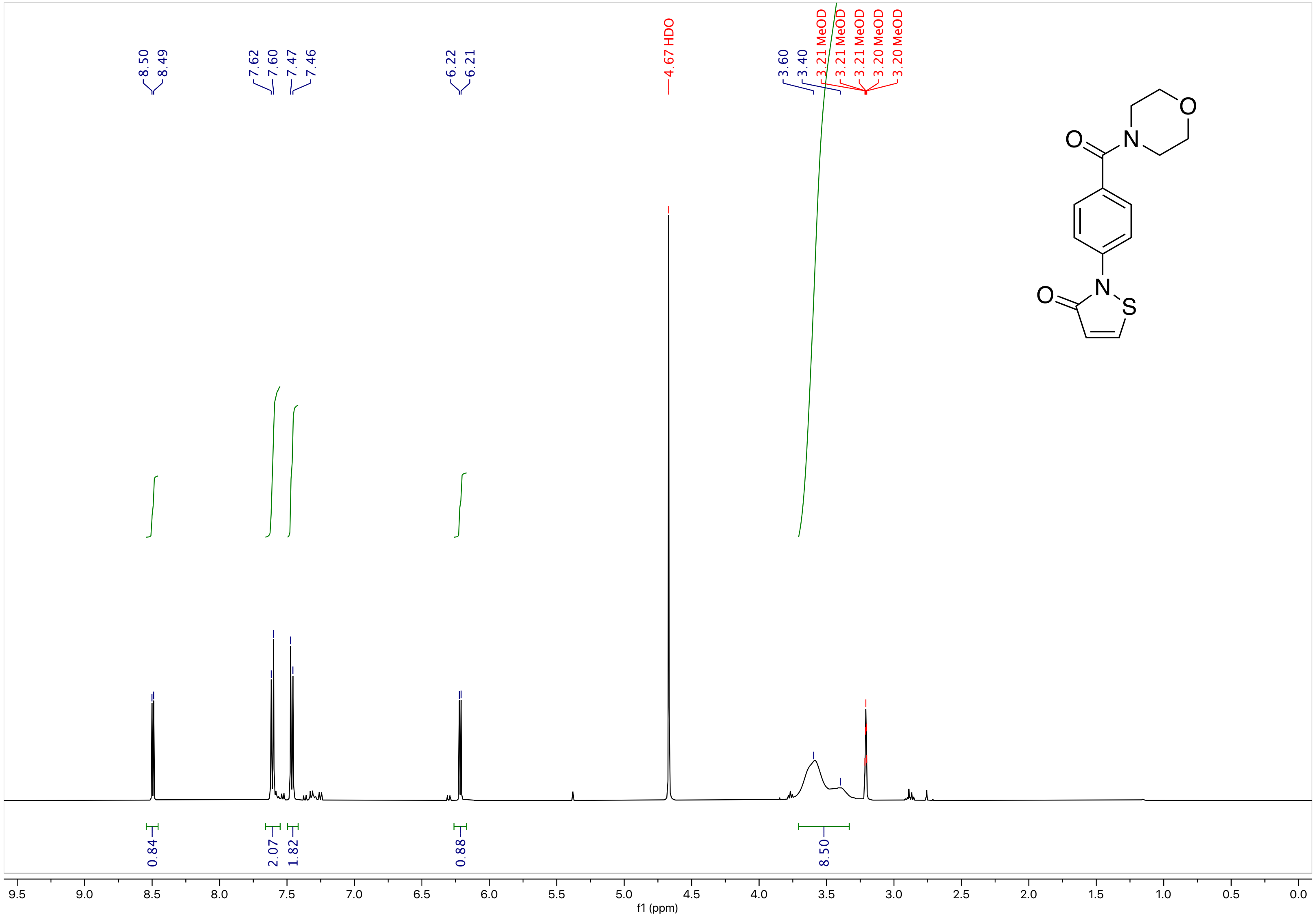

ISFP18 13C NMR

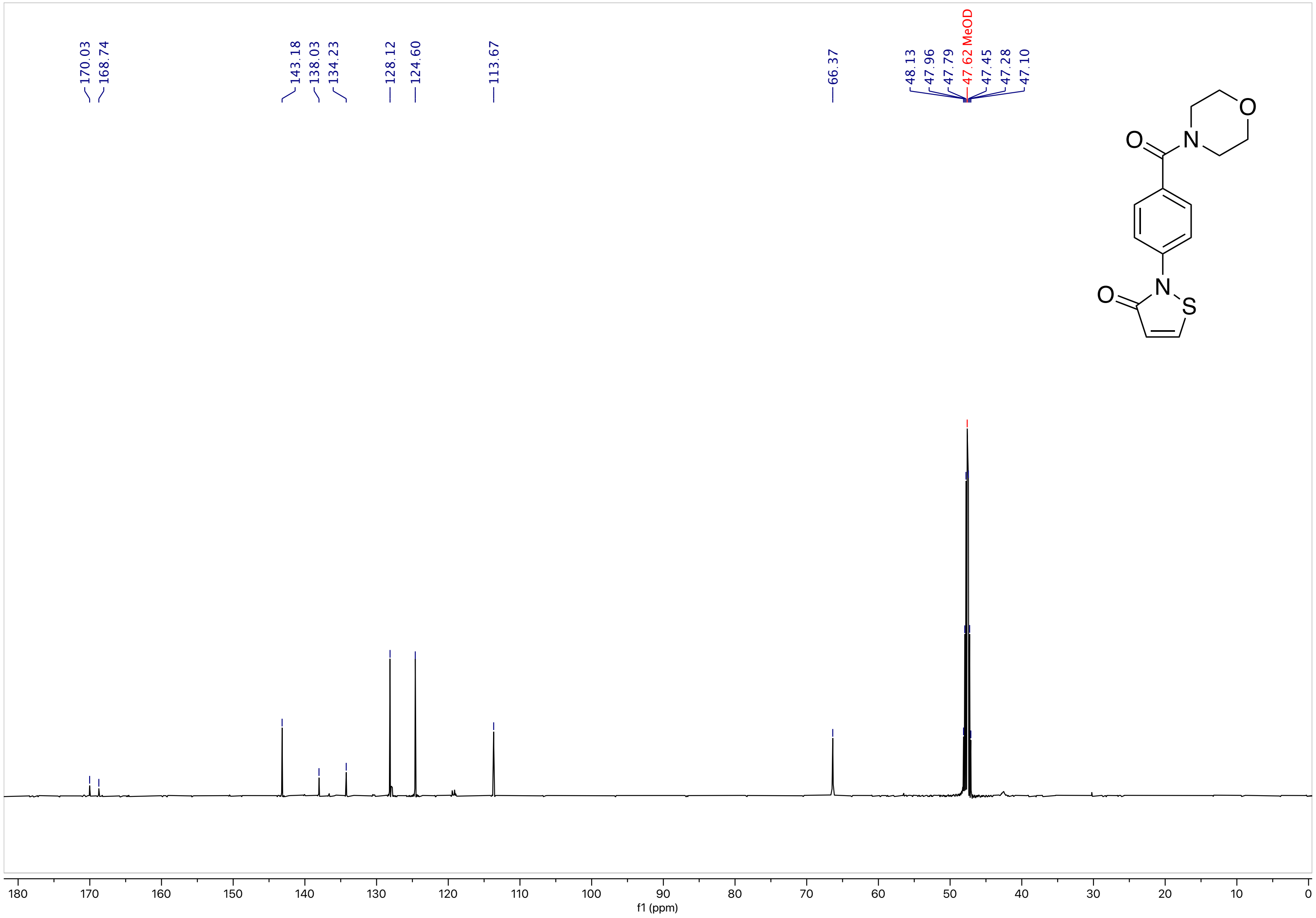

ISFP19 1H NMR

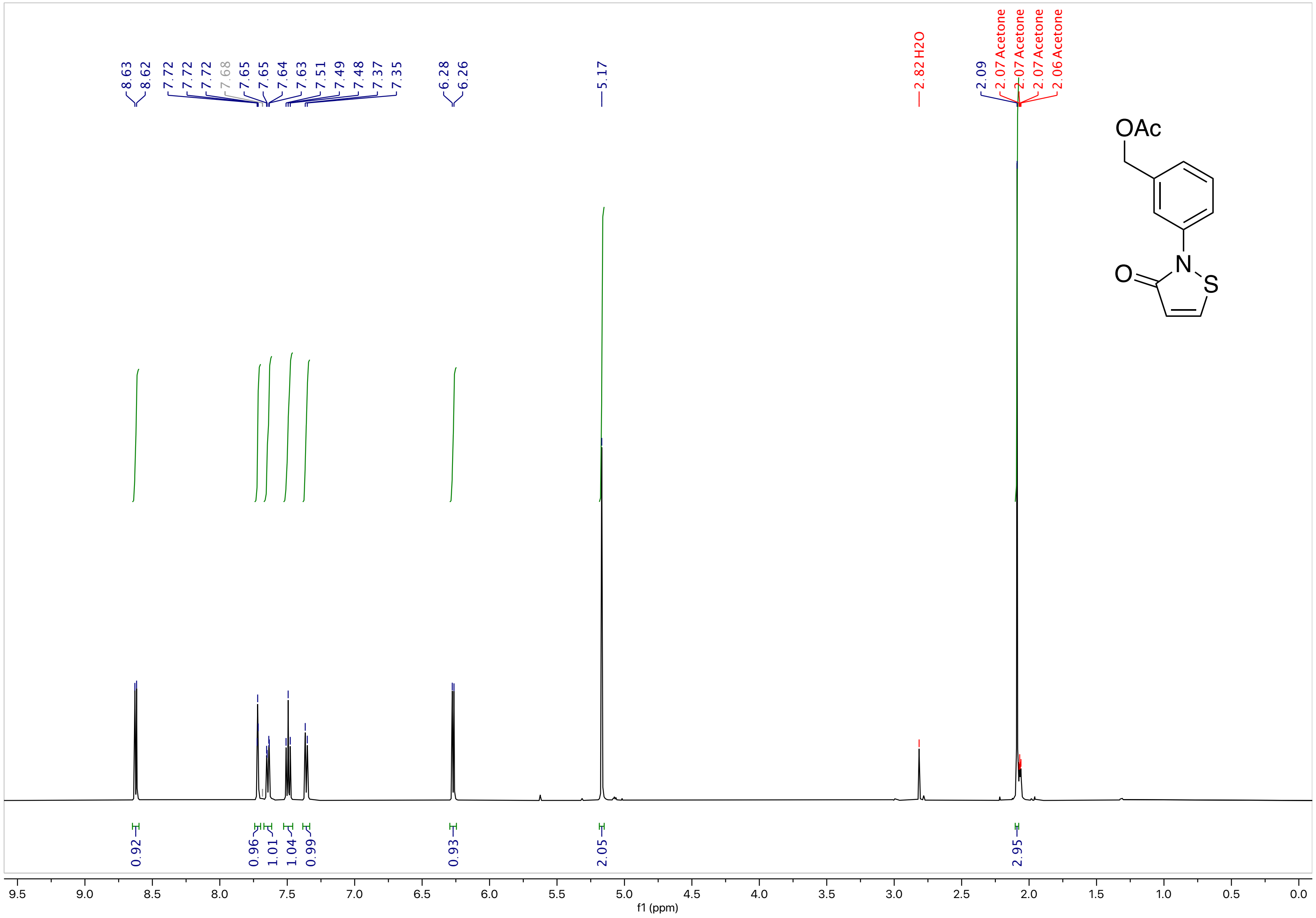

ISFP19 13C NMR

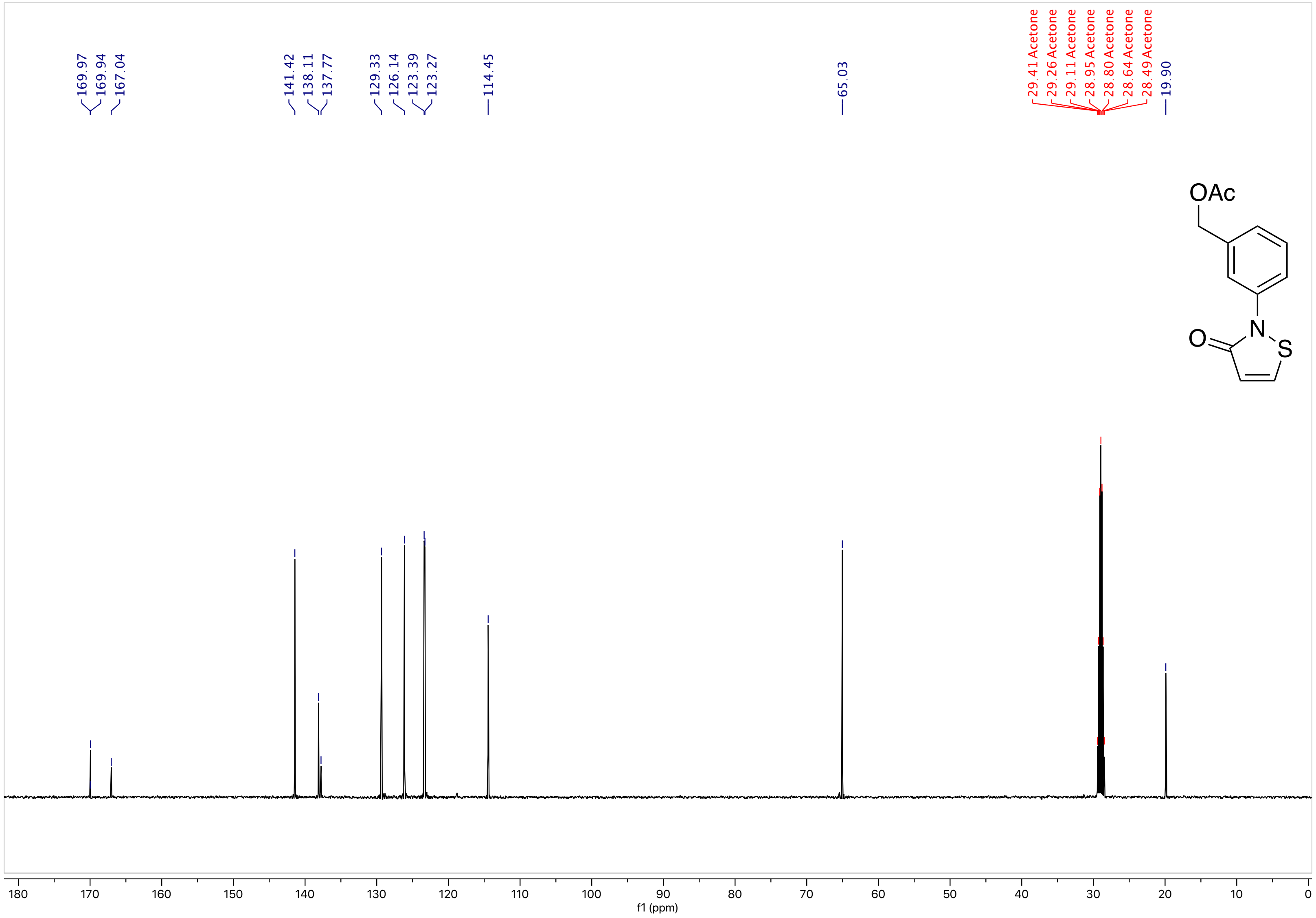

ISFP20 1H NMR

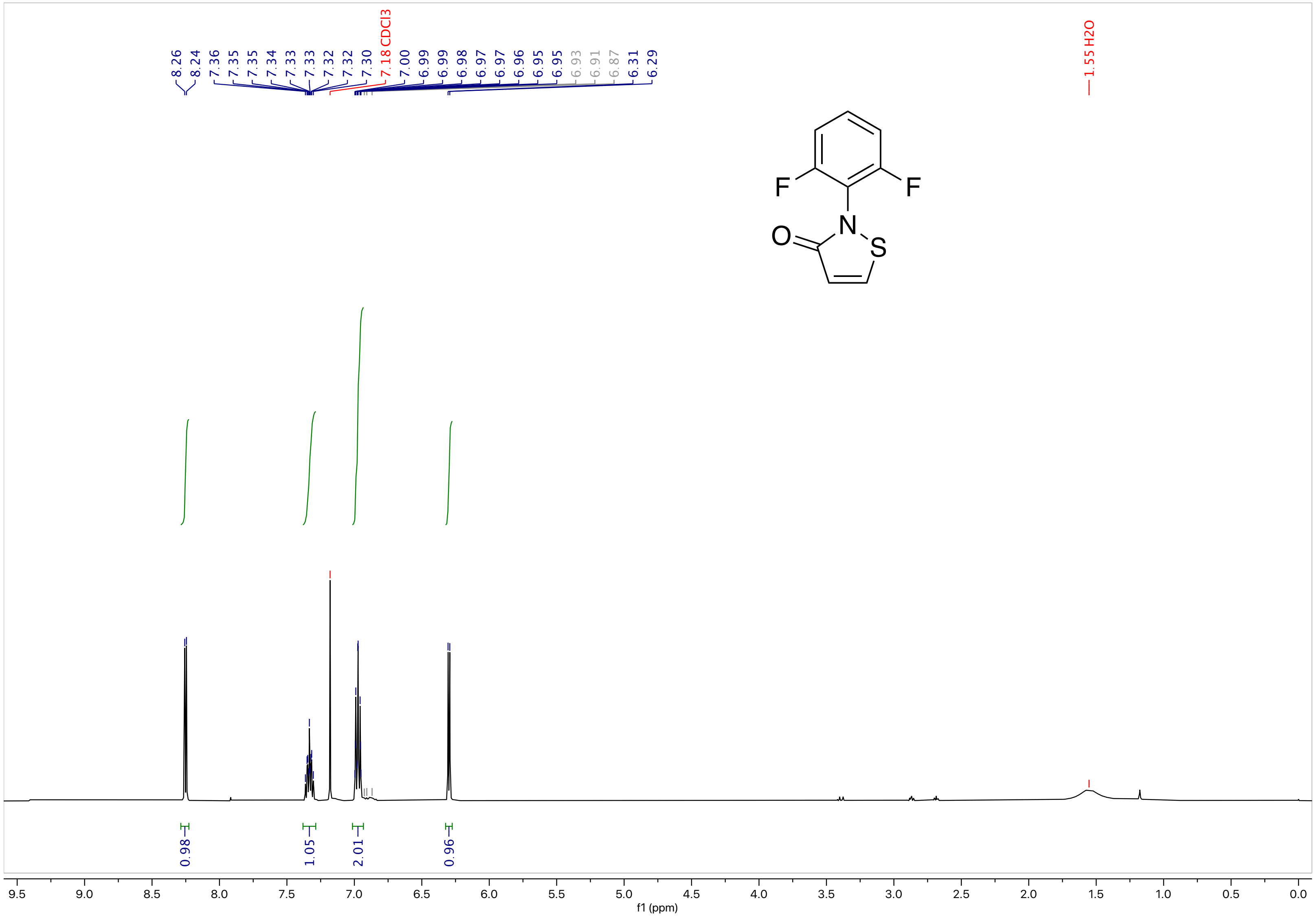

ISFP20 13C NMR

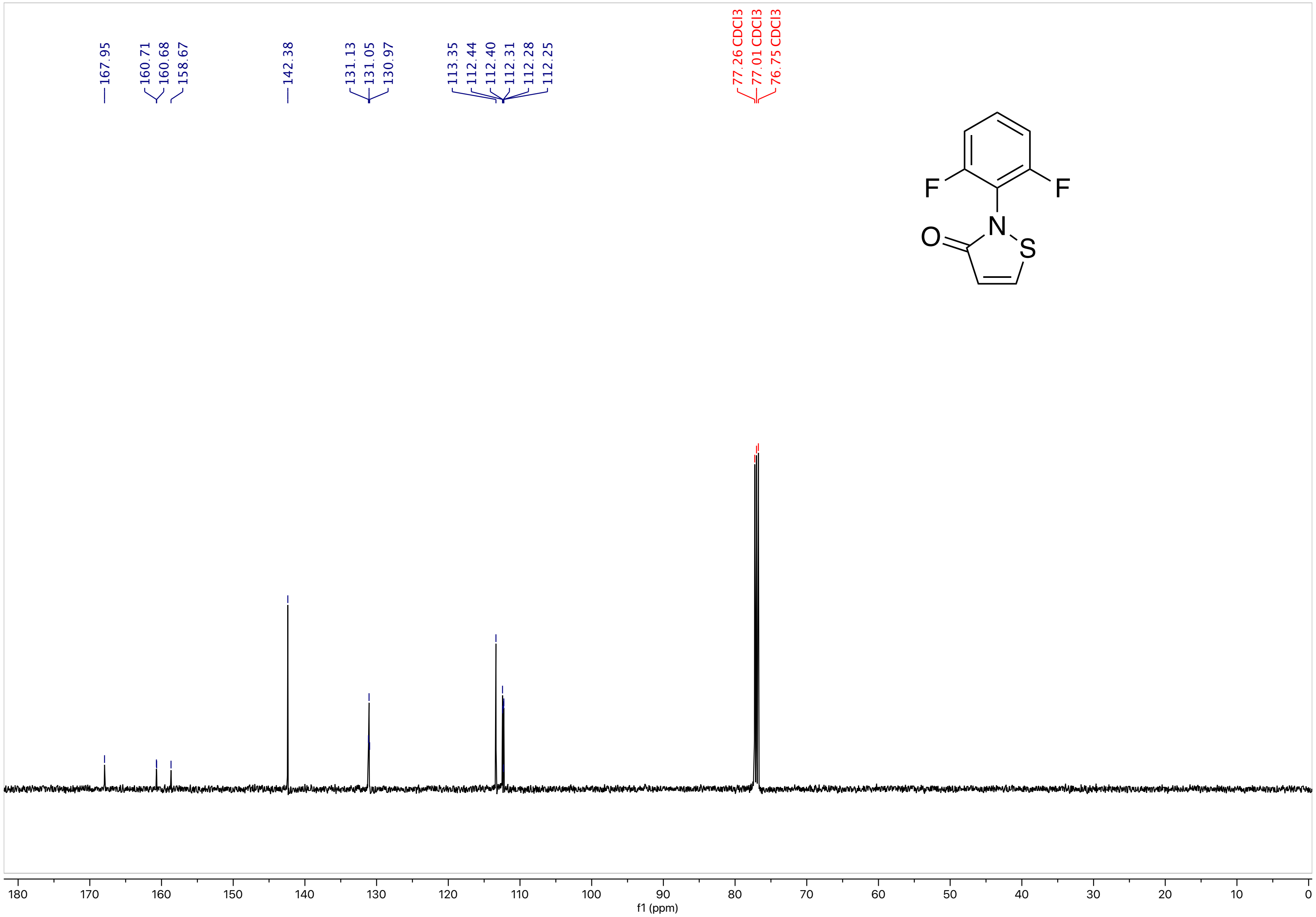

ISFP20 19F NMR

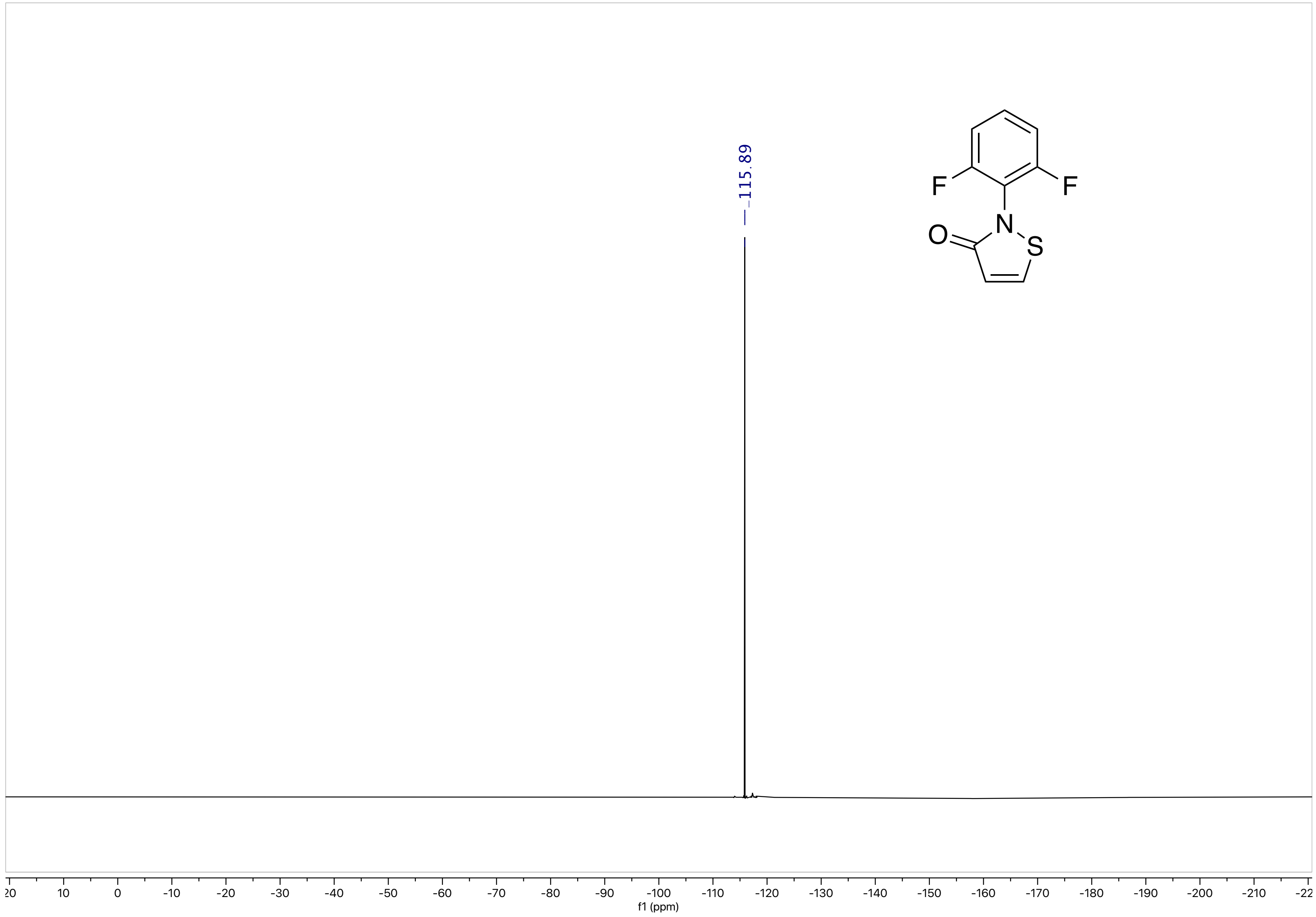

ISFP21 1H NMR

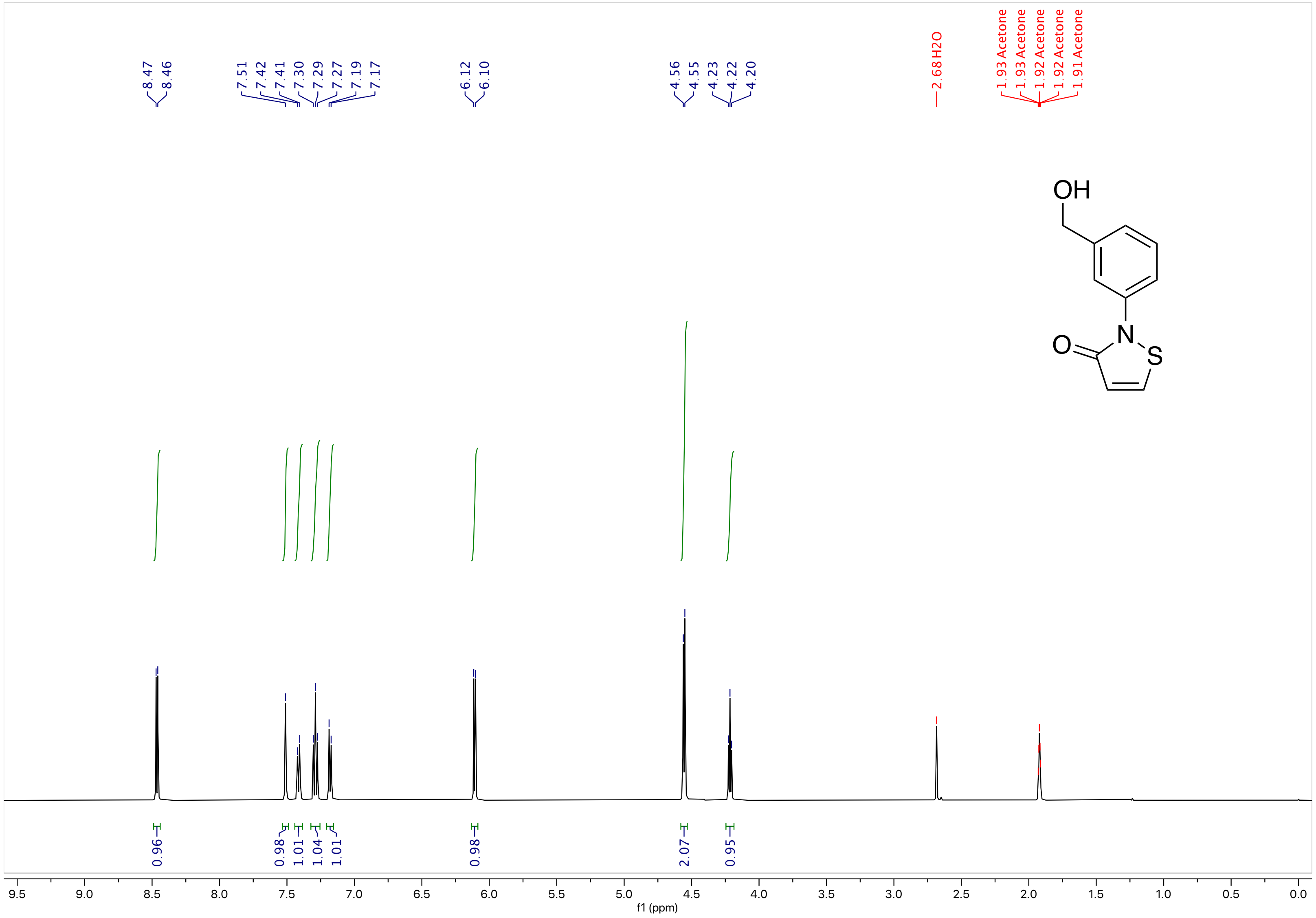

ISFP21 13C NMR

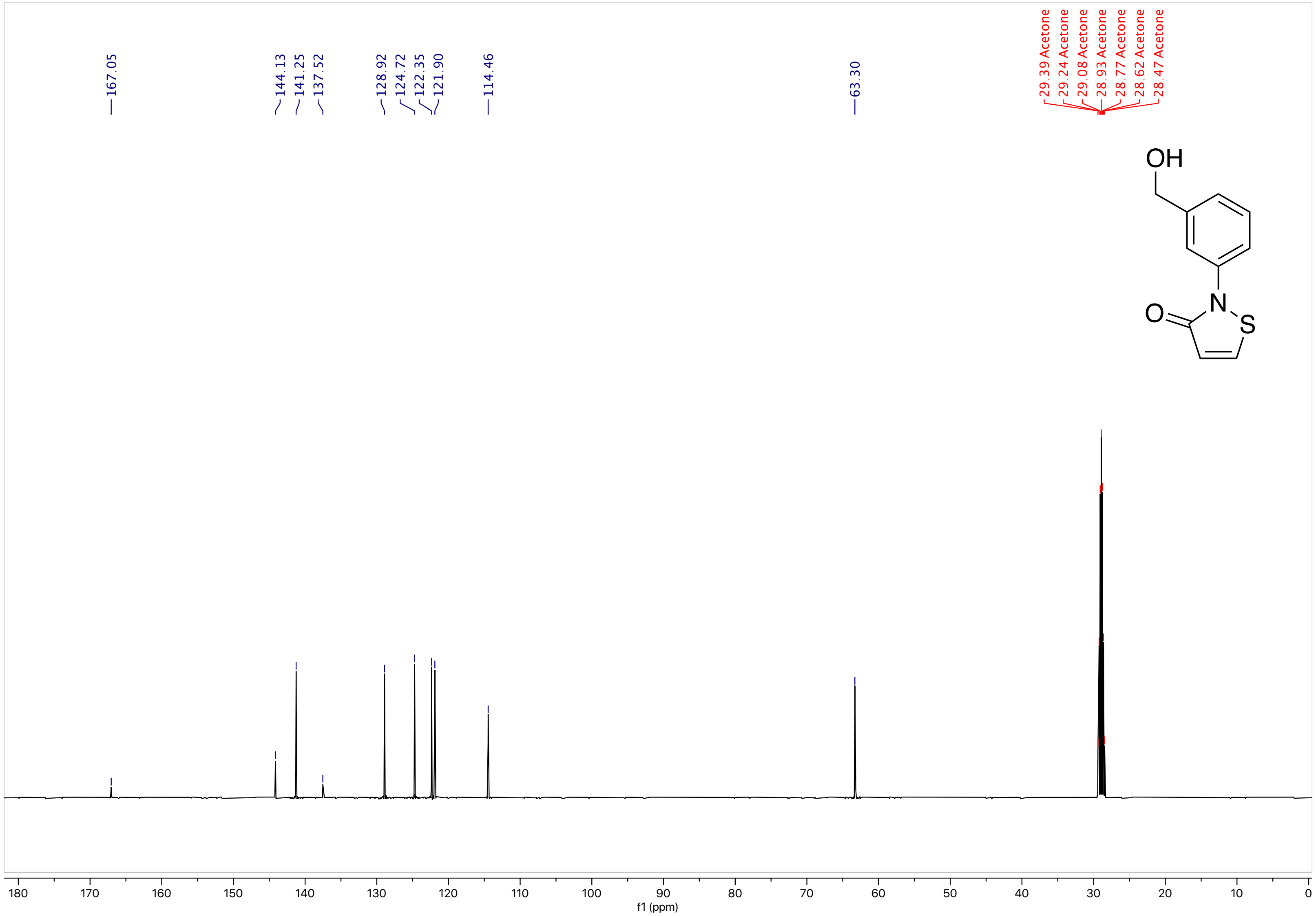

ISFP22 1H NMR

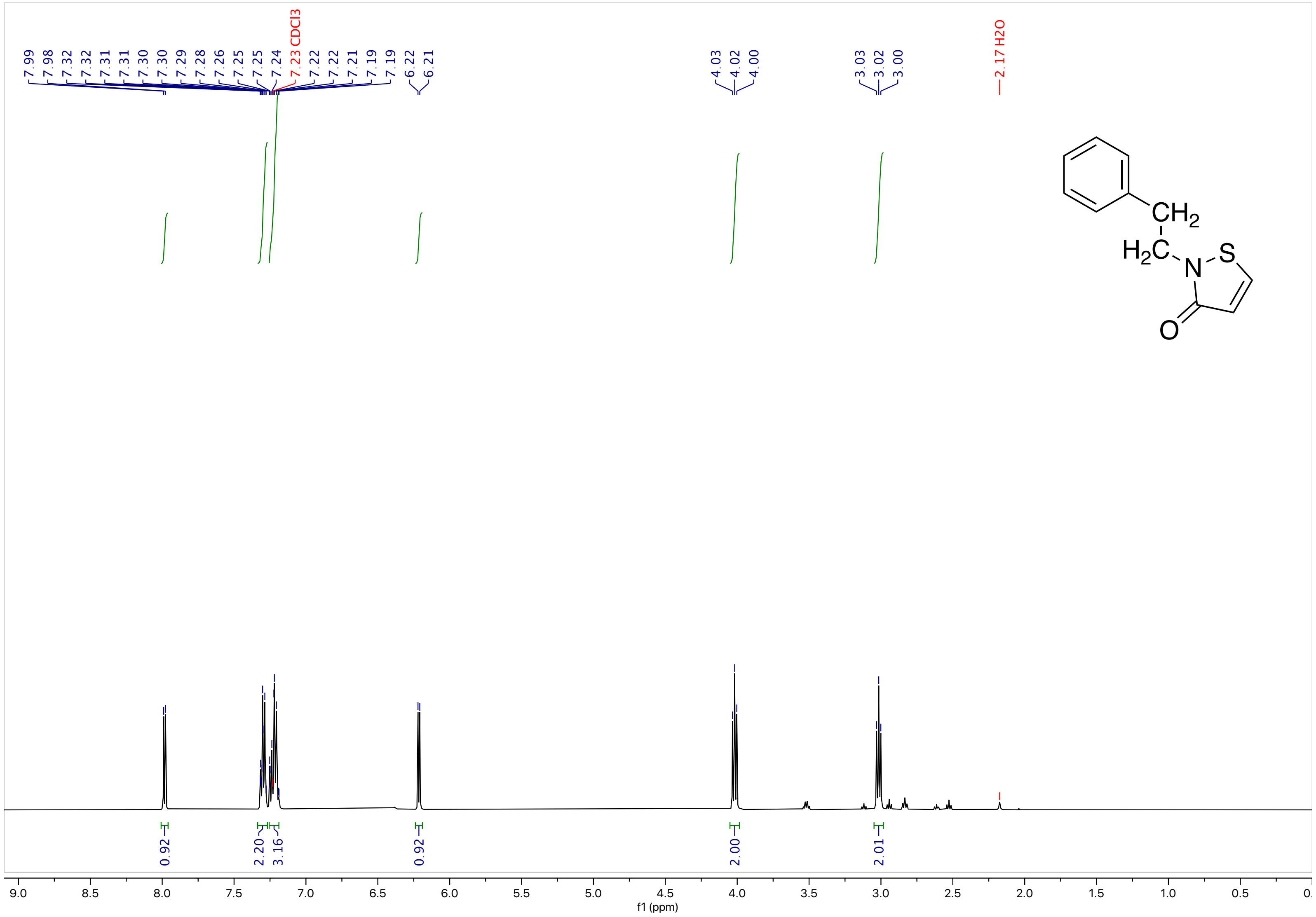

ISFP22 13C NMR

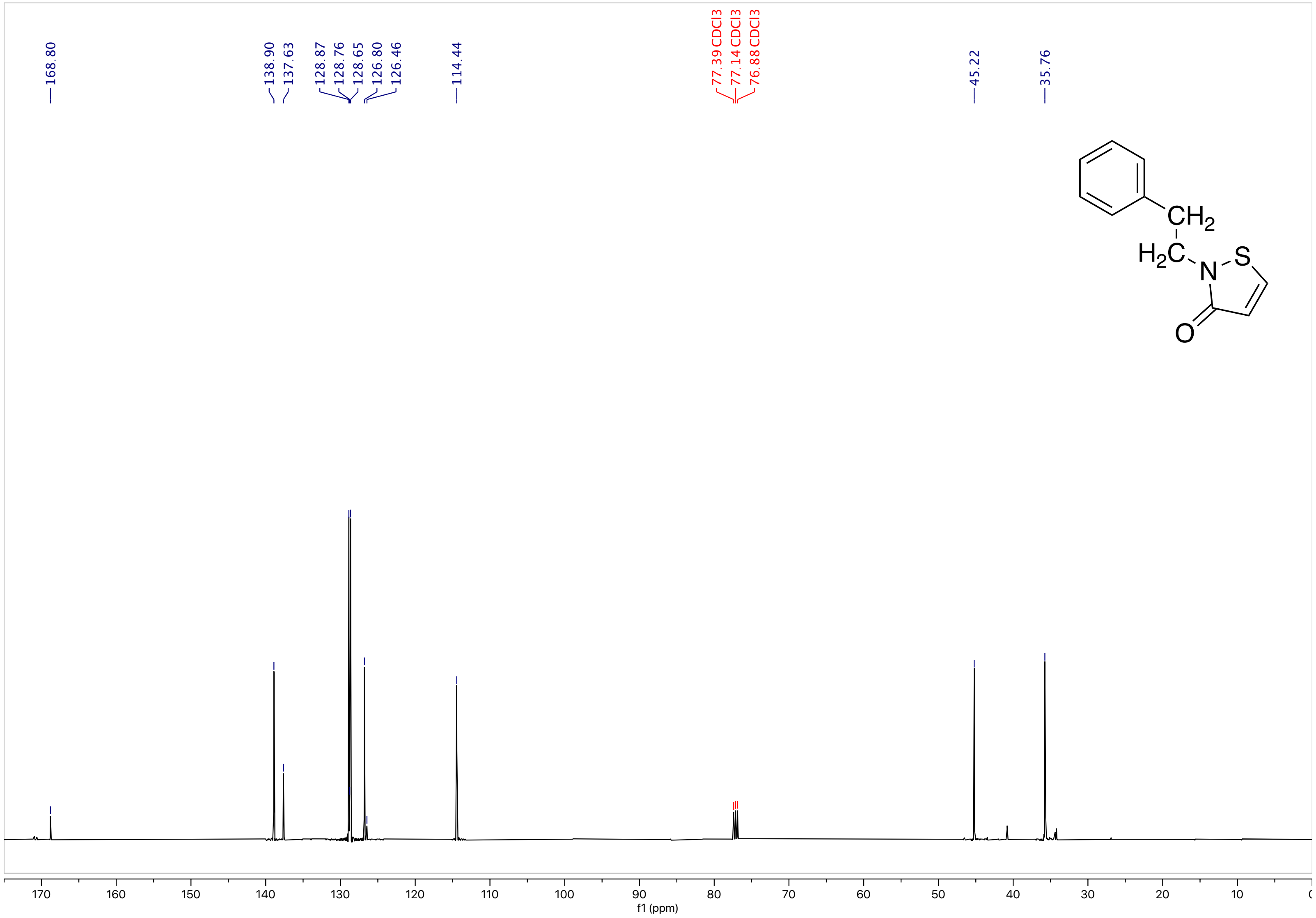

ISFP23 1H NMR

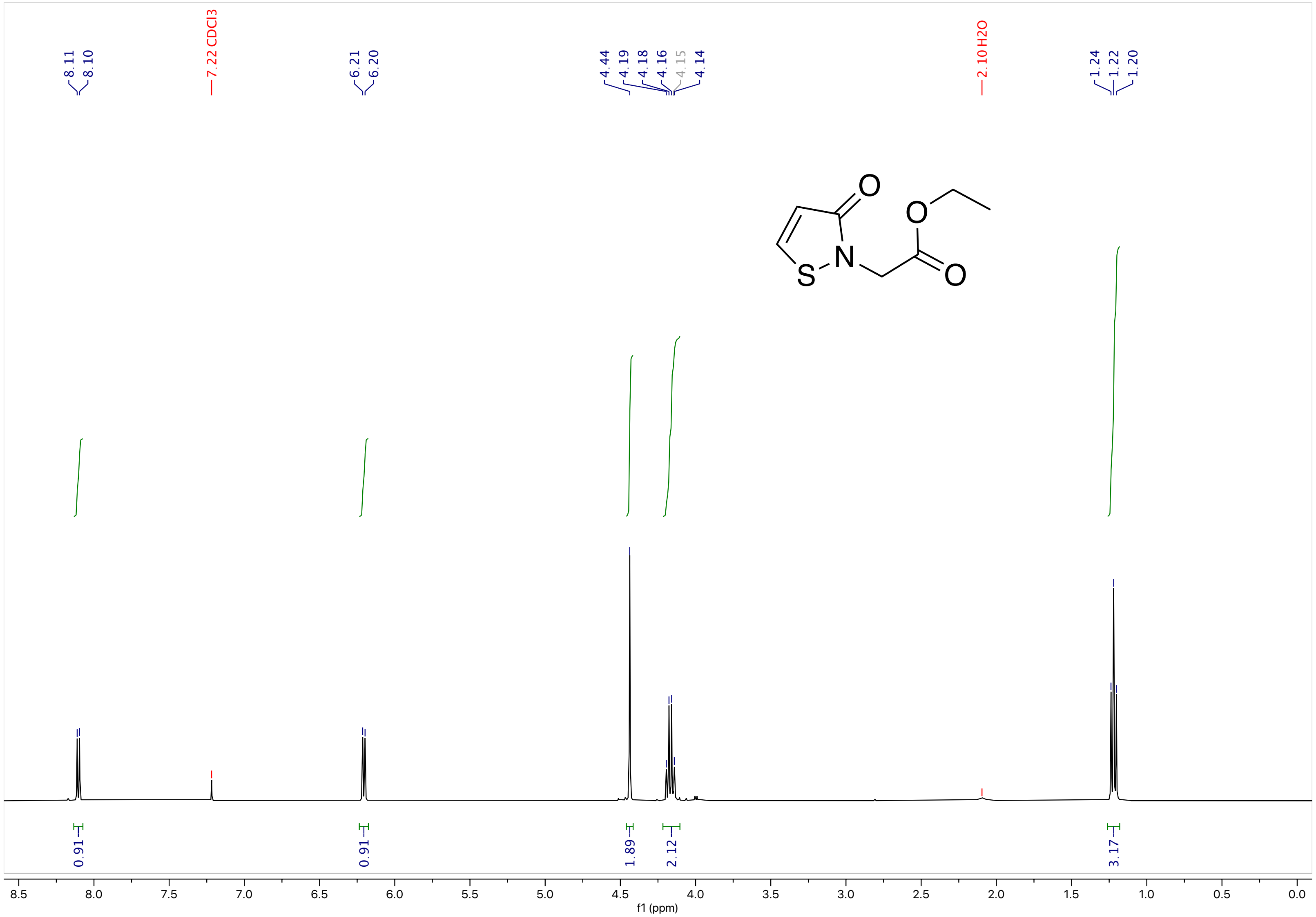

ISFP23 13C NMR

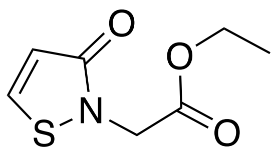

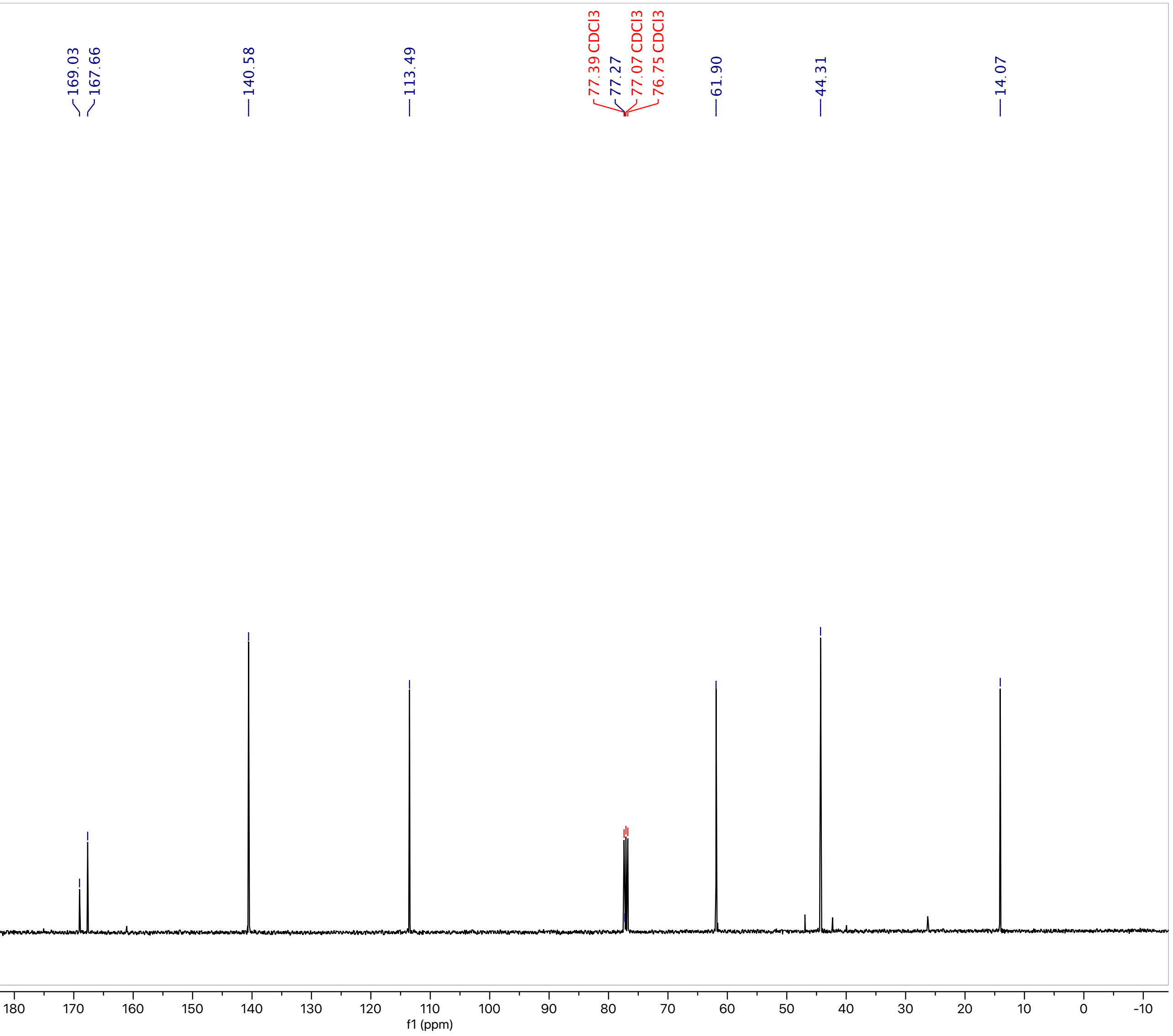

ISFP24 1H NMR

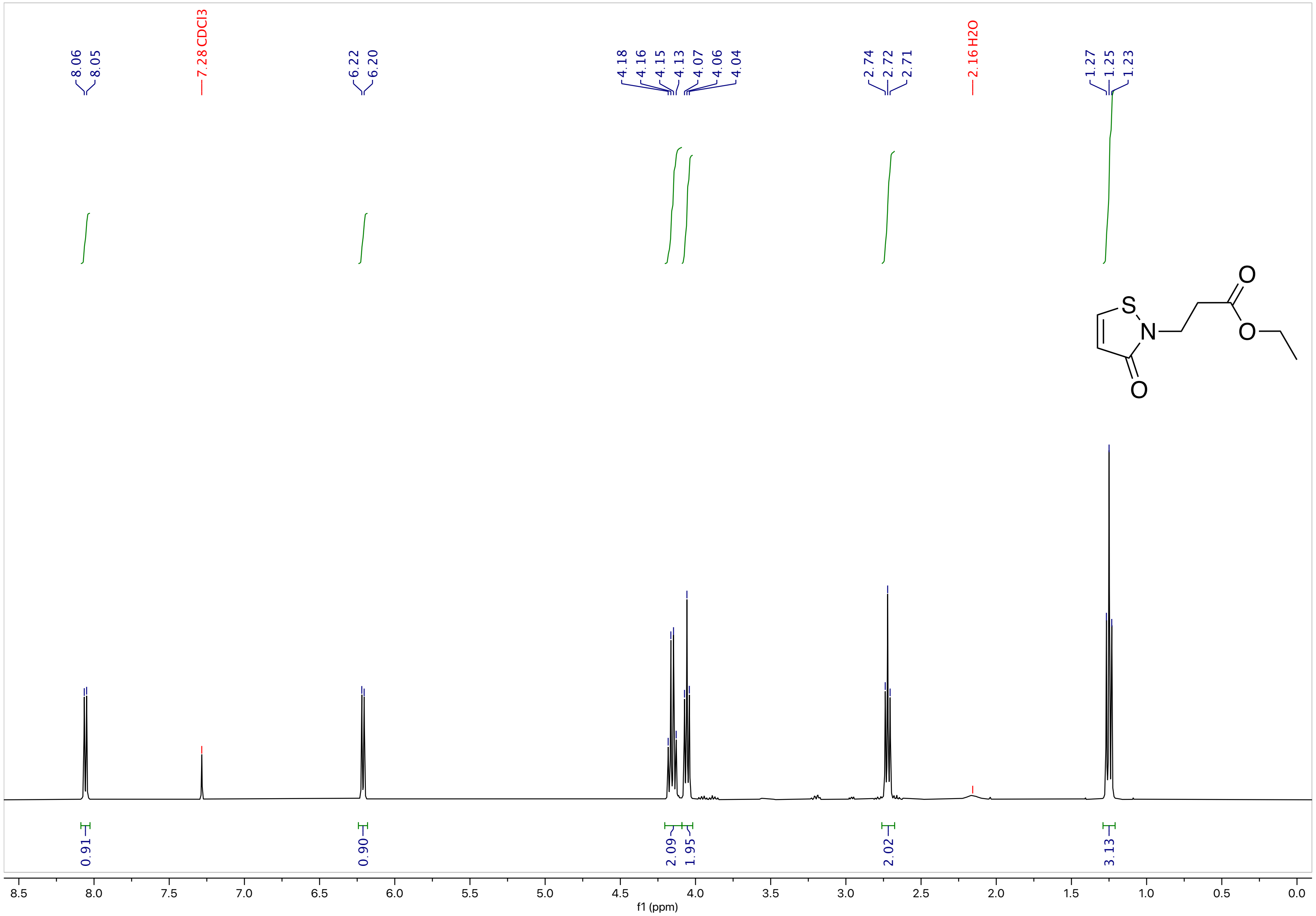

ISFP24 13C NMR

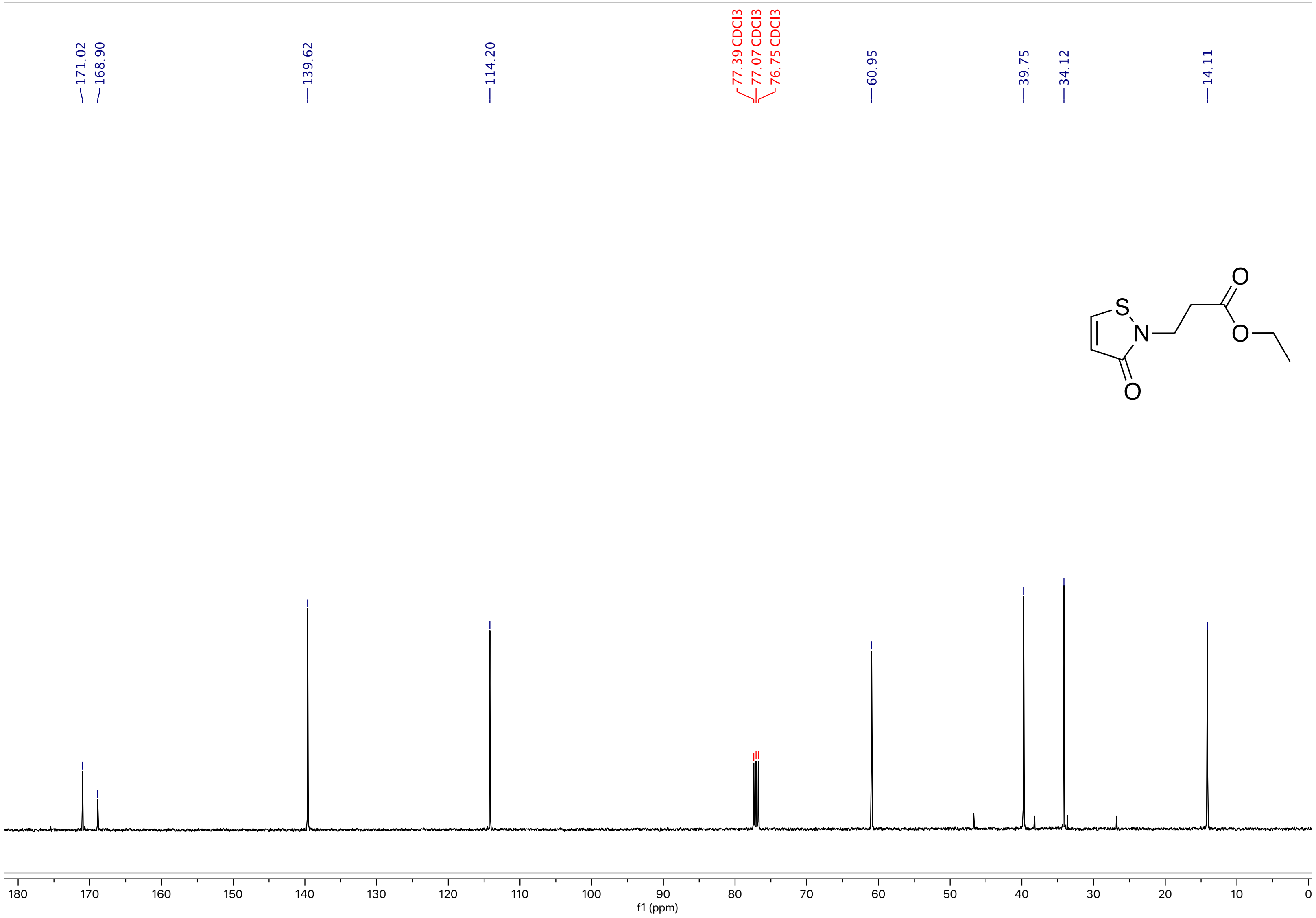

ISFP25 1H NMR

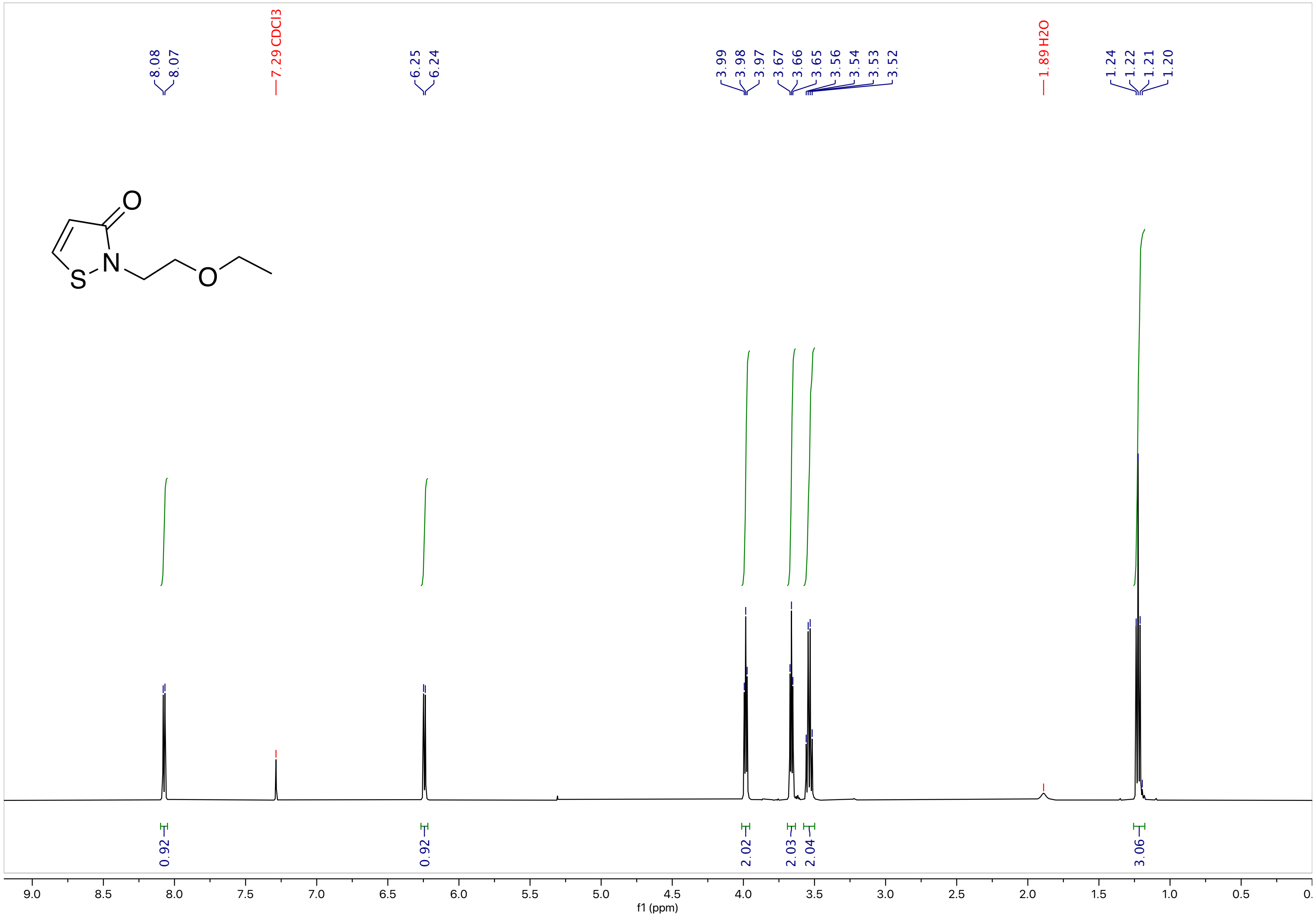

ISFP25 13C NMR

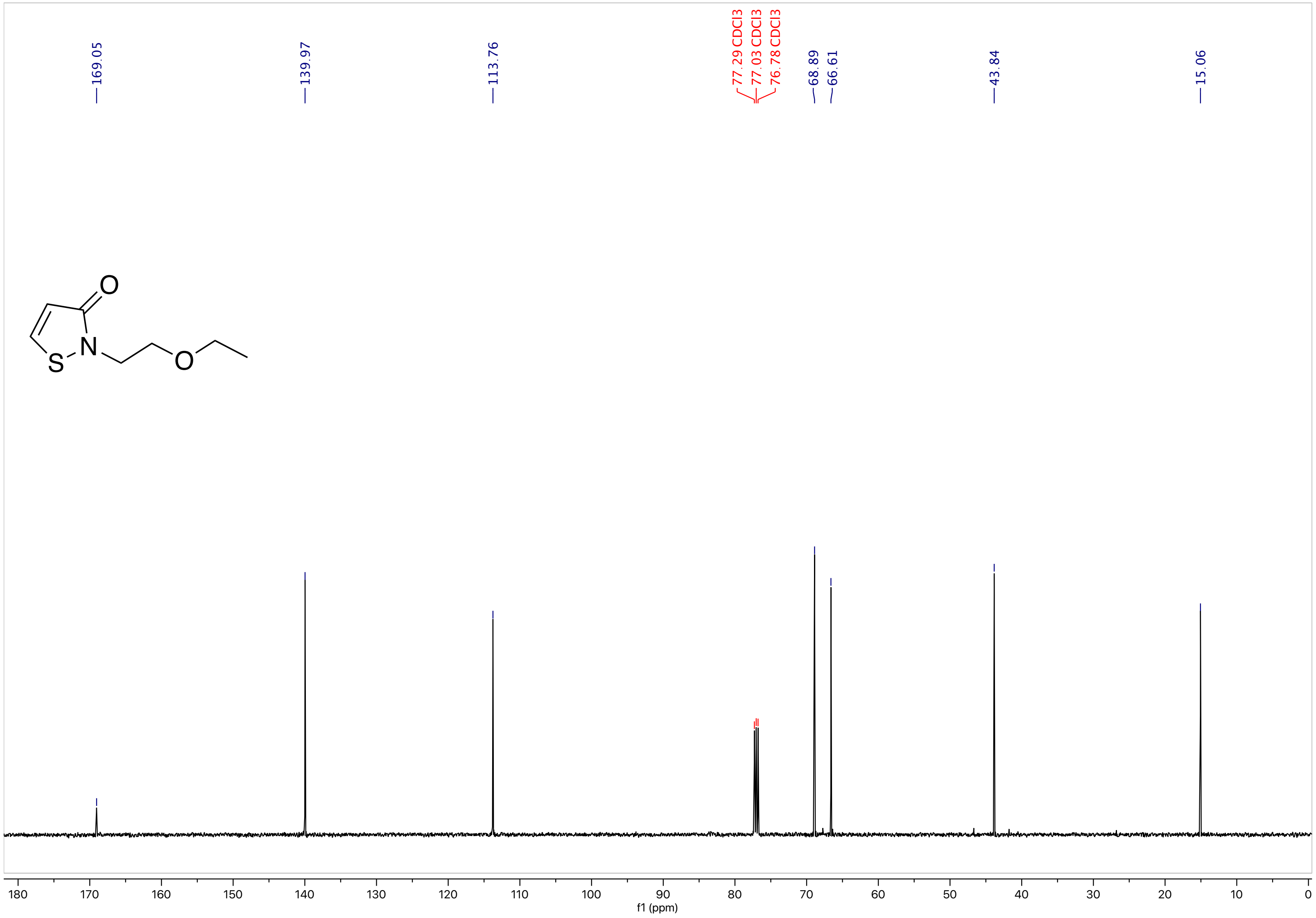

ISFP26 1H NMR

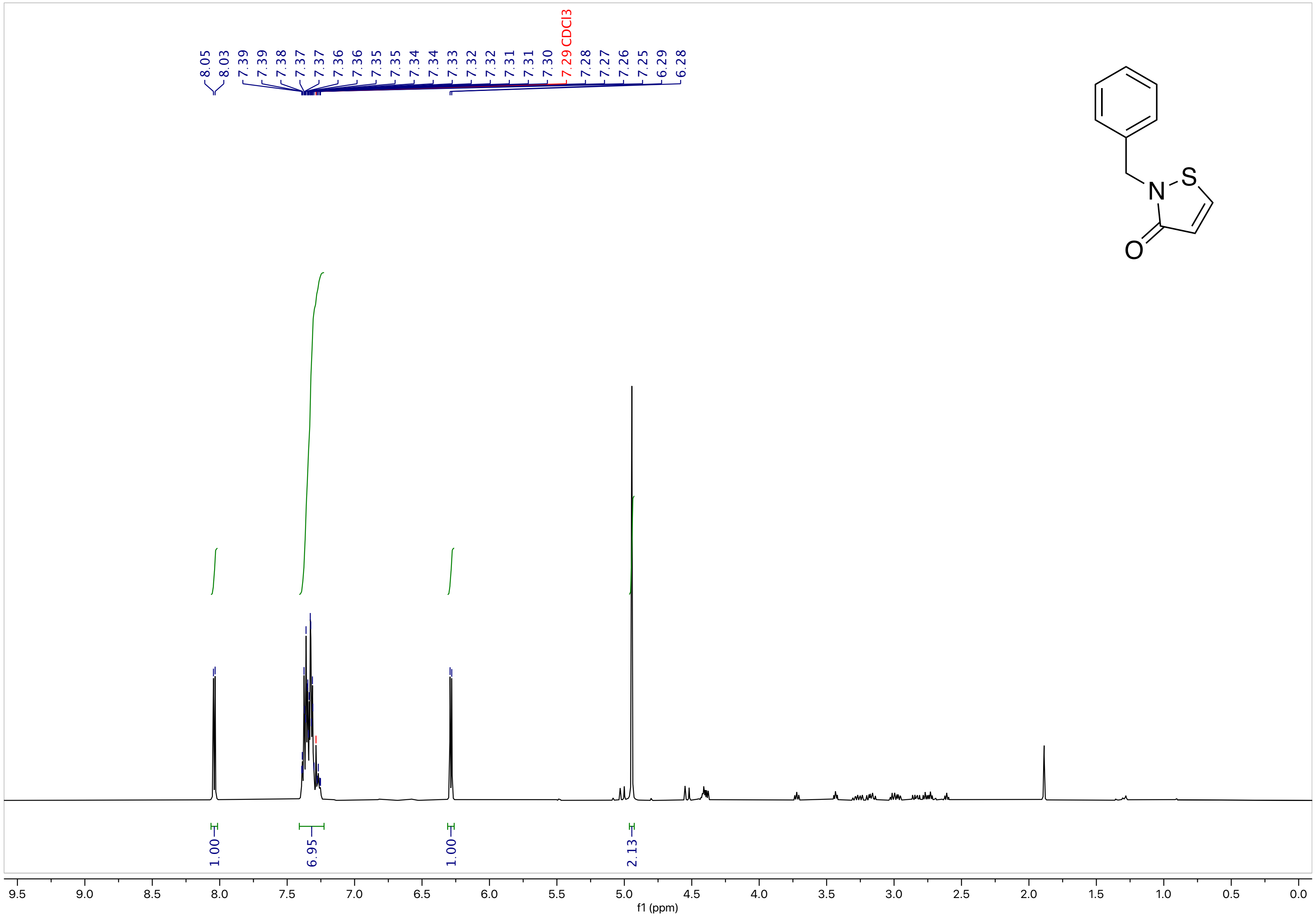

ISFP26 13C NMR

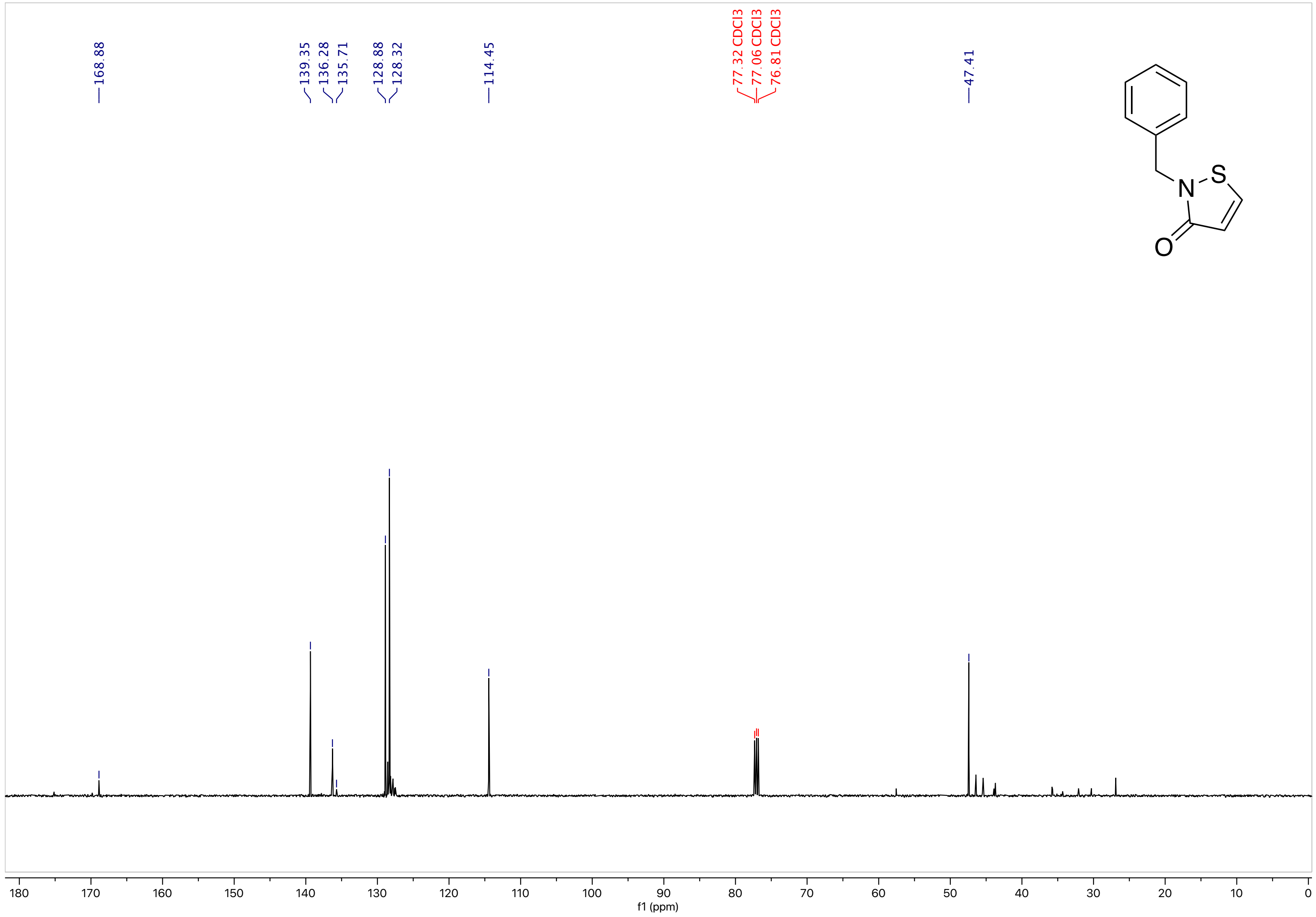

ISFP27 1H NMR

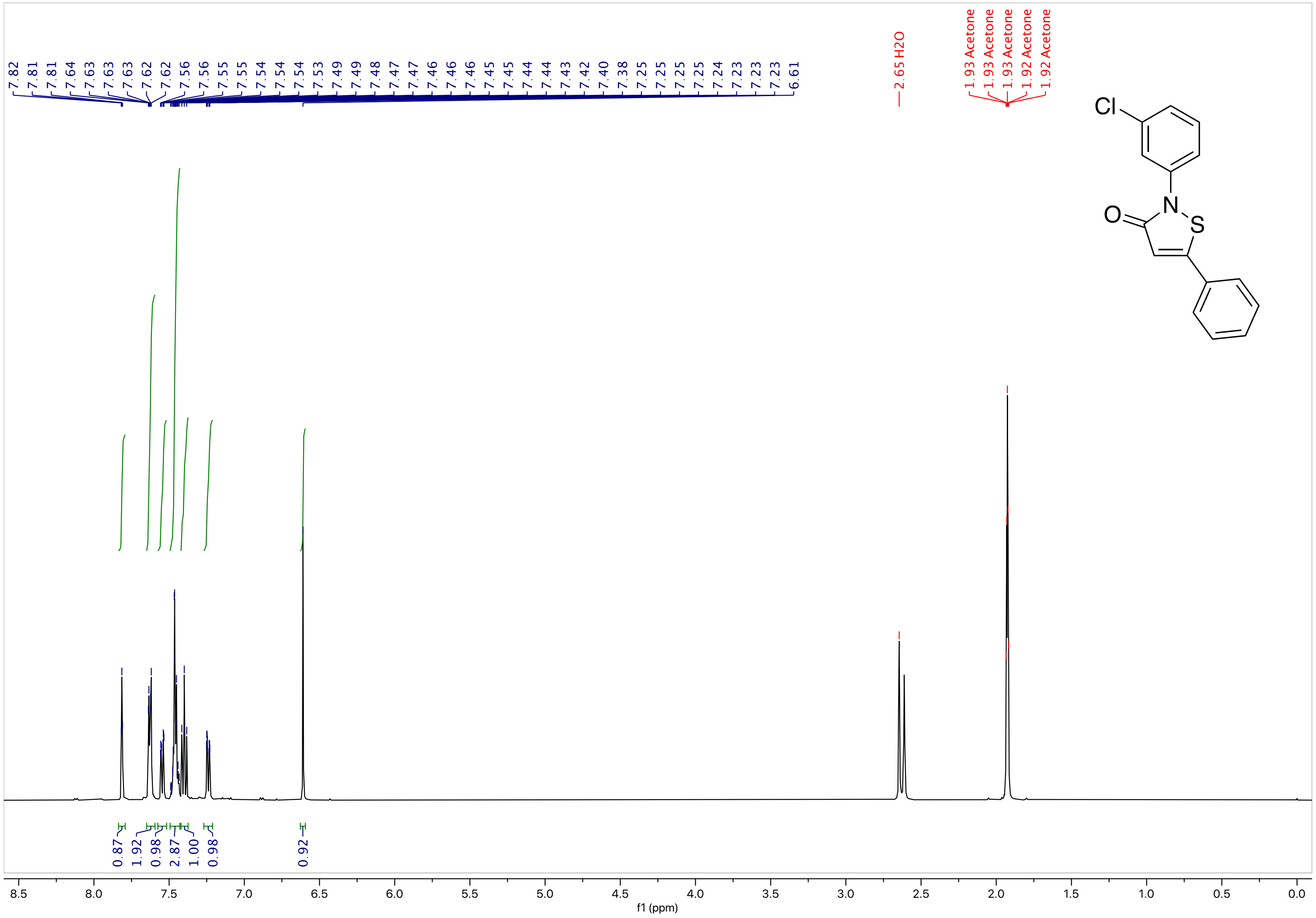

ISFP27 13C NMR

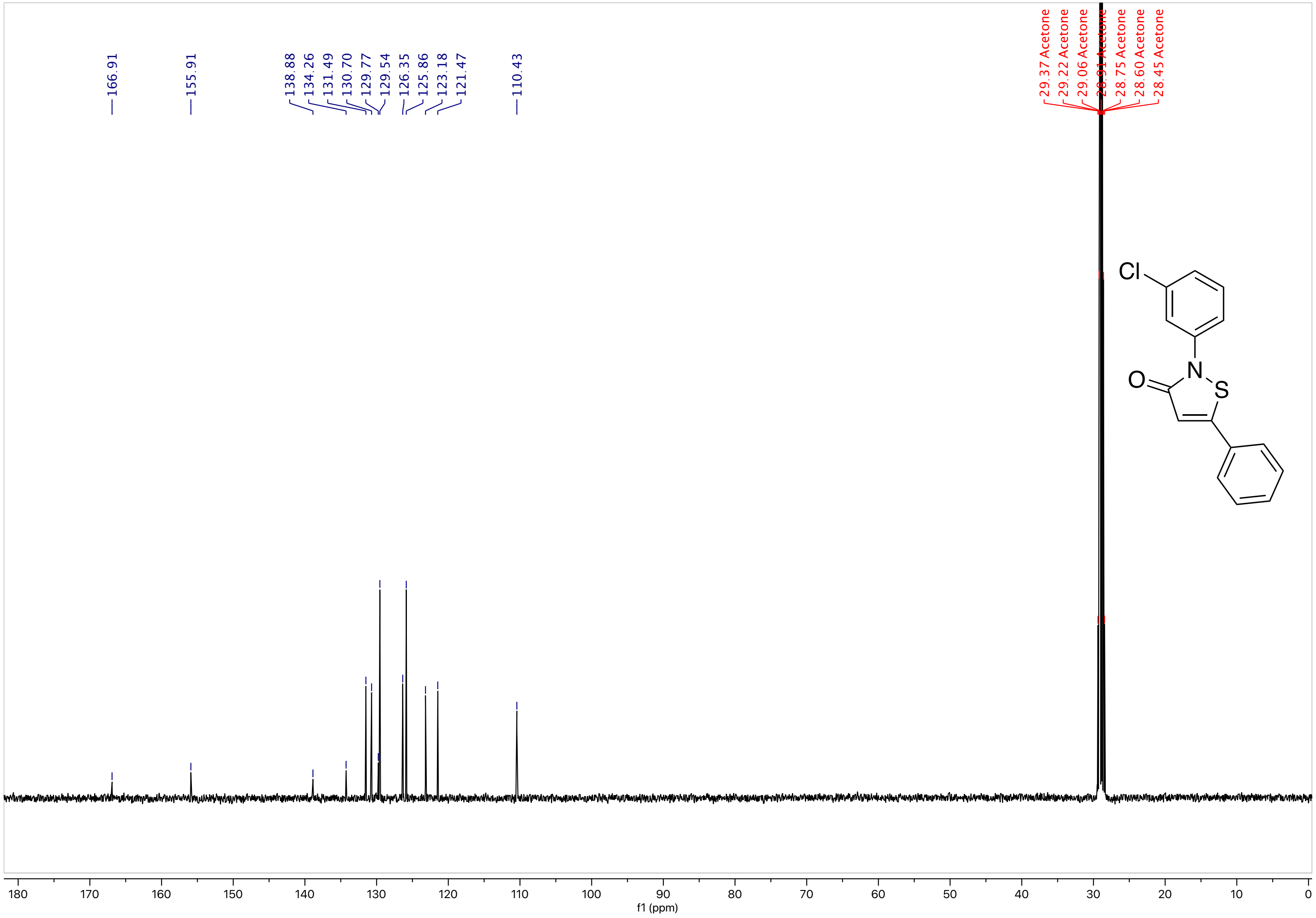

ISFP28 1H NMR

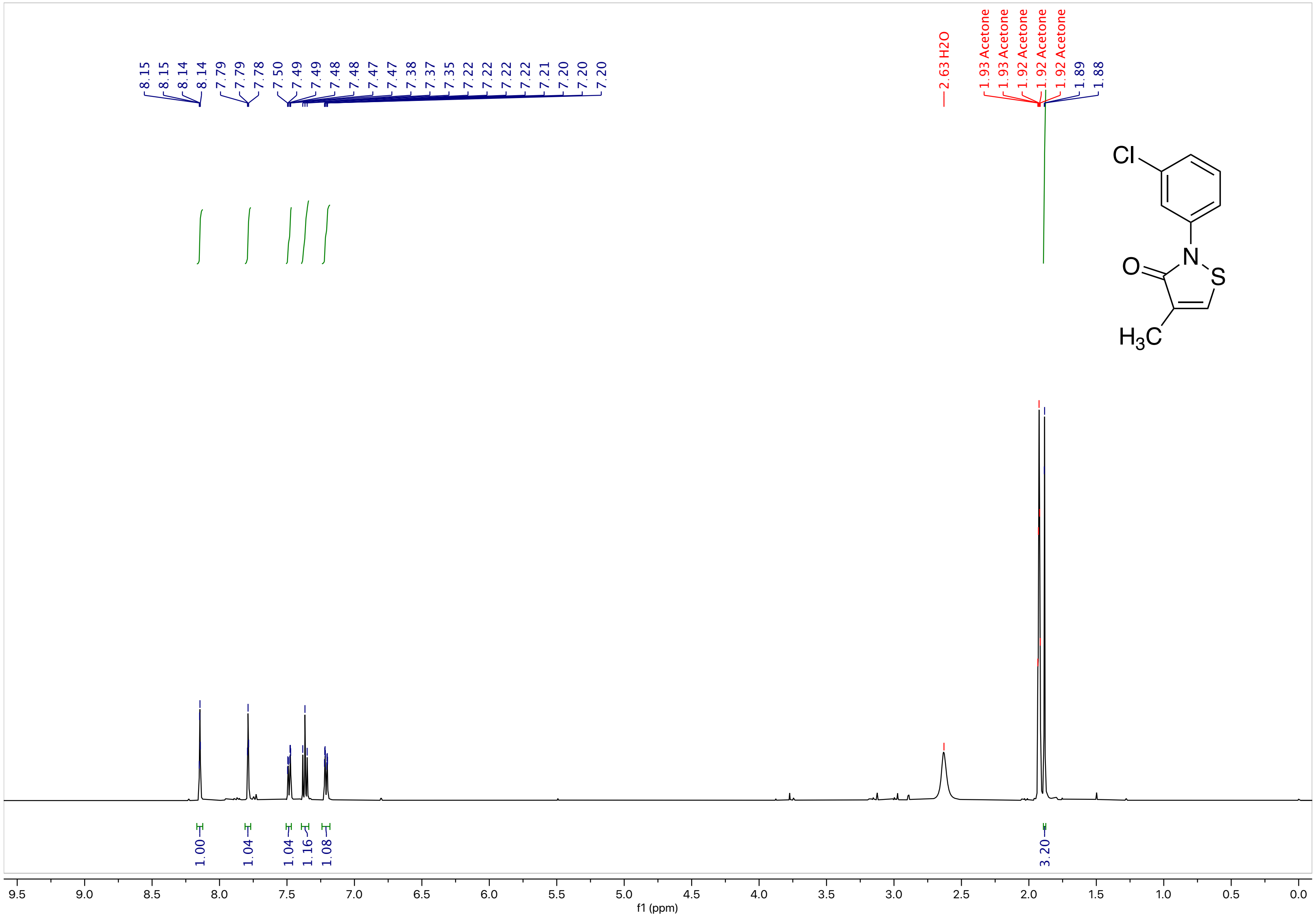

ISFP28 13C NMR

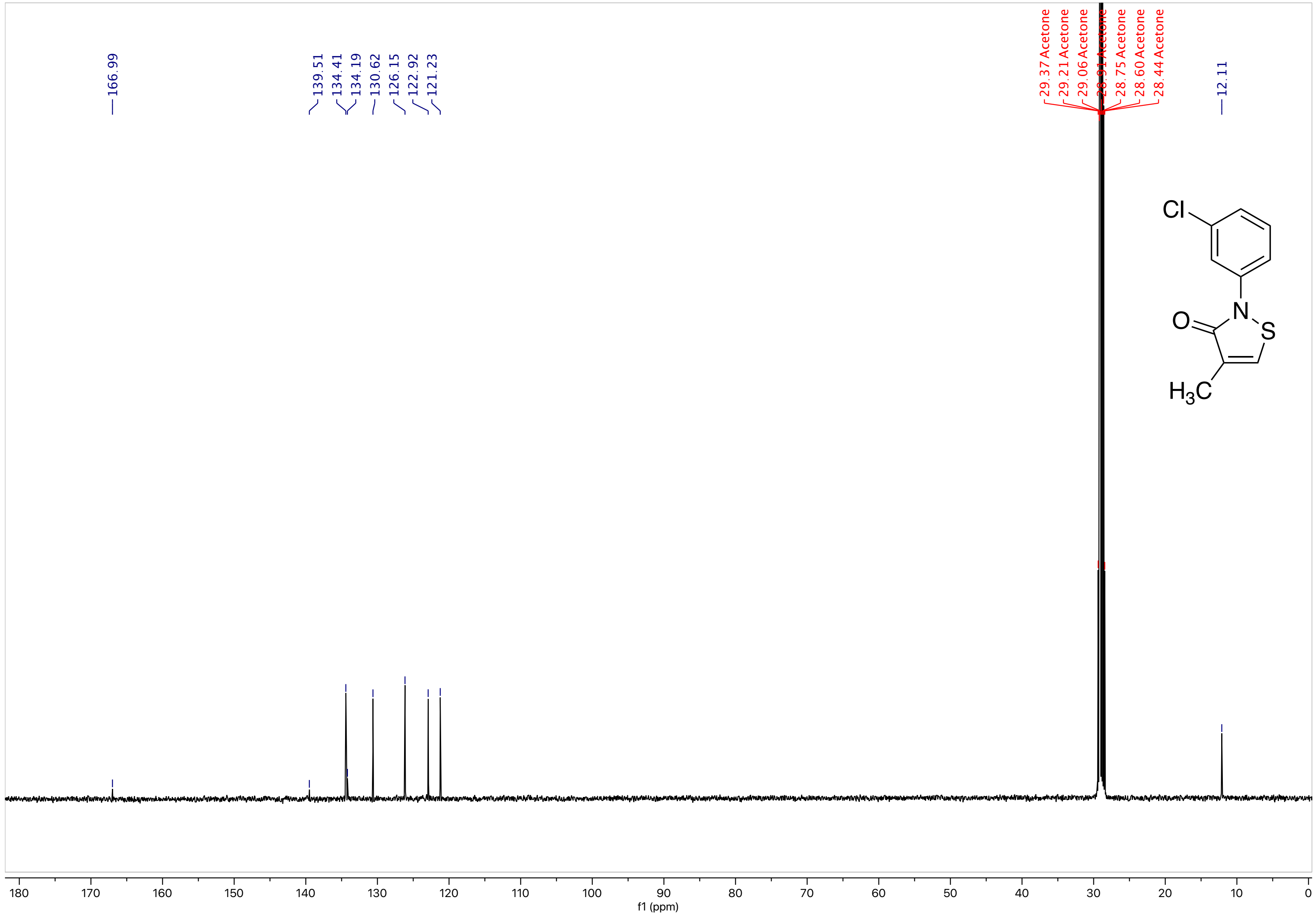

ISFP30 1H NMR

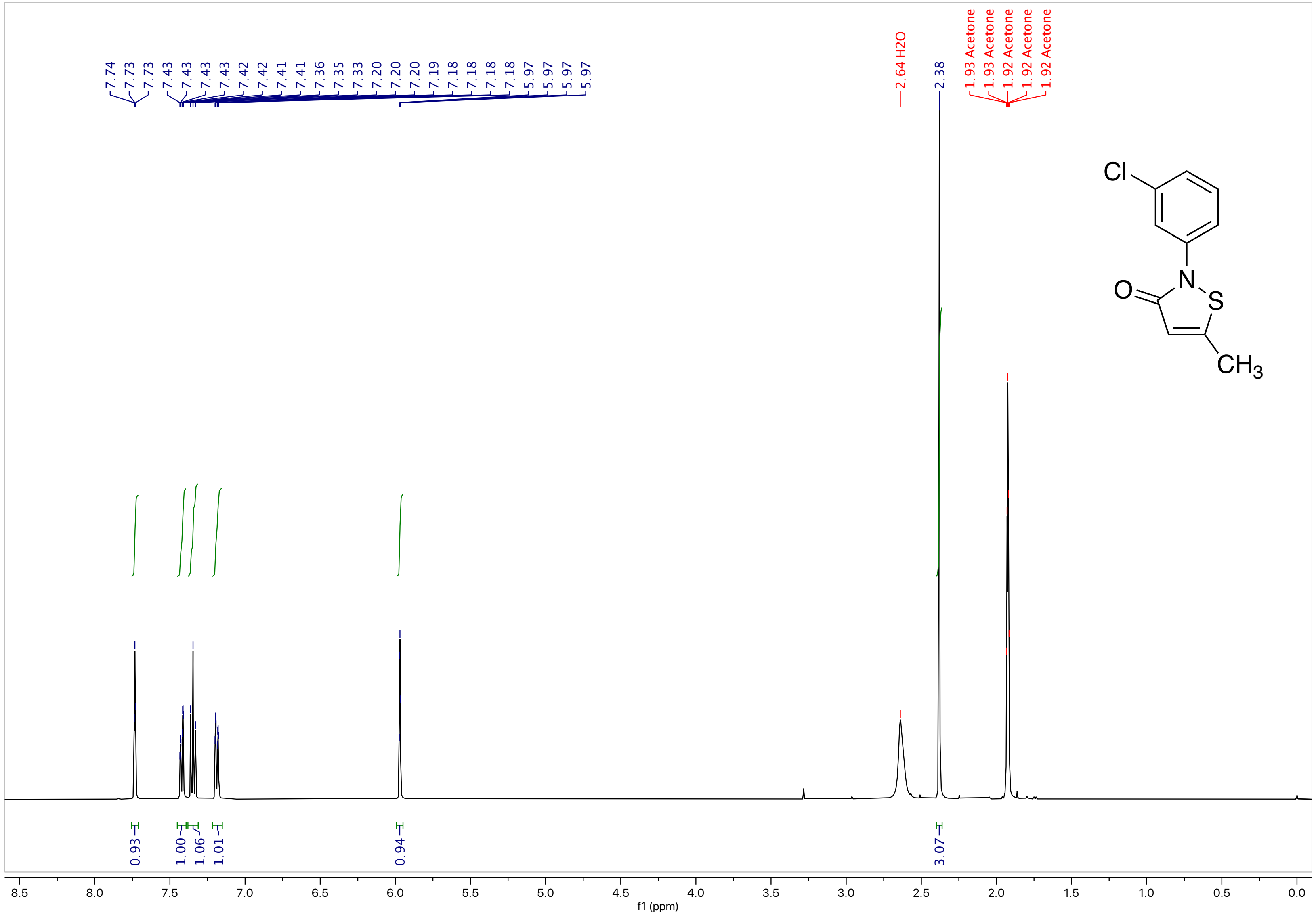

ISFP30 13C NMR

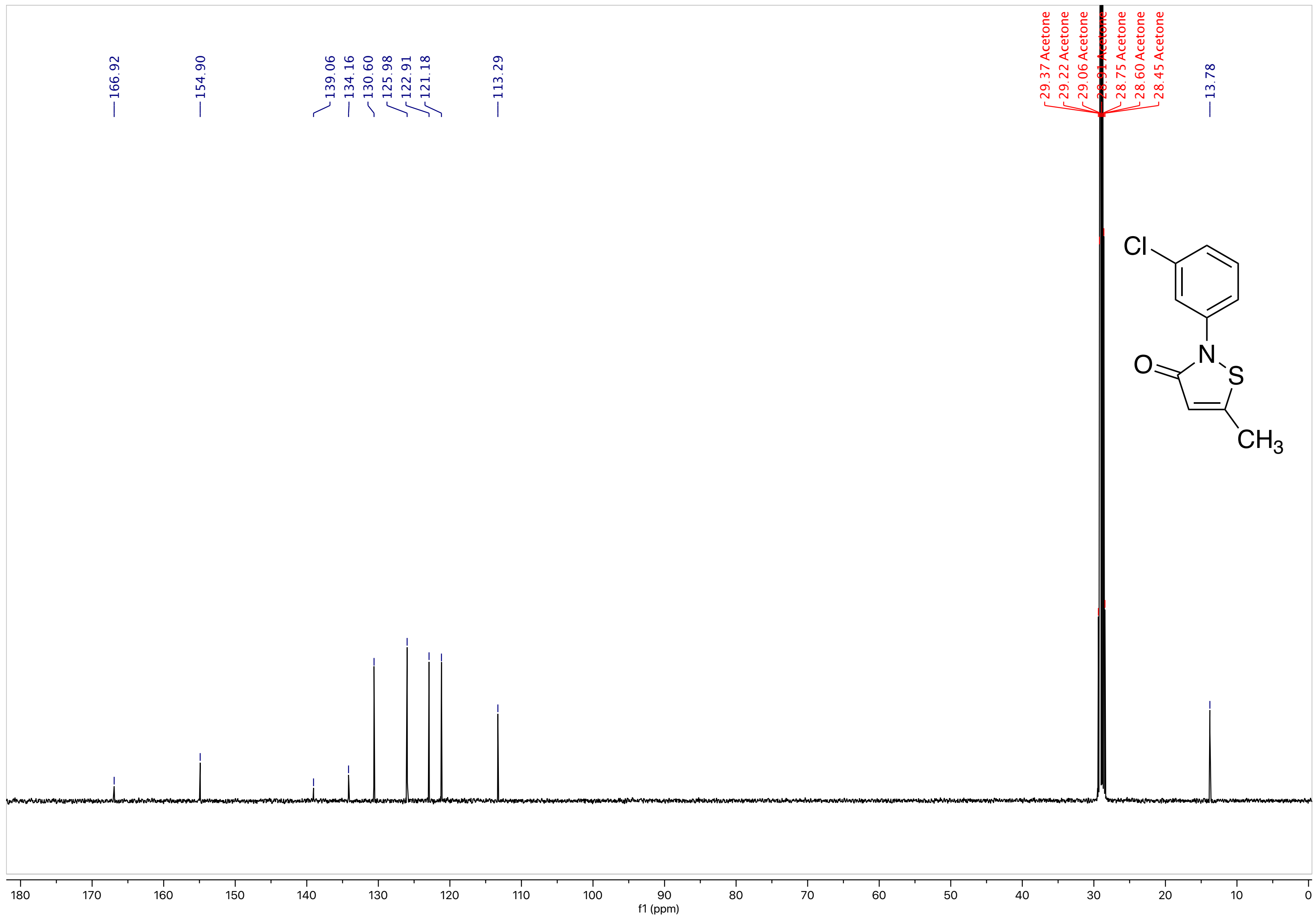

Supplement: NMR_data [file mmc20.pdf]
