# Supplementary material for: Genetic validation of Aspergillus fumigatus phosphoglucomutase as a viable therapeutic target in invasive aspergillosis
Source: J Biol Chem. 2022 Apr 30;298(6):102003. doi: 10.1016/j.jbc.2022.102003 (PMC9168620; doi:10.1016/j.jbc.2022.102003)
Supplement: Figure_S4 [file mmc11.pdf]

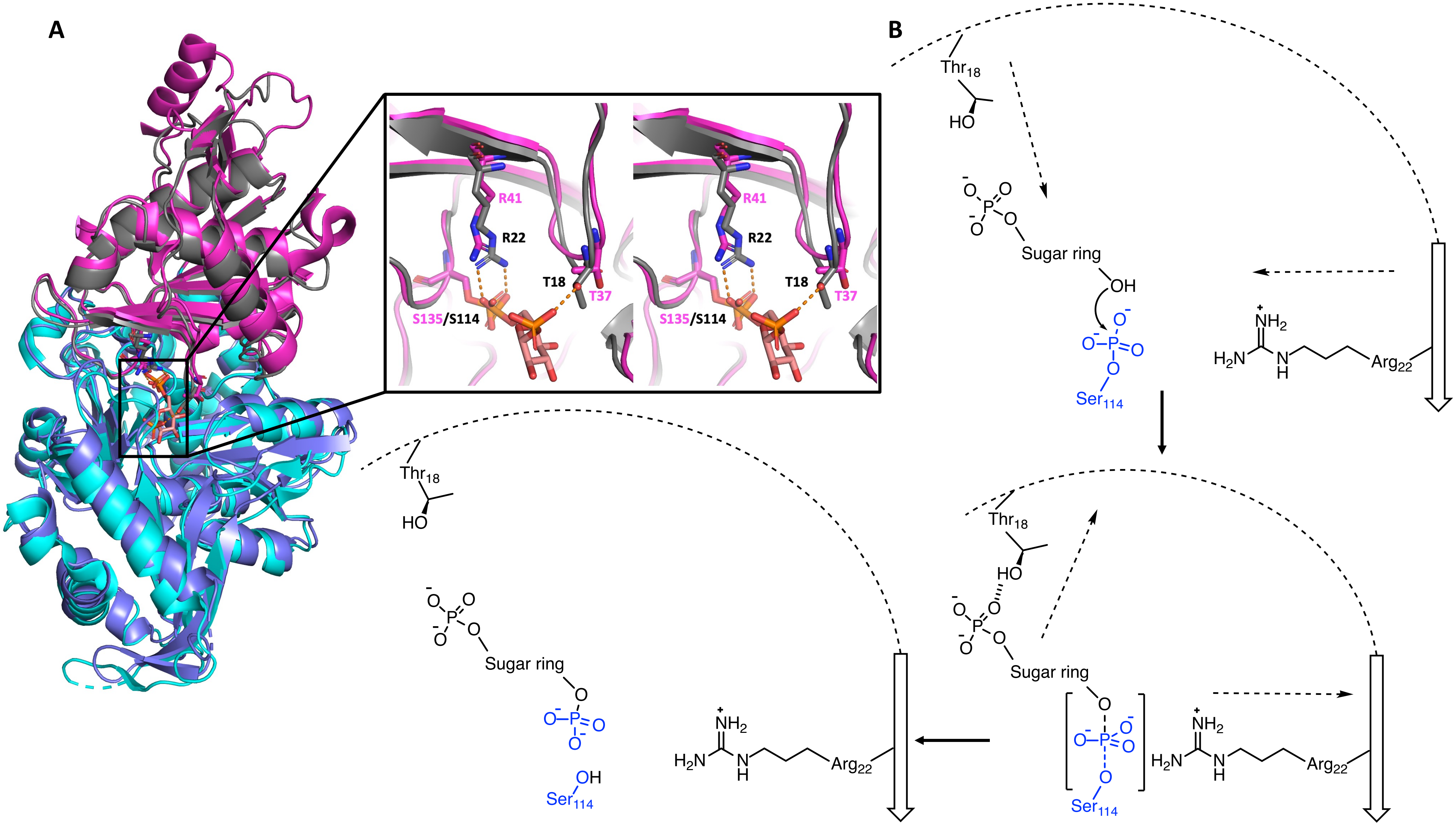
A
R41
R22
T18
T37
S135/S114
B
Thr18
HO
Sugar ring
OH
Ser114
NH2
H2N
N
H
Arg22
O
